# Spatiotemporal Analysis of Mesenchymal Stem Cells Fate Determination by Inflammatory Niche Following Soft Tissue Injury at a Single‐Cell Level

**DOI:** 10.1002/advs.202310282

**Published:** 2024-09-23

**Authors:** Chen Kan, Zhenya Tan, Haitao Wang, Wei Wang, Jiazhao Yang, Ya Zhang, Xiaoling Lu, Qirong Cheng, Lanyi Chai, Chao Peng, Jicheng Zhu, Chenghang Zhu, Hailin Wang, Li Zhan, Keqiong Lin, Yakun Liu, Lingqiang Zhang, Haitao Fan, Hong Zheng

**Affiliations:** ^1^ Department of Pathophysiology School of Basic Medical Sciences Anhui Medical University Hefei 230032 China; ^2^ Department of Physiology and Biomedical Engineering Mayo Clinic Rochester MN 55905 USA; ^3^ Department of Orthopedics The First Affiliated Hospital of USTC Hefei 230001 China; ^4^ State Key Laboratory of Medical Proteomics National Center for Protein Sciences (Beijing) Beijing Institute of Lifeomics Beijing 100850 China; ^5^ Department of Orthopedics The First Affiliated Hospital of Ningbo University Ningbo 315010 China

**Keywords:** heterotopic ossification, inflammatory microenvironment, mesenchymal stem cells, osteoimmunology

## Abstract

Heterotopic ossification (HO), often arising in response to traumatic challenges, results from the aberrant osteochondral differentiation of mesenchymal stem cells (MSCs). Nevertheless, the impact of trauma‐induced inflammatory exposure on MSC fate determination remains ambiguous. In this study, the cellular diversity within inflammatory lesions is elucidated, comprising MSCs and several innate and adaptive immune cells. It is observed that quiescent MSCs transition into cycling MSCs, subsequently giving rise to chondrogenic (cMSC) and/or osteogenic (oMSC) lineages within the inflammatory microenvironment following muscle or tendon injuries, as revealed through single‐cell RNA sequencing (scRNA‐seq), spatial transcriptome and lineage tracing analysis. Moreover, these investigations demonstrate that neutrophils and natural killer (NK) cells enhance transition of quiescent MSCs into cycling MSCs, which is also controlled by M1 macrophages, a subpopulation of macrophages can also stimulate cMSC and oMSC production from cycling MSCs. Additionally, M2 macrophages, CD4^+^ and CD8^+^ T lymphocytes are found to promote chondrogenesis. Further analysis demonstrates that immune cells promotes the activation of signaling transducers and activators of transcription (STAT) pathway and phosphoinositide 3 (PI3K)/protein kinase B (AKT) pathway in MSC proliferation and osteochondral progenitors’ production, respectively. These findings highlight the dynamics of MSC fate within the inflammatory lesion and unveil the molecular landscape of osteoimmunological interactions, which holds promise for advancing HO treatment.

## Graphical abstract

The dynamics of mesenchymal stem cell (MSC) lineage specification and associated dynamic regulation is highlighted by immune cells during HO formation, i.e., an elaborate trajectory of MSCs is characterized during aberrant osteochondrogenesis and demonstrates that MSCs proliferation are regulated by M1 macrophages (M1), neutrophils, and natural killer (NK) cells via janus kinase (JAK)/signal transducers and activators of transcription (STAT) signaling and differentiation process, including chondrogenic MSC (cMSC) and osteogenic MSC (oMSC) production are determined by M2 macrophages (M2), CD4^+^ and CD8^+^ T cells via phosphoinositide 3 kinase (PI3K) /protein kinase B (AKT) signaling activation.

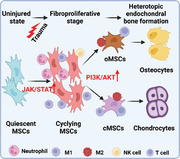

## 1 Introduction

Heterotopic ossification (HO), whether triggered by trauma or hereditary factors, represents the abnormal formation of bone within soft tissues, including muscle and tendons.^[^ ^1^, ^2^, ^3^ ^]^ Traumatic HO (tHO) is a frequent and debilitating consequence of traumatic incidents such as tendon injuries, burns, spinal cord injuries, and traumatic brain injuries.^[^ ^4^, ^5^ ^]^ Consistently, an acute trauma can also exacerbate the development of fibrodysplasia ossificans progressiva (FOP), which is a genetic form of HO caused by activating mutations in Activin A receptor type 1 (*ACVR1*).^[^ ^6^ ^]^ Patients afflicted with HO often experience chronic pain, restricted joint mobility, diminished quality of life, and impose a significant clinical burden.^[^ ^4^ ^]^ Yet, our comprehension of the pathophysiological mechanisms underlying HO remains incomplete, and effective treatment options are presently lacking.

Trauma‐induced hyperactive inflammation is acknowledged as a central driver of abnormal tissue repair during HO formation.^[^ ^7^, ^8^, ^9^ ^]^ Immune cells are recruited to the site of injury, where they interact with tissue‐resident stem cells within the inflammatory microenvironment, initiating tissue repair.^[^ ^10^ ^]^ Macrophages, neutrophils, and lymphocytes have been implicated in HO formation.^[^ ^11^, ^12^, ^13^, ^14^, ^15^ ^]^ The diversity of these versatile immune cells warrants further investigation, and their impact on stem cell function in HO remains largely elusive.

Mesenchymal stem cells (MSCs), also known as multipotent stem cells or mesenchymal stromal cells, are characterized by their tri‐lineage differentiation potential, encompassing osteogenesis, chondrogenesis, and adipogenesis.^[^ ^16^ ^]^ Several mesenchymal lineage cells, including glioma‐associated oncogene homolog 1 (*Gli1*)^+^, tyrosine kinase receptor 2 (*Tie2*)^+^, mx dynamin like GTPase 1 (*Mx1*)^+^, homeobox protein A11 (*Hoxa11*)^+^ and cathepsin K (*Ctsk*)^+^ cells, undergo aberrant osteochondral differentiation and are recognized as the cellular origin of HO.^[^ ^17^, ^18^, ^19^ ^]^ MSCs exhibit considerable heterogeneity^[^ ^20^ ^]^ Lineage tracing analysis, employing a fluorescence reporter system in mice, can track the terminal differentiation of MSCs but does not provide insights into their spatiotemporal changes in gene expression and function. Consequently, the developmental trajectory of MSCs in HO formation remains largely obscure. Additionally, further investigation is warranted to delineate the underlying mechanisms, with a focus on identifying potential therapeutic targets.

In this study, we employed scRNA‐seq in tandem with lineage tracing assays to unravel the developmental trajectory of MSCs in neuron specific enolase (*Nse*)‐bone morphogenetic protein 4 (*Bmp4*) mice, which is an animal model of FOP. Subsequent analysis using spatial transcriptomics unveiled the presence of these MSCs within the inflammatory niche, alongside macrophages, neutrophils, NK cells, and T cells. Several ligands expressed by these immune cells have the potential to promote MSC proliferation by activating the STAT signaling pathway and MSC differentiation through the PI3K/AKT pathways. In sum, our results provide an in‐depth understanding of the development and diversity of MSCs and immune cells, shedding light on their interactions and associated signaling pathways. These insights offer a novel perspective on the interplay between osteoimmunology and HO treatment.

## 2 Results

### 2.1 Characterization of Cell Types in the Inflammatory Lesion of Injured Tibial Muscle of BMP4‐Dependent HO Model Mice

**Figure 1:**
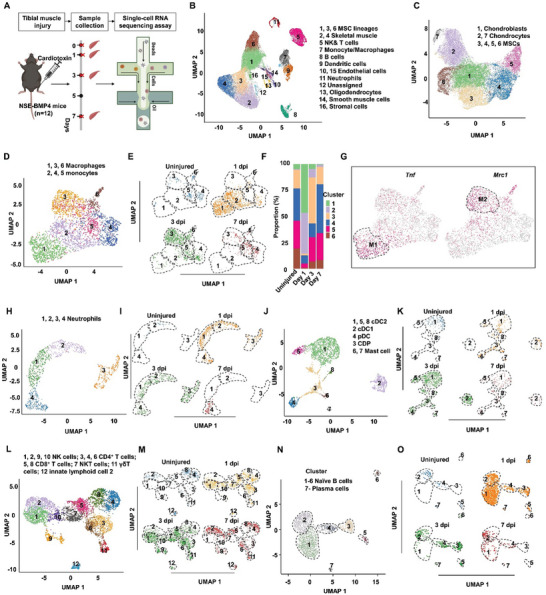
scRNA‐seq analysis of the cell types in the injured sites of tibial muscle from HO model mice. A) Schematic of the study design, highlighting the systematic process of target tissue collection at specified time intervals for scRNA‐seq analysis. B) UMAP visualization of 50 466 cells derived from injured tibial muscle of *Nse‐Bmp4* mice. C) UMAP visualization of MSC linage cells in injured tibial muscle of *Nse‐Bmp4* mice. D) UMAP visualization of macrophages and monocytes derived from injured tibial muscle of *Nse‐Bmp4* mice. E) UMAP visualization and F) percentage of macrophages and monocytes in uninjured and injured tibial muscle of *Nse‐Bmp4* mice at different time point. G) The feature plot images of classical molecules for M1 and M2 macrophages in uninjured and injured tibial muscle of *Nse‐Bmp4* mice covering 4 time points. H,I) UMAP visualization of neutrophils derived from tibial muscle of H) *Nse‐Bmp4* mice covering 4 time points and I) different time points with or without injury. UMAP visualization of DCs derived from tibial muscle of J) *Nse‐Bmp4* mice covering 4 time points and K) different time points post injury. L,M) UMAP visualization of NK & T cells derived from tibial muscle of L) *Nse‐Bmp4* mice covering 4 time points and M) different time points post injury. UMAP visualization of B cells derived from N) tibial muscle of *Nse‐Bmp4* mice covering 4 time points and O) different time points post injury.

Inflammation can drive MSC differentiation into osteochondral lineage cells, resulting in formation of HO in soft tissues, such as skeletal muscle and tendon.^[^ ^21^, ^22^, ^23^, ^24^ ^]^ To investigate the progression of inflammation‐related changes in MSC fate determination, we established a murine model of injury‐induced HO, as previously reported, through cardiotoxin (CTX) injury of tibial muscle in *Nse‐Bmp4* mice,^[^ ^7^ ^]^ which showed no significant difference in the percentage of immune cells and MSCs comparing with uninjured WT mice and could spontaneously develop HO with age (Figures S1A‐C and S2A,B, Supporting Information). We then examined HO development and MSC differentiation at different time points post injury (*i.e*., 1‐, 3‐ and 7‐days post injury) (Figure S3A‐C, Supporting Information). Next, injured and matched uninjured tibial muscles were harvested at 1‐, 3‐, and 7‐days post injury (dpi) for single cell RNA‐sequencing (scRNA‐seq) using the 10× genomics platform (**Figure** **1**A). This analysis generated a final dataset obtained of 50466 cells after quality control and batch integration (Figure S4A‐H, Supporting Information) and further confirmed that skeletal muscle‐derived BMP4 could regulate HO formation in *Nse*‐*Bmp4* mice (Figures S5 and S6, Supporting Information). We then defined 12 cell types by combining markers obtained from the Immgen database with previously published markers^[^ ^25^ ^]^ to improve resolution of subtypes: MSC lineage cells (clusters 1, 3 and 6, *Prrx1* ^+^/*Col1a1* ^+^/*Ptprc* ^−^), skeletal muscle cells (clusters 2 and 4, *Sgce* ^+^/*Col4a3* ^+^/*Myl9* ^+^/*Ptprc* ^−^), NK & T cells (cluster 5, *Klrb1c* ^+^ or *Cd3e* ^+^), monocytes/macrophages (cluster 7, *Cd68* ^+^), B cells (cluster 8, *Cd19* ^+^), Dendritic cells (DC) (cluster 9, *Itgax* ^+^), endothelial cells (clusters 10 and 15, *Cdh5* ^+^ or *Prox1* ^+^), neutrophils (cluster 11, *Ly6g* ^+^), oligodendrocytes (cluster 13, *Mbp* ^+^), smooth muscle cells (cluster 14, *Acta2* ^+^/*Pdgfra* ^−^), and stromal cells (cluster 16, *Kera* ^+^) (Figure 1B and Figure S7A,B, Supporting Information).

We next examined the diversity of MSC lineage cells and immune cells during HO development. UMAP was used to identify 7 subtype clusters within the mesenchymal lineage cluster based on signature gene expression (Figure 1C and Figure S8A, Supporting Information). More specifically, this analysis identified MSCs among MSC lineage cells in clusters 3, 4, 5 and 6 based on their differential expression of *Pdgfra, Prrx1, Ly6a*, *Ly6e*, *Cxcl12* and *Adam12* (Figure 1C and Figure S8B, Supporting Information). Additionally, MSC lineage cells in cluster 1 also expressed *Tnc*, suggesting that these were chondroblasts, while those in clusters 2 and 7 showed higher expression of *Col2a1* and *Col10a1*, indicating they were mature chondrocytes (Figure 1C and Figure S8B, Supporting Information).

Additionally, we characterized the innate immune cells in inflammatory lesions via Immgen database and expression of recognized markers, including 6 clusters of monocytes/macrophages (Figure 1D and Figure S9A, Supporting Information). Clusters 1, 3 and 6 were macrophages, while clusters 2, 4, and 5 were monocytes (Figure 1D and Table S1, Supporting Information). Notably, cluster 1 macrophages were detected at the injury site immediately following injury (most prevalent in 1dpi samples) and most likely promoted inflammatory response through higher expression of *Tnf*, *Fcgr1*, *Tlr4* and *Il1a* compared to other‐cluster macrophages, suggesting M1 subtype^[^ ^7^, ^26^ ^]^ (Figure 1E–G and Figure S9B–E, Supporting Information). Conversely, cluster 3 appeared to be recruited later (highest abundance at 3 dpi) and tissue‐resident cluster 6 (prevalent in uninjured tissue) were both enriched for *Mrc1*, *Csf1r*, *Tgfb1*, *Cd163* and *Retnla* expression, implying an anti‐inflammatory role characteristic of M2 macrophages (Figure 1G and Figure S9F–I, Supporting Information).^[^ ^7^ ^]^ We also identify 4 clusters of neutrophils (Figure 1H,I and Figure S10A, Supporting Information), and characterized 4 DC subtypes: cDC1 (clusters 2, *Itgax* ^+^/*Xcr1* ^+^), cDC2 (clusters 1, 5 and 8, *Itgax* ^+^/*Cd209a* ^+^), pDC (clusters 4, *Itgax* ^+^/*Cd4* ^+^) and common DC progenitors (CDP, cluster 3, *Itgax* ^−^/*H2‐Aa* ^+^/*Flt3* ^+^/*Csf1r* ^+^/*Adgrg3* ^+^/*Sox4* ^+^) (Figure 1J,K and Figure S10B,C, Supporting Information).^[^ ^27^ ^]^ Clusters 6 and 7 expressed *Ms4a2* and were therefore considered mast cells (Figure S10C, Supporting Information).^[^ ^28^ ^]^

Exploration of diversity among adaptive immunity cell populations showed that clusters 1, 2, 9 and 10 were likely NK cells (*Klrb1c* ^+^/*Ncr1* ^+^), clusters 3, 4 and 6 were CD4^+^ T cells (*Cd3e* ^+^/*Cd4* ^+^), clusters 5 and 8 were CD8^+^ T cells (*Cd3e* ^+^/*Cd8a* ^+^), cluster 7 comprised NKT cells (*Klrb1c* ^+^/*Cd3e* ^+^), cluster 11 was γδT cells (*Cd3e* ^+^/*Klrb1c* ^−^/*Cd4* ^−^/*Cd8a* ^−^), and cluster 12 included innate lymphoid cells 2 (*Gata3* ^+^/*Klrb1c* ^−^/*Cd3e* ^−^) (Figure 1L,M and Figure S11A,B, Supporting Information). More specifically, cluster 3 CD4^+^ T cells expressed *Foxp3* at high levels, identifying them as Tregs (Figure 1L and Figure S11C, Supporting Information). Cluster 6 CD4^+^ T cells expressed *Tbx21*, indicating these were the Th1 population, and cluster 4 were considered naïve CD4^+^ T cells (Figure 1L and Figure S11C, Supporting Information). Cluster 8, which was positive for *Ccr7*, were potentially naïve CD8^+^ T cells, whereas cluster 5 was considered cytotoxic effector T cells based on their *Tbx21* expression (Figure 1L and Figure S11C, Supporting Information). In addition, we characterized 2 subtypes of B cells: naïve B cells (clusters 1‐6, *Cd19* ^+^ /*Ighd* ^+^/Cd27^−^) and plasma cells (clusters 7, *Tnfrs17* ^+^)^[^ ^29^ ^]^ (Figure 1N,O and Figure S12, Supporting Information).

Moreover, we performed an integrated scRNA‐seq analysis combining WT and *Nse*‐*Bmp4* mice of C57BL/6 background, including both uninjured and injured groups. GO analysis revealed that there were a remarkable difference of immune cells and MSC lineage cells between HO model mice and normal mice. Specifically, immune cells exhibit a hyperinflammatory and hyperproliferative phenotype and MSC lineage cells occupied osteochondral capacity in HO model mice after tibial muscle injury (Figure S13, Supporting Information). Overall, this analysis revealed that the percentage of MSCs lineage cells (i.e., cellular progenitors of HO) were increased after injury, as were the proportions of immune cells in injured tibial samples compared to matched uninjured samples (Figure S14A,B, Supporting Information), suggesting a possible relationship between immune and mesenchymal cell populations following muscle injury in BMP4‐dependent HO mice.

### 2.2 Quiescent MSCs Transition into Cycling MSCs before Differentiating into Osteochondral‐Specific Lineages After Injury

**Figure 2:**
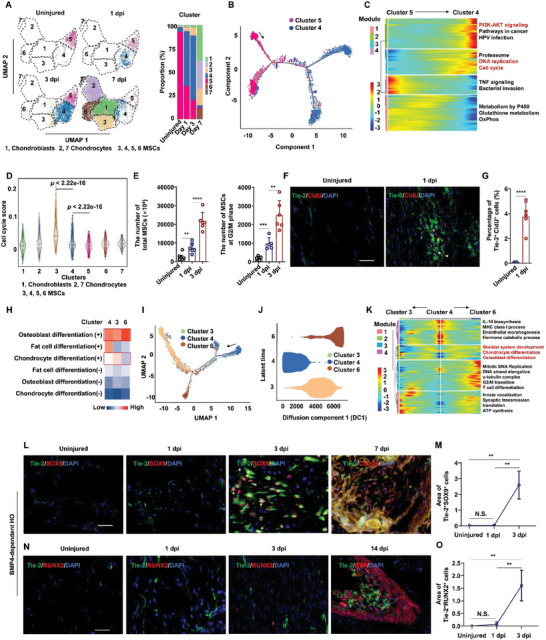
Quiescent MSC enters the cycling stage and differentiates into two lineage cells with osteogenic and chondrogenic capacity after tibial muscle injury. A) UMAP visualization and percentage of MSC linage cells in uninjured and injured tibial muscle of *Nse‐Bmp4* mice at different time points. B) Pseudotemporal trajectories of the quiescent MSCs (cluster 5 mesenchymal lineage cells) and cycling MSCs (cluster 4 mesenchymal lineage cells) in injured tibial muscle of *Nse‐Bmp4* mice using Monocle 2 analysis. C) Heatmap showing relative expressions of four modules genes in quiescent and cycling MSCs along inferred trajectories. D) AddModuleScore analysis of the cell cycle of each type of mesenchymal lineage cells. E) Statistical analysis of the number of total MSCs and MSCs at G2/M phase in uninjured and injured tibial muscle at indicated time points post injury. (*n* = 5 per group). Data are presented as mean ± SD of biological replicates. ** *p* < 0.01, *** *p* < 0.001, **** *p* < 0.0001. F,G) Representative immunofluorescence staining images of F) CldU and G) statistical analysis of Tie2^+^/CldU^+^ cells in uninjured and injured tibial muscle at 1 dpi from *Tie2‐Cre*; *Rosa^mTmG^*; *Nse‐Bmp4* mice. (*n* = 5 per group). Data are presented as mean ± SD of biological replicates. **** *p* < 0.0001. Scale bar, 200 µm. H) GSVA analysis of the capacity of trilineage differentiation of cycling MSCs (cluster 4), cMSC (cluster 3) and oMSC (cluster 6). I) Pseudotemporal trajectories of the cycling MSCs (cluster 4), cMSCs (cluster 3) and oMSCs (cluster 6) in injured tibial muscle of *Nse‐Bmp4* mice using Monocle 2 analysis. J) Pseudotime trajectory analysis of cycling MSC, cMSC and oMSC using diffusion map. K) Heatmap showing relative expressions of four modules genes in cycling MSCs, cMSCs and oMSCs along inferred trajectories. L,M) Representative immunofluorescence staining images of L) Tie2 and SOX9 and statistical analysis of M) Tie2^+^/SOX9^+^ cells in uninjured and injured tibial muscle from *Nse‐Bmp4* mice. (*n* = 5 per group). Data are presented as mean ± SD of biological replicates. ** *p* < 0.01, N. S. indicated no significance. Scale bar, 200 µm. N,O) Representative immunofluorescence staining images of N) Tie2 and RUNX2 and O) statistical analysis of Tie2^+^/RUNX2^+^ cells in uninjured and injured tibial muscle from *Nse‐Bmp4* mice. (*n* = 5 per group). Data are presented as mean ± SD of biological replicates. ** *p* < 0.01, N. S. indicated no significance. Scale bar, 200 µm.

We subsequently analyzed changes in the MSC populations over a 7 d post‐injury observation period and found that MSCs in cluster 5 were principally present in uninjured tibial muscle, whereas MSCs in cluster 4, rather than cluster 5, were the predominant MSC population 1 dpi in the HO lesion (**Figure** **2**A). Since quiescent adult stem cells act as a reservoir for normal tissue turnover and can be activated to enter the cell cycle in response to acute injury or other stimuli,^[^ ^30^ ^]^ we therefore hypothesized that cluster 5 included quiescent MSCs that could give rise to cluster 4 (i.e., MSCs that entered the cell cycle at 1 dpi). Pseudotime trajectory analysis with Monocle 2 indeed supported that cluster 5 likely gave rise to cluster 4 (Figure 2B). Further transcriptomic analysis along the MSC developmental trajectory identified 5306 differentially expressed genes (DEGs) at 1 dpi, which could be categorized into four distinct transcriptional programs (M1–M4) (Figure 2C and Table S1, Supporting Information). Functional enrichment analysis showed that genes associated with “PI3K‐AKT signaling,” “DNA replication,” and “cell cycle” were upregulated in cluster 4 MSCs, suggesting a proliferative state compared to cluster 5 MSCs (Figure 2C).

Additional analysis by AddModuleScore indicated that cell cycle scores, especially of G2/M scores, were higher in cluster 4 than cluster 5 (Figure 2D and Figure S15A, Supporting Information). Cell cycle analysis with the Scran package also suggested that cluster 4 (which emerged at 1 dpi in injured samples) had a higher proportion of MSCs in the S and G2/M phases than cluster 5 (Figure S15B, Supporting Information). To validate that MSCs in uninjured muscle transitioned into the cell cycle at 1 dpi, we used flow cytometry with propidium iodide (PI) staining to quantify actively cycling MSCs at 1 dpi. This assay confirmed that CD45^−^/CD31^−^/PDGFRa^+^/Sca1^+^ MSC numbers were indeed significantly higher at 1 dpi than in uninjured muscle (Figure 2E and Figure S16A, Supporting Information). Moreover, significantly more MSCs were detected in the G2/M phase in 1 dpi samples than in uninjured muscle (Figure 2E and Figure S16B–D, Supporting Information). Furthermore, we sought to validate the switch from quiescent to cycling MSCs by labeling *Tie2* ^+^ MSCs^[^ ^18^, ^24^, ^31^, ^32^, ^33^ ^]^ through a single dose of 5‐chloro‐2′‐deoxyuridine (CldU) in *Tie2‐Cre*; *Rosa^mTmG^*; *Nse‐Bmp4* mice before CTX‐induced muscle injury. At 1 dpi, the proportion of *Tie2* ^+^ MSCs in the cycling state was obviously greater in injured muscle than uninjured muscle (Figure 2F,G). These data confirmed that quiescent MSCs in soft tissues transition into actively cycling MSCs after injury.

We next analyzed MSC development in muscle samples of *Nse‐Bmp4* mice at 3 dpi. We found that cluster 4 MSCs (i.e., cycling MSCs) gradually increased from 1 to 3 dpi, as did MSCs in clusters 3 and 6 (Figure 2A). Based on previous studies that showed MSCs display aberrant repair functions in response to inflammation, differentiating into osteochondral lineage cells,^[^ ^24^, ^32^ ^]^ we speculated that cycling MSCs might further convert into chondrogenic‐ and/or osteogenic‐committed progenitor MSCs by 3 dpi. Gene set variation analysis (GSVA) revealed that cluster 3 MSCs indeed expressed chondrogenesis‐related genes at higher levels but had lower expression of osteogenesis‐ and adipogenesis‐related genes compared to cycling MSCs in cluster 4. In addition, cluster 6 MSCs also expressed osteoblast differentiation‐associated genes at higher levels than cycling MSCs, but appeared to lose their chondrogenic and adipogenic capacity (Figure 2H). These data implied that cluster 3 MSCs entered an early stage of chondrogenic lineage differentiation (cMSC), whereas cluster 6 MSCs showed early capacity for generating osteocyte lineage cells (oMSC). Additional analysis with Monocle 2 and Diffusion Mapping to evaluate the developmental trajectory of cycling MSCs, cMSCs, and oMSCs confirmed that cycling MSCs of cluster 4 were the likely founder population that developed into cluster 3 cMSCs and cluster 6 oMSCs (Figure 2I,J). Functional enrichment analysis showed that genes associated with “skeletal system development”, “chondrocyte differentiation” and “osteoblast differentiation” were enriched in cMSCs and oMSCs compared to cycling MSCs (Figure 2K and Tables S2‐S6, Supporting Information).

To verify that cycling MSCs preferentially differentiate into osteochondral lineage cells in muscle, we again injured the tibial muscle of *Tie2‐Cre*; *Rosa^mTmG^*; *Nse‐Bmp4* mice at 1 and 3 dpi and performed immunostaining to examine the expression of SOX9 and RUNX2. This experiment showed that Tie2^+^ MSCs expressed SOX9 and RUNX2 at 3 dpi, but not at 1 dpi, nor in uninjured muscle (Figure 2L‐O). Consistently, *Prrx1*‐expressing MSCs also expressed SOX9 and RUNX2 at 3 dpi (Figure S17A‐D, Supporting Information). Additionally, Monocle 2 and RNA velocity analyses revealed that cMSCs served as the original cellular source of chondroblasts and mature chondrocytes, supporting their identification as cMSCs (Figure S18A,B, Supporting Information). We also noted that Tie2^+^ MSCs expressed mature chondrocyte marker, COL2a, at 7 dpi and osteocyte marker, BSP, at 14 dpi (Figure 2L,N). Taken together, these results further supported the likelihood that quiescent MSCs in BMP4‐dependent HO mice transition into cycling state MSCs prior to specializing into two distinct lineages that both undergo heterotopic osteochondral ossification.

### 2.3 Prevalent Communication among Immune Cells and Transitioning MSCs within Inflammatory Lesions

**Figure 3:**
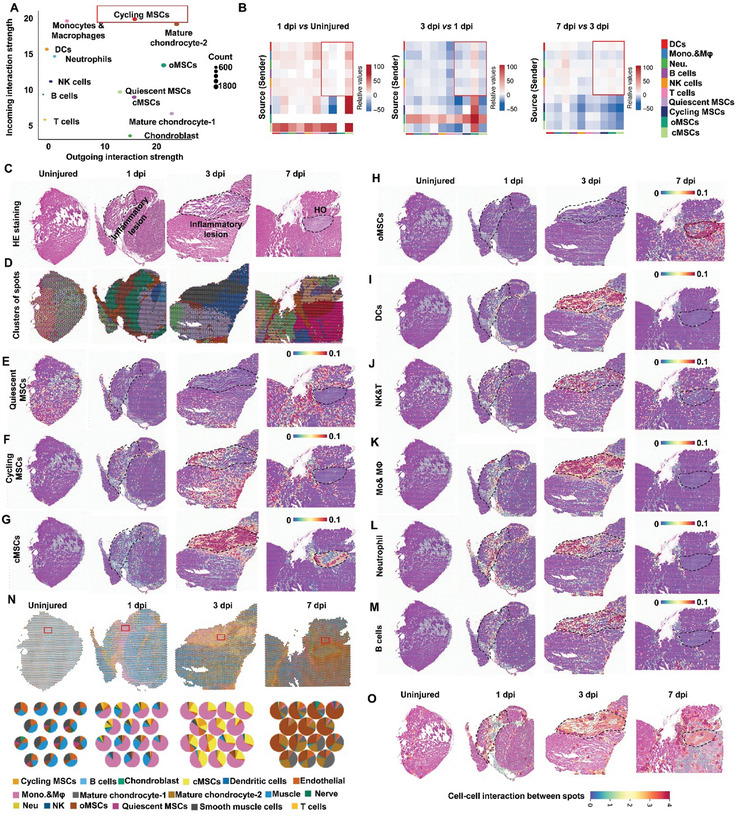
Interaction of MSC with immune cells in the inflammatory niche of injured tibial muscle of *Nse‐Bmp4* mice. A) Signaling role of MSCs and immune cells in tibial muscle of *Nse‐Bmp4* mice covering 4 time points. B) Heatmap displaying differential interactions between immune cells and MSCs at three indicated time points: Day 1 versus Uninjured, Day 3 versus Day 1, and Day 7 versus Day 3. C) HE staining images of uninjured and injured tibial muscles for spatial transcriptome sequencing. D) Mapping of 12 types of cluster cells across tissue regions. E–M) Using AddModule analysis for mapping the cell types generated from single‐cell RNA sequencing to spatial transcriptomics, including E) quiescent MSCs, F) cycling MSCs, G) cMSCs, H) oMSCs, I) DCs, J) NK&T cells, K) macrophages & monocytes, L) neutrophils and M) B cells. N) RCTD analysis of the distribution of each type of cells across the tissue regions at different time points. O) Stlearn analysis of the interaction between two spots across the tissue regions at different time points.

The coinciding timeline of immune cell accumulation and MSC development inspired us to check for possible interactions between immune cells and MSCs (Figure S14A,B, Supporting Information). CellChatDB^[^ ^34^ ^]^ inference of intercellular communication networks among immune cells and MSCs identified at 1, 3, and 7 dpi, as well as in uninjured tissues, revealed several interaction networks with varying interaction strength between MSCs and immune cells (**Figure** **3**A). Of note, among several types of MSCs, cycling MSCs showed the highest outgoing and incoming interaction strength, suggesting a higher capacity for cellular communication than other MSC types in response to inflammation (Figure 3A). Although MSCs exhibited immunoregulative properties, which is important for tissue regeneration, we mainly focused on the influence of immune cells on MSCs, owing to their substantial contribution to HO formation.^[^ ^23^, ^35^ ^]^ We found that the interaction number at 1 dpi was increased between monocyte/macrophage, neutrophil, and NK immune cells with quiescent and cycling MSCs, but not between MSCs and DC or T/B cells, compared to that in uninjured tissue (Figure 3B and Table S7, Supporting Information). Interaction strength analysis further confirmed this dynamic (Table S8, Supporting Information). Moreover, since cycling MSCs could give rise to cMSCs and oMSCs at 3 dpi, we further compared interactions among immune cells and MSCs between 3 dpi and the uninjured group, as well as those at 1 dpi. We found that signaling from immune cells to cMSCs at 3 dpi was increased compared to the uninjured group but decreased compared to that in 1 dpi samples. By contrast, signaling from immune cells to oMSCs was enhanced at 3 dpi over that at 1 dpi, and further increased by 7 dpi (Figure 3C and Tables S7 and S8, Supporting Information).

After characterizing cell‐to‐cell interactions from immune cells to MSCs, we next examined the spatial distribution of these communicating cell populations within the inflammatory niche of injured muscles. To this end, we again injured the tibial muscle of *Nse‐Bmp4* mice and collected samples of injured tissue at 1, 3 and 7 dpi, as well as uninjured samples for spatial transcriptome analysis^[^ ^36^ ^]^ (Figure 3C and Figure S19A, Supporting Information). Subsequent UMAP dimensionality reduction and unsupervised clustering of the spatial transcriptomics data identified 12 clusters across the 3 time points and uninjured controls (Figure 3D and Figure S19B, Supporting Information). Specific sites within sections of the inflammatory lesion (termed spots, hereafter) that we histologically outlined were annotated as containing clusters 6, 7, and 8, and had detectable expression of both immune cell and MSC markers (Figure S19C and Table S9, Supporting Information). AddModuleScore analysis using gene signatures derived from our scRNA‐seq data to map MSCs and immune cells showed that quiescent MSCs were mainly distributed in the uninjured muscle (Figure 3E), while cycling MSCs initially accumulated in the lesion at 1 dpi and were further enriched at 3 dpi (Figure 3F). Similarly, cMSCs and oMSCs were mainly detected in the inflammatory lesion at 3 dpi (Figure 3G,H). Additionally, several immune cells, including monocytes/macrophages, neutrophils, DCs, NK&T and B cells were all present in the inflammatory lesion at 1 and 3 dpi (Figure 3I–M).

We next examined the potential interplay between MSCs and immune cells within spots during the post‐injury inflammatory stage (1 and 3 dpi). For this analysis, we performed robust cell type decomposition (RCTD) analysis for injured muscle at 1 and 3 dpi. We found that each immune cell type could be present at the same time as cycling MSCs, but not quiescent MSCs, within any given spot at 1 dpi (Figure 3N and Table S10, Supporting Information). Moreover, the number of spots containing monocytes/macrophages along with cycling MSCs was the highest, suggesting these cells shared the strongest communications (Figure 3N and Table S10, Supporting Information). At 3 dpi, T cells, B cells, NK cells and neutrophils were rarely found sharing spots with cycling MSCs, cMSCs and oMSCs, whereas monocytes/macrophages and DCs were still abundant in the presence of these MSCs (Figure 3N and Table S10, Supporting Information). In addition, stlearn analysis exploring potential spot–spot communication between immune cells and MSCs revealed that spot–spot communication was greater within inflammatory lesions at 1 and 3 dpi than in uninjured tissue (Figure 3O). These analyses collectively suggest that immune cells, especially monocytes/macrophages, are located in close proximity and interact with cycling MSCs, oMSCs, and cMSCs in the inflammatory niche of injured muscle tissue of BMP4‐dependent HO mice.

### 2.4 Macrophages Promote MSC Proliferation and Osteochondral Differentiation in Injured Muscle of HO Model Mice

**Figure 4:**
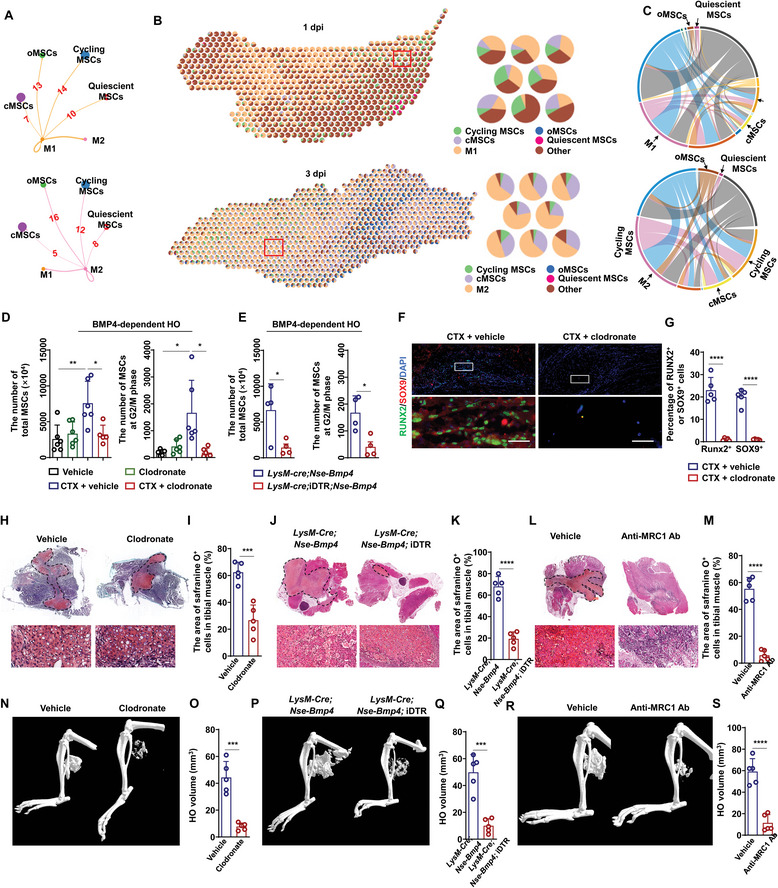
Macrophages promote transition of quiescent MSCs into cycling MSCs and aberrant osteochondral differentiation of cycling MSCs. A) The interaction number for M1 and M2 macrophages (source) and each subtype of MSCs (recipients). B) RCTD analysis for the interaction among M1 macrophages and MSCs at 1 dpi and M2 macrophages and MSCs at 3 dpi. C) Interaction analysis for M1 macrophages and MSCs at 1 dpi and M2 macrophages and MSCs at 3 dpi. D) Statistical analysis of total MSCs and MSCs at G2/M phase in injured tibial muscle of *Nse‐Bmp4* mice with vehicle or clodronate liposome treatment at 1 dpi. (*n* = 5 per group). Data are presented as mean ± SD of biological replicates. * *p* < 0.05, ** *p* < 0.01. E) Statistical analysis of the number of total MSCs and MSCs at G2/M phase in injured tibial muscle of *LysM‐Cre*; *Nse‐Bmp4* and *LysM‐Cre*; *iDTR*; *Nse‐Bmp4* mice with DT treatment at 1 dpi. (*n* = 4 per group). Data are presented as mean ± SD of biological replicates. * *p* < 0.05. F) Representative immunofluorescence staining images and G) statistical analysis of the area of RUNX2^+^ and SOX9^+^ in the injured tibial muscle of *Nse‐Bmp4* mice with vehicle or clodronate treatment. (*n* = 5 per group). Data are presented as mean ± SD of biological replicates. **** *p* < 0.0001. Scale bar, 50 µm. H) Representative images and I) statistical analysis of safranine O^+^ area in injured tibial muscle of *Nse‐Bmp4* mice with vehicle or clodronate liposome treatment. (*n* = 5 per group). Data are presented as mean ± SD of biological replicates. *** *p* < 0.001. Scale bar, 50 µm. J) Representative images and K) statistical analysis of safranine O^+^ area in injured tibial muscle of *LysM‐Cre*; *Nse‐Bmp4 and LysM‐Cre; iDTR; Nse‐Bmp4* mice with DT treatment. (*n* = 5 per group). Data are presented as mean ± SD of biological replicates. **** *p* < 0.0001. Scale bar, 50 µm. L) Representative images and M) statistical analysis of safranine O^+^ area in injured muscle of *Nse‐Bmp4* mice with vehicle or anti‐MRC1 antibody treatment. (*n* = 5 per group). Data are presented as mean ± SD of biological replicates. **** *p* < 0.0001. Scale bar, 50 µm. N) Representative microCT images and O) statistical analysis of HO volume in injured tibial muscle of *Nse‐Bmp4* mice with vehicle or clodronate treatment from 7 to 14 dpi. (*n* = 5 per group). Data are presented as mean ± SD of biological replicates. * *p* < 0.05, ** *p* < 0.01, **** *p* < 0.0001, N. S. indicated no significance. P) Representative microCT images and Q) statistical analysis of HO volume in injured tibial muscle of *LysM‐Cre*; *Nse‐Bmp4 and LysM‐Cre; iDTR; Nse‐Bmp4* mice with DT treatment. (*n* = 5 per group). Data are presented as mean ± SD of biological replicates. *** *p* < 0.001. R) Representative microCT images and S) statistical analysis of HO volume in injured tibial muscle of *Nse‐Bmp4* mice with either vehicle or anti‐MRC1 antibody treatment (*n* = 5 per group). Data are presented as mean ± SD of biological replicates. **** *p* < 0.0001.

Monocyte‐derived macrophages, including M1 and M2 subtypes, have been shown to play a role in wound healing, including HO.^[^ ^37^ ^]^ However, the role of macrophage dynamics in MSC fate determination after soft tissue injury remains poorly understood. We first examined interactions between M1 macrophages and each type of MSC at each time point and in control tissue via CellChatDB analysis. We found that the number of interactions between M1 macrophages and quiescent MSCs at 1 dpi was increased compared to their interactions in uninjured tissue (Table S11, Supporting Information). Moreover, M1 macrophages had more interactions with cycling MSCs than any other pairwise interactions between immune cell‐MSC subtypes (**Figure** **4**A and Table S11, Supporting Information). Further RCTD analysis examining the distribution of M1 macrophages and MSCs within spots in spatial transcriptome data showed that M1 macrophages mainly shared spots with cycling MSC, rather than quiescent MSCs, cMSCs or oMSCs, suggesting that M1 macrophages could potentially promote transition from quiescent to cycling MSCs and subsequent differentiation (Figure 4B,C).

We thus hypothesized that M1 macrophages might regulate the proliferation and/or differentiation of MSCs in muscle. Therefore, to investigate the effect of M1 macrophages on cycling MSCs, we pre‐treated *Nse‐Bmp4* mice with clodronate liposome via intravenous injection to deplete circulatory monocytes before tibial muscle injury, preventing infiltration of monocyte‐derived M1 macrophages to the injury site (Figure S20A‐E, Supporting Information). Following 3 treatments of clodronate or vehicle liposome, we injured the tibial muscle of *Nse‐Bmp4* mice and examined the number of MSCs at 1 dpi. Flow cytometry analysis indicated that the number of total MSCs and MSCs at G2/M phase in injured muscle of clodronate‐treated *Nse‐Bmp4* mice were significantly lower than in injured mice treated with the vehicle (Figure 4D). Additionally, we used *LysM‐Cre*; iDTR; *Nse‐Bmp4* mice, in which monocytes/macrophages were depleted by pre‐injection of diptheria toxin (DT), to further determine how M1 macrophages could affect MSC proliferation at 1 dpi. As expected, the number of total MSCs and G2/M phase MSCs were significantly decreased compared to that in *LysM‐Cre*; *Nse‐Bmp4* control mice (Figure 4E). We next explored the effects of M1 macrophages on cycling MSC differentiation in muscle. Immunostaining of SOX9 and RUNX2 revealed that the percentages of SOX9^+^ or RUNX2^+^ cells in the injury site of clodronate‐treated *Nse‐Bmp4* mice were significantly lower than their proportions in the vehicle group (Figure 4F,G). These results indicated that pro‐inflammatory (M1) macrophages promote transition of quiescent MSCs into cycling MSCs and further differentiation of cycling MSCs into cMSCs and oMSCs.

In light of these apparent effects of M1 macrophages on cycling MSCs in injured muscle at 1 dpi, we next checked the function of M2 macrophages at 3 dpi. CellChatDB analysis revealed that M2 macrophages most likely regulate the function of cycling MSCs, cMSCs and oMSCs (Figure 4A and Table S12, Supporting Information). To spatially assess the function of M2 macrophages, we performed RTCD analysis for macrophages at 3 dpi. The results showed that M2 macrophages could share spots with cMSCs and oMSCs, but not cycling MSCs, indicating that M2 macrophages potentially regulate the osteochondral differentiation of MSCs (Figure 4B,C). We next injected clodronate or liposome vehicle into injured muscle tissue of *Nse‐Bmp4* mice from 3 to 7 dpi and found that chondrocyte differentiation was limited in mice with macrophage depletion (Figure 4H,I and Figure S21A,B, Supporting Information). Additionally, significantly fewer COL2^+^ or Safranine O^+^ chondrocytes were detected in *LysM‐Cre*; *iDTR*; *Nse‐Bmp4* mice compared to that in *LysM‐Cre*; *Nse‐Bmp4* mice (Figure 4J,K and Figure S21C,D, Supporting Information). Moreover, since M2 macrophages specifically expressed *Mrc1*, we administered an MRC1 neutralizing antibody (Abs) via intravenous injection in *Nse‐Bmp4* mice from 3 to 7 dpi. We observed that Safranine O^+^ chondrocytes cells were significantly decreased in MRC1 Abs‐treated *Nse‐Bmp4* mice compared to that in the vehicle group (Figure S21E,F, Supporting Information and Figure 4L,M).

We subsequently investigated the role of M2 macrophages in heterotopic osteogenesis of oMSCs. Spatial transcriptome analysis revealed that *Mrc1* ^+^ M2 macrophages were the dominant immune cells around peri‐chondrocyte regions, which were also enriched for oMSCs (Figure S22A,B, Supporting Information). RCTD analysis further demonstrated that *Mrc1* ^+^ M2 macrophages shared spots with oMSCs (Figure S22C, Supporting Information). Moreover, clodronate‐ and *LysM‐Cre*; iDTR‐mediated macrophage depletion from 7 to 14 dpi in injured muscle of *Nse‐Bmp4* mice indeed alleviated ectopic bone formation, indicated by the significantly lower HO volume in these mice compared to the vehicle group (Figure 4N–Q). Consistent with these findings, HO volume was significantly decreased in MRC1 Abs‐treated *Nse‐Bmp4* mice compared to the vehicle group (Figure 4R,S). Taken together, these results suggested that macrophages play a substantial role in BMP4‐dependent HO formation via promoting MSC cycling and differentiation.

### 2.5 Neutrophils and NK Cells Promote MSC Proliferation

**Figure 5:**
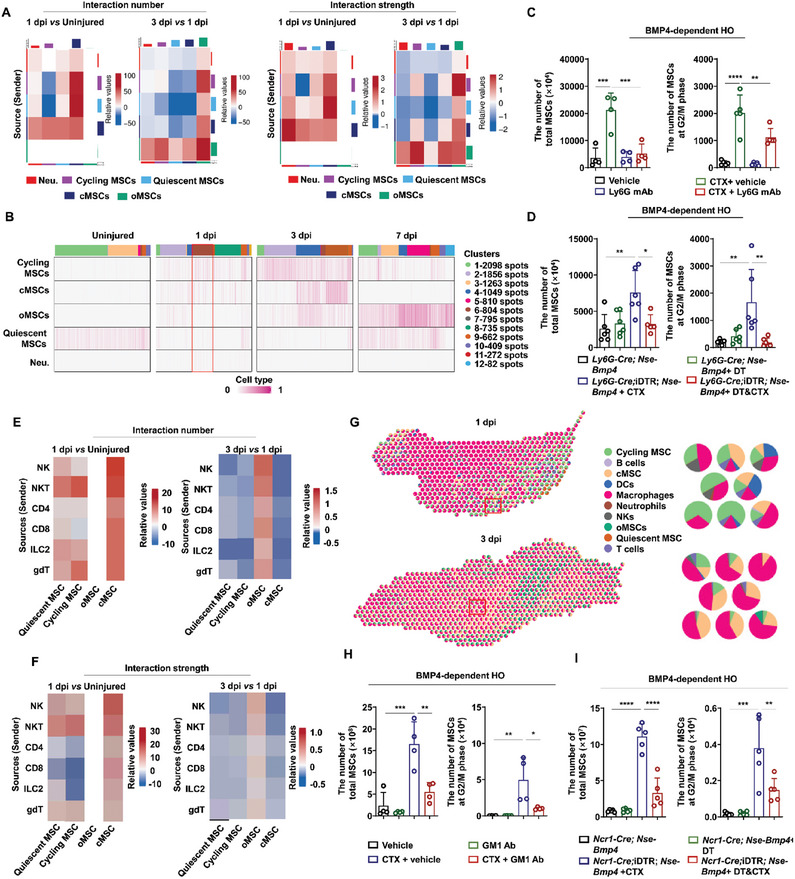
Neutrophils and NK cells potentiates the proliferation of MSC in injured tibial muscle of *Nse‐Bmp4* mice. A) The differential interaction number and strength between neutrophils and MSCs at 1 dpi compared to uninjured group, or at 3 dpi compared to 1 dpi. B) RCTD analysis of the interaction between neutrophils and each subtype of MSCs. C) Statistical analysis of the number of total MSCs and MSCs at G2/M phase in injured tibial muscle of *Nse‐Bmp4* mice with either vehicle or Ly6G antibody treatment at 1 dpi (*n* = 4 per group). Data are presented as mean ± SD of biological replicates. ** *p* < 0.01, *** *p* < 0.001, **** *p* < 0.0001. D) Statistical analysis of number of total MSCs and MSCs at G2/M phase in injured tibial muscle of *Ly6G‐Cre*; *Nse‐Bmp4 and Ly6G‐Cre; iDTR; Nse‐Bmp4* mice with DT treatment. (*n* = 6 per group). Data are presented as mean ± SD of biological replicates. * *p* < 0.05, ** *p* < 0.01. E,F) The differential interaction number and strength of NK or T cells with MSCs at 1 dpi compared to uninjured group, or at 3 dpi compared to 1 dpi. G) RCTD analysis of the interaction of NK or T cells with each subtype of MSCs. H) Statistical analysis of the number of total MSCs and MSCs at G2/M phase in injured tibial muscle of *Nse‐Bmp4* mice with either vehicle or GM1 antibody treatment at indicated time points after injury. (*n* = 4 per group). Data are presented as mean ± SD of biological replicates. * *p* < 0.05, ** *p* < 0.01, *** *p* < 0.0001. I) Statistical analysis of number of total MSCs and MSCs at G2/M phase in injured tibial muscle of *Ncr1‐Cre*; *Nse‐Bmp4 and Ncr1‐Cre; iDTR; Nse‐Bmp4* mice with DT treatment. (*n* = 5 per group). Data are presented as mean ± SD of biological replicates. ** p < 0.01, *** p < 0.001, **** *p* < 0.0001.

We next examined whether and how other immune cells, in addition to macrophages, might affect the transition of cycling MSCs (i.e., MSC proliferation). Investigation of neutrophils, which are also rapidly recruited to injury sites to eliminate pathogens and facilitate tissue repair,^[^ ^38^, ^39^ ^]^ confirmed that neutrophils infiltrated the inflammatory lesion by 1 dpi in *Nse‐Bmp4* mice, remained at stable levels through 3 dpi, then progressively decreased (Figure 1I and Figure S23A–D, Supporting Information), which implied a possible function in MSC fate determination. CellChatDB analysis of neutrophil interactions with each MSC type across different timepoints and uninjured controls showed that neutrophils had more potential signaling interactions with cycling MSCs than other MSC types (**Figure** **5**A, Tables S7 and S8, Supporting Information). Similarly, RCTD analysis indicated that neutrophils shared spots (in cluster 6) with cycling MSCs more than other MSCs (Figure S19B, Supporting Information and Figures 2B and 5B), together suggesting that neutrophils could influence cycling MSCs.

To further examine the effect of neutrophils on cycling MSCs, we pre‐treated *Nse‐Bmp4* mice with Ly6G monoclonal antibody (mAb) via intravenous injection to deplete the neutrophils prior to tibial muscle injury, thus preventing neutrophil infiltration in the injury site (Figure S24A‐D, Supporting Information). Flow cytometry quantification of MSCs at 1 dpi in injured tibial muscle of *Nse‐Bmp4* mice pre‐treated with 3 doses of Ly6G mAb or PBS indicated that significantly fewer total MSCs and MSCs at G2/M phase were present at the inflammatory lesion in mice with neutrophil depletion compared to the vehicle control group (Figure 5C). Additionally, the number of total MSC and MSC at G2/M phase were significantly decreased in *Ly6G‐Cre*; iDTR; *Nse‐Bmp4* mice compared to *Ly6G‐Cre*; *Nse‐Bmp4* mice (Figure 5D).

We found that interaction number and interaction strength between NKs and quiescent MSCs also increased at 1 dpi (Figure 5E,F) and RCTD analysis confirmed that NK cells shared spots primarily with cycling MSCs (Figure 5G). To better understand how their interactions affected cycling MSCs, we depleted NK cells from the inflammatory lesion by intravenously injecting *Nse‐Bmp4* mice with Asialo GM1 antibody^[^ ^40^ ^]^ (NK cell‐depleting antibody) before tibial muscle injury, thus preventing infiltration of NK cell, but not macrophages (Figure S24E–H, Supporting Information). After 3 injections of Asialo GM1 antibody or PBS, tibial muscle was injured and MSCs were counted by flow cytometry, which revealed that the abundance of total MSCs and MSCs at G2/M phase was lower in injured tibial muscle of NK‐depleted mice than in the injury site of PBS‐treated control mice at 1 dpi (Figure 5H). Additionally, *Ncr1‐Cre*; iDTR; *Nse‐Bmp4* mice, in which NK cells were depleted by pre‐injection of diptheria toxin (DT), showed significantly decreased numbers of both total and G2/M phase MSCs compared with *Ncr1‐Cre*; *Nse‐Bmp4* mice (Figure 5I). These results indicated that neutrophils and NK cells also promote MSC cycling and proliferation.

### 2.6 CD4^+^ and CD8^+^ T Cells Potentiate Chondrogenic Differentiation by MSCs in Muscle After Injury

We next investigated the effect of immune cells on MSC differentiation in muscle during HO formation. To this end, we measured interaction number and strength between these T cells and quiescent or cycling MSCs at 1 dpi. Compared to their interactions in uninjured muscle samples, we found no obvious differences in CD4^+^ or CD8^+^ T cell interaction numbers or interaction strength with either quiescent or cycling MSCs at 1 dpi (Figure 5E,F). RCTD analysis revealed that CD4^+^ and CD8^+^ T cells rarely shared the same spot with cycling MSCs or quiescent MSCs (Table S13, Supporting Information). Conversely, NKT cells, like NK cells, showed enhanced interaction number and strength with quiescent or cycling MSCs at 1 dpi, compared to that in uninjured tibial muscle (Figure 5E,F). Additionally, the number of gdT cell interactions with quiescent or cycling MSC increased after injury, but the strength of these interactions with quiescent/cycling MSCs was unchanged at 1 dpi compared to that in uninjured tibial muscle. These results suggested that gdT cells did not likely exert regulatory effects on MSC proliferation.

**Figure 6:**
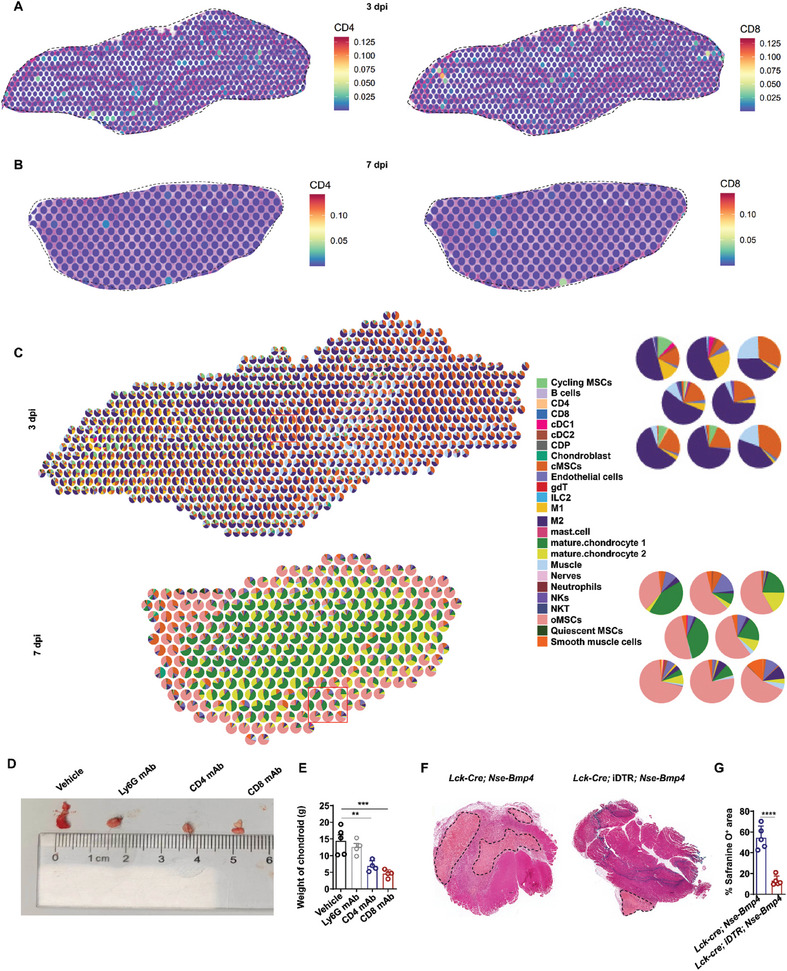
CD4^+^ and/or CD8^+^ T cells promote chondrogenic differentiation of cMSCs. A,B) AddModuleScore analysis of A) *Cd4* and *Cd8* at 3 and B) 7 dpi using spatial transcriptome analysis. C) RCTD analysis of CD4^+^ and CD8^+^ T cells at 3 and 7 dpi using spatial transcriptome analysis. D) Representative images and E) statistical analysis of the chondroid weight after either Ly6G mAb, CD4 mAb or CD8 mAb treatment. (*n* = 4 or 5 per group). Data are presented as means ± SD of biological replicates. ** *p* < 0.01, *** *p* < 0.001. F) Representative safranine O staining images and G) statistical analysis of the injured tibial muscle of *Lck*‐*Cre*; *Nse*‐*Bmp4* mice and *Lck‐Cre*; iDTbR; *Nse*‐*Bmp4* mice (*n* = 5 per group). Data are presented as means ± SD of biological replicates. **** *p* < 0.0001. Scale bar, 200 µm.

Since CD4^+^ and CD8^+^ T cells account for largest population of immune cells that function in adaptive immunity, we next focused on their possible role in osteochondral differentiation of MSCs. CellchatDB analysis revealed that CD4^+^ and CD8^+^ T cells showed more and stronger interactions with cMSCs and oMSCs at 3 dpi compared to that in other samples (Figure 5E and Table S13, Supporting Information). RCTD analysis indicated that CD4^+^ and CD8^+^ T cells shared spots with cMSCs and cycling MSCs, suggesting that these immune cells could potentially regulate osteochondral differentiation of MSCs (**Figure** **6**A–C). Because chondrocyte differentiation was found to occur from 3 to 7 dpi, preceding osteocyte formation, we injected CD4 mAb or CD8 mAb into the injured muscle of *Nse‐Bmp4* mice from 3 to 7 dpi (for 3 injections) to deplete these cell types individually. We found that chondroid weights decreased following CD4 mAb treatment (Figure 6D). Likewise, chondrogenic differentiation of MSCs was also significantly inhibited in mice treated with CD8 mAb (Figure 6D,E). Additionally, *Lck‐Cre*‐mediated T cell depletion in iDTR; *Nse‐Bmp4* mice after DT administration from 3 to 7 dpi resulted in a significant decrease in Safranine O^+^ cells in *Lck‐Cre*; *iDTR*; *Nse‐Bmp4* mice compared to that in *Lck‐Cre*; *Nse‐Bmp4* mice (Figure 6F,G). RCTD analysis of the effects of CD4^+^ and CD8^+^ T cells on osteogenic differentiation of MSCs showed that no CD4^+^ and CD8^+^ T cells shared the same spot with oMSCs, indicating that neither T cell subtype affected osteogenic differentiation of MSCs (Figure 6C). These results collectively indicated that CD4^+^ and CD8^+^ T cells could promote chondrogenic, but not osteogenic differentiation of MSCs following muscle injury of *Nse*‐*Bmp4* mice.

### 2.7 Inflammatory Immune Cells Promote Tendon MSC Development into HO

**Figure 7:**
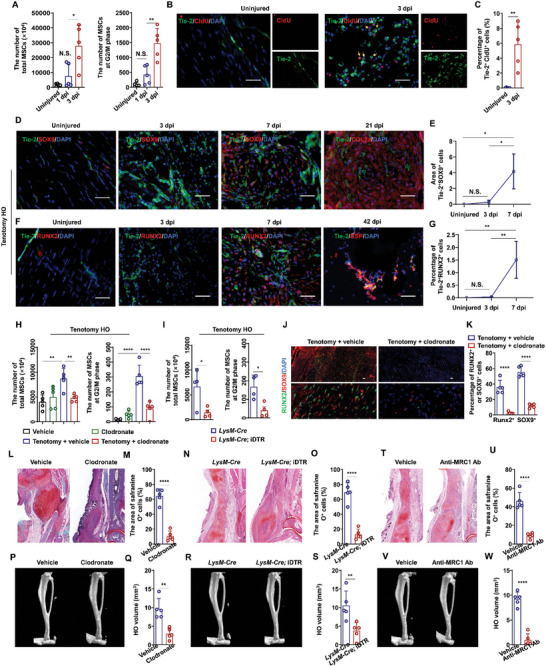
Macrophages promotes MSC proliferation and osteochondral differentiation in tendon. A) Statistical analysis of the number of total MSCs and MSCs at G2/M phase in injured tendon at indicated time points after injury. (*n* = 5 per group). Data are presented as mean ± SD of biological replicates. * *p* < 0.05, ** *p* < 0.01, N. S. indicated no significance. Representative immunofluorescence staining images of B) Tie2 and CldU and C) statistical analysis of Tie2^+^/CldU^+^ cells in uninjured and injured tendon at 3 dpi from *Nse‐Bmp4* mice. (*n* = 5 per group). Data are presented as mean ± SD of biological replicates. ** *p* < 0.01. Scale bar, 200 µm. Representative IF staining images of D) Tie2, SOX9, and Col2a and statistical analysis of E) Tie2^+^/SOX9^+^ cells in uninjured and injured tendon from WT mice. (*n* = 5 per group). Data are presented as mean ± SD of biological replicates. * *p* < 0.05, N. S. indicated no significance. Scale bar, 200 µm. Representative IF staining images of F) Tie2, RUNX2 and BSP and G) statistical analysis of Tie2^+^/RUNX2^+^ cells in uninjured and injured tendon from WT mice. (*n* = 5 per group). Data are presented as mean ± SD of biological replicates. ** *p* < 0.01, N. S. indicated no significance (unpaired two‐tailed t‐test). Scale bar, 200 µm. H) Statistical analysis of the number of total MSCs and MSCs at G2/M phase in injured tendon of WT mice with either vehicle or clodronate liposome treatment at 3 dpi. (*n* = 5 per group). Data are presented as mean ± SD of biological replicates. ** *p* < 0.01, **** *p* < 0.0001. I) Statistical analysis of the number of total MSCs and MSCs at G2/M phase in injured tendon of *LysM‐Cre* and *LysM‐Cre*; *iDTR* mice with DT treatment at indicated time points after injury. (*n* = 4 per group). Data are presented as mean ± SD of biological replicates. * *p* < 0.05. Representative IF staining images of J) SOX9 and RUNX2 and K) statistical analysis of SOX9^+^ or RUNX2^+^ cells in injured tendon from WT mice with either vehicle or clodronate treatment. (*n* = 5 per group). Data are presented as mean ± SD of biological replicates. **** *p* < 0.0001. Scale bar, 200 µm. L) Representative images and M) statistical analysis of safranine O^+^ area in injured tendon of WT mice with either vehicle or clodronate treatment. (*n* = 5 per group). Data are presented as mean ± SD of biological replicates. **** *p* < 0.0001. Scale bar, 200 µm. N) Representative images and O) statistical analysis of safranine O^+^ area in injured tendon of *LysM‐Cre and LysM‐Cre; iDTR* mice with DT treatment. (*n* = 5 per group). Data are presented as mean ± SD of biological replicates. **** *p* < 0.0001. Scale bar, 200 µm. P) Representative images and Q) statistical analysis of HO volume in injured tendon of WT mice with either vehicle or clodronate treatment. (*n* = 5 per group). Data are presented as mean ± SD of biological replicates. **** *p* < 0.0001. Scale bar, 200 µm. R) Representative microCT images and S) statistical analysis of HO in injured tendon of *LysM‐Cre* and *LysM‐Cre*; iDTR mice. (*n* = 5 per group). Data are presented as mean ± SD of biological replicates. ** *p* < 0.01. T) Representative safranine O^+^ area and V) microCT images and U) statistical analysis of safranine O^+^ area and W) HO volume in injured tendon of WT mice with MRC1 antibody (*n* = 5 per group). Data are presented as mean ± SD of biological replicates. ** *p* < 0.01 **** *p* < 0.0001.

Since MSCs in tendon may also differentiate into osteochondral cells, resulting in HO, under inflammatory conditions,^[^ ^35^, ^41^, ^42^ ^]^ we next examined whether quiescent MSCs resident in tendon also entered the cell cycle after injury. Flow cytometry analysis using the same markers (CD45^−^/CD31^−^/PDGFRa^+^/Sca1^+^) as above indicated that MSC populations were slightly higher in injured tendon compared to the sham group at 1 dpi, but significantly increased over that in the sham group by 3 dpi, as did the population of MSCs in the G2/M phase at the tenotomy site at 3 dpi compared to that in uninjured tendon (**Figure** **7**A and Figure S25, Supporting Information), suggesting that the timeline of MSC response to inflammation differed between tendon and muscle tissues. Additionally, Tie2^+^ MSCs contribute to each stage of tenotomy HO (Figures S26A–C and S27A–D, Supporting Information). At day 3 post tenotomy in *Tie2‐Cre*; *Rosa^mTmG^* mice, the proportion of *Tie2* ^+^ MSCs in the cycling state in tendon was significantly higher than in uninjured tendon (Figure 7B,C).

Following the same timeline used to observe MSCs in muscle, we next examined the MSC capacity for osteochondral differentiation in tendon at 7 dpi. Immunostaining analysis indicated that Tie2^+^ MSCs did not express SOX9 or RUNX2 in either uninjured tendon or injured tendon at 3 dpi (i.e., the proliferation stage), but showed high expression of SOX9 and RUNX2 at 7 dpi, implying that Tie2^+^ tendon MSCs could give rise to osteochondral lineages after proliferation (Figure 7D–G). At 21 dpi in tendon, Tie2^+^ MSCs expressed the mature chondrocyte marker, COL2a (Figure 7D) and expressed osteocyte marker, BSP, at 42 dpi (Figure 7F).

Additionally, we investigated whether macrophages also participate in fate determination of tendon‐resident cycling MSCs from 3 to 7 dpi via intravenous injection of clodronate liposomes (Figure S28A,B, Supporting Information). Following depletion of macrophages via either clodronate or by genetic depletion (*i.e*., *LysM‐Cre*; iDTR), we found that the total MSC and G2/M phase MSC populations were significantly decreased in injured tendon compared to that in controls (Figure 7H,I).^[^ ^37^ ^]^ Furthermore, the proportions of SOX9^+^ or RUNX2^+^ cells were also significantly lower in the injured tendon of clodronate‐treated WT mice compared to the vehicle group (Figure 7J,K). In addition, the chondrocyte abundance and HO formation were also inhibited following macrophage depletion by chemical or genetic methods (Figure 7L–S), while Safranine O^+^ cell numbers and HO volume were significantly lower in tenotomy mice given intravenous injection of MRC1 neutralizing antibody from 7 to 21 dpi compared to vehicle control mice (Figure 7T–W).

We then explored the effects of other immune cells on tendon MSC fate determination. Consistent with results in muscle, chemically or genetically induced depletion of either neutrophils or NK cells resulted in significantly impaired proliferation of tendon MSCs (Figure S29A–D, Supporting Information). Moreover, inhibition of CD4^+^ or CD8^+^ T cells led to significantly reduced chondrocyte differentiation and lower volume of HO in tendon tissue (Figure S30A–D, Supporting Information). DC and B cells did not affect HO formation (Figure S31A–H, Supporting Information). Taken together, these results supported the likelihood that the inflammatory niche could enhance aberrant proliferation and osteochondral differentiation of tendon MSCs.

### 2.8 Molecular Regulation of MSC by Immune Cells in the Inflammatory Niche

**Figure 8:**
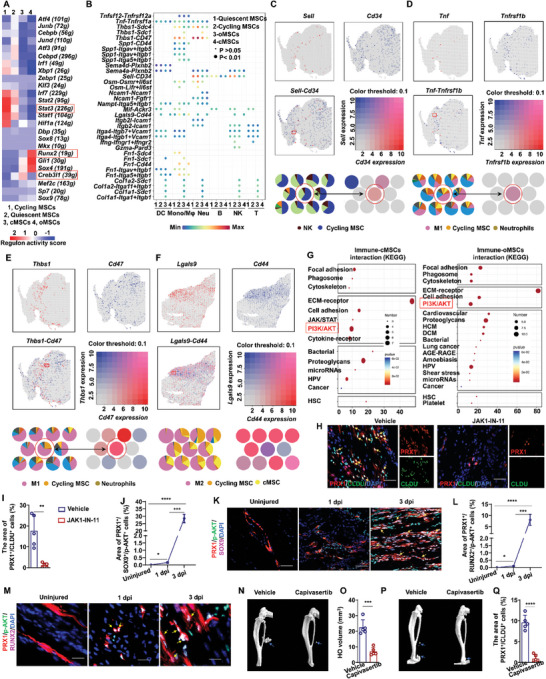
Molecular regulation of MSC by immune cells. A) Heatmap of the activity of the regulons of each subtype of MSCs. B) Molecular interaction pairs between immune cells (donors) and MSCs (recipients). C) Co‐expression pattern of *Sell* and *Cd34* in the spatial section and the spatial feature plots of *Sell* and *Cd34*‐enriched NK/MSC expansion units at 1 dpi. D) Coexpression pattern of *Tnf* and *Tnfrsf1b* in the spatial section and the spatial feature plots of *Tnf* and *Tnfrsf1b*‐enriched NK/MSC expansion units at 1 dpi. E) Co‐expression pattern of *Thbs1* and *Cd47* in the spatial section and the spatial feature plots of *Thbs1* and *Cd47*‐enriched NK/MSC expansion units at 1 dpi. F) Coexpression pattern of *Lgals9* and *Cd44* in the spatial section and the spatial feature plots of *Lgals9* and *Cd44*‐enriched NK/MSC expansion units at 3 dpi. G) KEGG analysis of immune‐MSC interaction pairs. H) IF staining and I) statistical analysis of the PRX1^+^/CldU^+^ MSCs in injured tendon with or without JAK inhibitor (JAK1‐IN‐11) treatment. (*n* = 5 per group). Data are presented as mean ± SD of biological replicates. ** *p* < 0.01. Scale bar, 50 µm. J) IF staining and K) statistical analysis of the PRX1^+^/SOX9^+^/p‐AKT^+^ MSCs in injured tendon at different time points post injury. (*n* = 5 per group). Data are presented as mean ± SD of biological replicates. *** *p* < 0.001, **** *p* < 0.0001, Scale bar, 50 µm. M) IF staining and L) statistical analysis of the PRX1^+^/RUNX2^+^/p‐AKT^+^ MSCs in injured tendon at different time points post injury. (*n* = 5 per group). Data are presented as mean ± SD of biological replicates. *** *p* < 0.001, **** *p* < 0.0001, Scale bar, 20 µm. N) Representative microCT images and O) statistical analysis of muscle HO volume in *Nse‐Bmp4* mice after Capivasertib treatment. (*n* = 5 per group). Data are presented as mean ± SD of biological replicates. *** *p* < 0.001. P) Representative microCT images and Q) statistical analysis of tendon HO volume after Capivasertib treatment. (*n* = 5 per group). Data are presented as mean ± SD of biological replicates. **** *p* < 0.0001.

Based on our results showing that immune cells in the inflammatory niche can regulate MSC fate determination in muscle and tendon, we next sought to identify the molecular networks that potentially control MSC development during HO formation. To this end, we first investigated the transcription factors responsible for MSC proliferation and differentiation. Regulon analysis revealed that *Stat* family genes, including *Stat3*, *Stat2* and *Stat1*, were more actively transcribed in cycling MSCs than in quiescent MSCs, suggesting that the JAK‐STAT signaling pathway contributed to inducing cell cycle entry in cycling MSCs (**Figure** **8**A). Further analysis of potential signals interaction between immune cells and MSCs at 1 dpi by CellChatDB identified 34 significant ligand‐receptor pairs that might contribute to aberrant proliferation in MSCs (Figure 8B). These pairs could be categorized into 17 distinct signaling pathways, including the OSM, TWEAK, TNF, THBS, SPP1, SEMA4, SELL, NCAM, VISFATIN, MIF, GALECTIN, ITGAL‐ITGB2, VCAM, IFN‐II, PARs, FN1 and COLLAGEN pathways.

Since MSCs undergo two pathophysiological processes to form HO, i.e., rapid proliferation followed by osteochondral differentiation, we next sought to identify signal molecules that played key roles in regulating these two processes. Following our above data that confirmed M1 macrophages, neutrophils, and NK cells promote MSC proliferation at 1 dpi, we therefore characterized the suite of immune cell‐derived molecules that might participate in driving proliferation. CellChatDB analysis was used to first identify ligand genes in NKs that could interact with receptors expressed in quiescent and/or cycling MSCs at 1 dpi, since our observation that quiescent MSCs transition to actively cycling MSCs suggested that immune cells initially interact with quiescent MSCs, then consistently interact with cycling MSCs. We found that the expression of eight ligand genes (*Sema4d*, *Sema4a*, *Mif*, *Itgb2*, *Itga4*, *Gzma*, *Sell* and *Pdgfb*) was increased in NK cells at 1 dpi (Figure S32, Supporting Information). Subsequent Stlearn analysis of our scRNA‐seq data to identify possible interactions involving these ligands showed that *Sell* was expressed at the highest levels, and that a *Sell‐Cd34 pair* was distributed in the same spot occupied by NK/cycling MSCs (Figure 8C), implying that NK cell‐expressed *Sell* might promote MSC proliferation through interaction with *Cd34*. Additionally, seven ligand genes in neutrophils (*Ncam1*, *Osm*, *Sell*, *Sema4a*, *Sema4d*, *Thbs1* and *Tnf*) and four in M1 macrophages (*Gdf*, *Nampt*, *Thbs1* and *Tnf*) were identified as upregulated at 1 dpi (Figures S33 and S34, Supporting Information). Moreover, both of these populations showed high expression of *Thbs1* and *Tnf* at the same time, while Stlearn analysis indicated that *Tnf*‐*Tnfrsf1b* and *Thbs1*‐*Cd47* pairs were expressed in the same spots containing neutrophils, M1 macrophages, and cycling MSCs (Figure 8D,E). It should also be noted that *Tnf* and *Cd47* have been reported to promote activation of the JAK‐STAT signaling and cell proliferation,^[^ ^43^, ^44^, ^45^ ^]^ supporting the likelihood that these two signal pairs could contribute to aberrant proliferation of MSCs.

We next explored molecules potentially associated with osteochondral differentiation of MSCs in injured muscle. Regulon analysis revealed that *Runx2*, *Gli1* and *Sox4* could be closely associated with osteogenic differentiation, whereas *Creb3l1* might regulate chondrogenic differentiation in MSCs (Figure 8A). Given our above finding that M2 macrophages, CD4^+^ and CD8^+^ T cells promote aberrant osteochondral differentiation of MSCs, we therefore sought to identify signal molecules from M2 macrophages that could influence cycling MSCs. This analysis identified the *Lgals9*, *Thbs1*, *Spp1*, *Osm* and *Gas6* genes as upregulated at 1 dpi. Although CD4^+^ and CD8^+^ T cells could also interact with cycling MSCs (Table S14, Supporting Information). Although CD4^+^ and CD8^+^ T cells could also interact with cycling MSCs (Figure 6C), we found that *Itga4* and *Mif* were expressed at higher levels in both of these T cell types at 3 dpi compared to uninjured samples (Table S14, Supporting Information). It is noted that *Vcam1* (receptor of Itga4) and *Cd44* (receptor of *Mif*) were reported to promote chondrocyte differentiation.^[^ ^46^ ^]^ We found that *Lgals9* and *Ifng* were expressed at higher levels in both of these T cell types at 3 dpi compared to 1 dpi (Figure 8B). We found that *Lgals9* consistently interacted with cycling MSCs and cMSCs, but not oMSCs, suggesting that the *Lgals9*‐*Cd44* pair could promote chondrogenic differentiation (Figure 8B). Additionally, *Osm* also consistently interacted with cycling MSCs, rather than oMSCs (Figure 8B). Given that *Osm* has been shown to play a role in osteogenic differentiation,^[^ ^47^ ^]^ these results suggested that *Osm* expressed by M2 macrophages cells at 3 dpi might influence cycling MSC differentiation into oMSCs. Stlearn analysis showed that *Lgals9*‐*Cd44* (the latter of which may promote CREB signaling—a likely factor in chondrocyte differentiation^[^ ^48^ ^]^ were expressed in the same spots containing M2 macrophages, cycling MSCs, and cMSCs (Figure 8F). These collective results identified candidate signal molecules specific to each inflammatory immune cell type in injured muscle that could potentially interact with cycling MSCs in close proximity to stimulate their proliferation and differentiation, leading to HO formation. Since *Tnf*‐*Tnfrsf1b* and *Thbs1*‐*Cd47* could activate JAK/STAT signaling, which is also important for transitioning quiescent MSC into the cycling stage according to Regulon analysis (Figure 8A), we revealed that JAK/STAT inhibitor, JAK1‐IN‐11, significantly inhibited the proliferation of MSCs (Figure 8G,H). Regarding osteochondral differentiation of MSCs, we performed the signaling pathway (KEGG) analysis for immune‐MSC interaction molecular pairs using scRNA‐seq and spatial transcriptome database and found that genes associated with PI3K/AKT signaling pathway was enriched in both immune‐cMSCs and immune‐oMSCs interaction, suggesting that PI3K/AKT signaling regulates aberrant osteochondral differentiation of MSC during HO formation (Figure 8I and Figure S35, Supporting Information). Immunofluorescence staining confirmed that PRX1^+^/SOX9^+^ cells and PRX1^+^/RUNX2^+^ cells at 3 dpi expressed higher level of phosphorylated AKT, compared to PRX1^+^/SOX9^−^/RUNX2^−^ MSCs in uninjured tibial muscle and injured tibial at 1 dpi (Figure 8J–M). Moreover, we applied Capivasertib, an inhibitor of AKT kinase, to treat BMP4‐dependent HO mice and traumatic tenotomy HO mice and found that Capivasertib effectively inhibit the HO formation (Figure 8N–Q). These results substantiate the indicated molecular mechanism of HO.

## 3 Discussion

MSCs are heterogenous, multipotent stem cell population that play a well‐established role in muscle and tendon repair after trauma but can also initiate aberrant osteochondral differentiation and eventual heterotopic ossification, the lineage dynamics and cellular molecular mechanisms of which have remained incompletely understood. Here, we found that quiescent MSCs can transition into cycling MSCs before differentiating into two specialized osteo‐ or chondrogenic progenitors, and potentially form heterotopic endochondral bone in soft tissues. Moreover, our results indicate that fate determination in these stem/progenitor cells is regulated by immune cells, including macrophages, neutrophils, NK cells and T cells, in the inflammatory niche. Exploration of immune cell diversity and network interactions suggested that M1 macrophages, neutrophils and NK cells may stimulate cell cycling and proliferation in MSCs, possibly through *Sell*‐, *Tnf*‐ and *Thbs1*‐mediated activation of STAT signaling, whereas *Lgals9* expressed by M2 macrophages and *Ifng* expressed by CD4^+^ and/or CD8^+^ T cells might promote osteochondral differentiation. Characterization of this cellular diversity and post‐injury MSC differentiation trajectory during HO formation suggests that MSC fate determination is mechanistically governed by osteoimmunological signaling from the inflammatory niche and points to several potential therapeutic targets for HO treatment.

The subsets of resident MSCs differ among tissues, and muscle resident MSCs, i.e., fibroadipogenic progenitors (FAP), give rise to myofibroblasts, adipocytes, and osteochondral lineage cells.^[^ ^32^, ^33^ ^]^ ScRNA‐seq and lineage tracing assays in this study revealed that, after muscle or tendon injury, MSCs follow a stepwise developmental progression from quiescent MSCs to cycling MSC at 1 dpi, then differentiate into cMSC or oMSC subpopulations at 3 dpi, which respectively differentiate into chondrocytes at 7 dpi or remain quiescent oMSCs in perichondrium of heterotopic cartilage. It is noteworthy that this aberrant MSC differentiation we observed in soft tissues strongly resembles the progression of endochondral bone formation occurring under physiological conditions.^[^ ^49^ ^]^ We also observed that MSC lineage cells showed highest outgoing and incoming interactions with other cell types, i.e., they potentially regulated immunogenicity of immune cells and could also be contingent on immune cells. Cycling MSCs showed highest incoming interaction score, which represents they will receive most ligands and activate associated signaling pathways during HO formation; this raises the central hypothesis that immune cells is critical to trigger quiescent MSC transition and cycling MSC differentiation. cMSCs and oMSCs emerged after cycling MSC and also interacted with immune cells, which could initiate the aberrant osteochondral differentiation.

Skeletal muscle tissues are known to retain a larger pool of quiescent stem or progenitor cells for tissue repair than other tissues,^[^ ^50^ ^]^ which is consistent with our results showing rapid MSC proliferation in injured muscle of HO model mice. Thus, inhibiting MSC proliferation, for instance by blocking STAT pathway activation via targeting the *Sell*‐*CD34*, *Tnf*‐*Tnfrsf1b* and *Thbs1*‐*Cd47* ligand‐receptor pairs, might suppress HO but could also hinder repair of skeletal muscle. Results in this work show that STAT pathway genes are upregulated in cycling MSCs after muscle or tendon injury, especially targets of *Stat3* and *Stat1* known to play a role in cell proliferation among other processes.^[^ ^51^ ^]^ Interestingly, inhibition of JAK1/2 could prevent neurogenic HO.^[^ ^52^ ^]^ We noted that both traumatic HO and FOP share some common features in pathology, i.e., injury‐induced inflammatory response will promote or accelerate heterotopic endochondral bone formation in the soft tissues. However, there are still difference between two types of HO, *i.e*., FOP is caused by gain‐of‐function mutations of ACVR1 and subsequent hyperactivation of BMP signaling pathway, whereas tHO might be closely associated with TGFβ signaling activation.^[^ ^53^ ^]^ Future work will investigate whether any of these STAT pathway molecules might serve as specific targets for alleviating HO.

Upregulation of *Cd44* in cycling MSCs could activate *Creb3l1* and/or *Runx2* expression, which respectively promote chondrogenic and osteogenic differentiation. Since *Cd44* is expressed in both chondrocytes and osteoblasts, we speculated that *Cd44* signaling could be involved in osteochondral differentiation. Furthermore, disrupted CD44 expression (fragment) is enhanced in osteoarthritis (OA) patients.^[^ ^48^ ^]^ We found that M2 macrophages at the injury site express *Lgals9*, which could activate *Cd44* on nearby cycling MSCs, and macrophages reportedly promote HO formation, suggesting *Cd44* participates in both chondrogenic and osteogenic differentiation. The OSM‐OSMR pair has also been shown to play an essential role in osteochondral differentiation,^[^ ^47^ ^]^ and was especially activated during aberrant chondrogenic differentiation in this study. Our evidence showing that the same pathways for endochondral bone formation are involved in HO suggest that these targets might be exploited for engineering bone regeneration in future therapeutic development.

So‐called “niches” include both specific anatomical locations as well as the resident stem and immune cells required to regulate tissue generation, maintenance, and repair.^[^ ^54^ ^]^ Niche populations serve as a reservoir to mitigate depletion, provide context/signaling to ensure functionality of its specific constituent somatic stem cell populations, and suppress excessive proliferation via signaling that balances stem cell proliferation and fate determination with host requirements.^[^ ^55^ ^]^ Tissue‐resident stem cells display remarkable plasticity in damaged regions where inflammatory cells also accumulate to form the inflammatory niche in reparative responses.^[^ ^56^ ^]^ MSCs have the capacity for multipotent differentiation, especially in HO formation, and future work will explore the full diversity of the in vivo MSC niche. We identified several immune cells recruited to the lesion that form a niche with resident MSCs. Our scRNA‐seq and spatial transcriptomic analysis revealed that the immune cell types interacting with MSCs differ among MSC developmental stages. Macrophage‐derived TGFb1, activin A, and BMPs are essential for osteochondral differentiation in MSCs.^[^ ^57^ ^]^ In addition, macrophages exhibit dynamic shifts in their role in the inflammatory niche, with M1 macrophages inducing apoptosis in cartilage while M2 macrophages promote cartilage hypertrophy and ectopic bone formation.^[^ ^49^ ^]^ However, our results indicate that M1 macrophages consistently interact with quiescent and cycling MSCs after muscle injury to promote proliferation and preferential osteochondral differentiation in HO development. Moreover, the M1 macrophage population transitions to an M2 type, promoting chondrocyte and osteocyte formation.

We noted that macrophages (clusters 3 and 6) in uninjured state expressed *Mrc1* and could be associated with tissue homeostasis maintenance as a population of tissue‐resident macrophages. Clusters 4 and 5 consisted of monocytes, likely serve as a reservoir for tissue‐specific macrophages. After injury, cluster 2 monocytes and cluster 1 macrophages expressed *Tnf* and increased rapidly, indicating their role as inflammation‐responsive M1 monocytes/macrophages. However, at 3 dpi, clusters 3, 4, 5 and 6 were reassembled. Particularly, cluster 3 macrophages (*Mrc1* ^+^) showed a significant increase at 3 dpi, suggesting that these macrophages were initiating the repair process. By 7 dpi, tissue‐repairing macrophages persisted at levels comparable to those in uninjured tissue, indicating a completion of the macrophage‐mediated tissue repair process. Therefore, the function of macrophages undergoes dynamic changes compared to the uninjured state.

Additionally, we found that M1 macrophages, but not M2 macrophages, occurred only at 1 dpi. Pre‐treatment of clodronate could inhibit the M1 macrophages at 1 dpi. At this time point (1 dpi), M1 macrophage–MSC interaction was examined, without perturbation of M2 macrophages. When explore the interaction between M2 macrophages and MSCs, we applied MRC1 neutralizing antibody to specifically inhibit M2 macrophages. Considering that M1 and M2 macrophages respectively affect MSCs proliferation and osteochondral differentiation, we used clodronate to deplete both M1 and M2 macrophages along proliferation and differentiation stage and hope to inhibit HO formation and in this experiment, clodronate indeed potentially inhibit the M1 macrophages at early stage and subsequent occurrence of M1‐derived M2 macrophages, but this result demonstrated that macrophages’ contribution in HO formation. These findings underscore the critical role of macrophages in tissue repair and their potential impact on HO formation.

Taken together, we revealed a stepwise amplifying strategy for osteochondral differentiation of MSCs during HO formation. The disclosure of spatiotemporal patterning of inflammatory niche at single‐cell resolution provides key insights into the intrinsic nature of MSC and their progeny as well as the osteoimmunological interaction for networking and functioning, although the molecular mechanism of environmental cues in shaping MSC development requires further exploration. Our study will provide the molecular landscape of osteoimmunological interactions that occur during aberrant osteochondral differentiation that could be useful for HO treatment.

## 4 Experimental Section

### Mice

*Nse‐Bmp4* transgenic mice (C57BL/6 background) were provided by Dr Kan Lixin (Northwestern University, USA). As described previously, the *Nse‐Bmp4* transgene was constructed by cloning a 1246‐bp fragment containing the murine *Bmp4* cDNA downstream of the rat neuron‐specific enolase (*Nse*) promoter and upstream of an SV40 polyadenylation signal. C57BL/6 background *Tek‐Cre*, *LysM‐Cre*, *Itgax‐Cre*, *Lck‐Cre*, *Rosa26^mT/mG^* and *Rosa26^iDTR^* mice were purchased from Jackson laboratories. *Ly6G‐Cre* mice were purchased from Shanghai Model Organisms Center, Inc. *Tek‐Cre* mice were mated with *Rosa26^mT/mG^* mice to obtain *Tek‐Cre*; *Rosa26^mT/mG^* mice for tracing *Tek* ^+^ MSCs development after soft tissues injury. *Tek‐Cre*, *LysM‐Cre*, *Itgax‐Cre*, *Lck‐Cre* and *Ly6G*‐*Cre* mice were mated with *Rosa26^iDTR^* mice to obtain *LysM‐Cre*; *Rosa26^iDTR^*, *Itgax‐Cre*; *Rosa26^iDTR^*, *Lck‐Cre*; *Rosa26^iDTR^* and *Ly6G*‐*Cre*; *Rosa26^iDTR^* mice respectively. All mice were bred and maintained under SPF conditions with 12 h dark/light cycle, regular chow diet, 24 °C temperature, and 60% humidity. All experimental procedures involving mice were carried out as prescribed by the National Guidelines for Animal Usage in Research (China) and were approved by the Ethics Committee of Anhui Medical University (reference: LLSC20210807; Hefei, China).

### HO Model Mice

BMP4‐dependent HO was induced by Cardiotoxin (CTX) injury of tibial muscle in *Nse‐Bmp4* mice, according to previous reports.^[^ ^17^ ^]^ For tenotomy HO model, mice were anesthetized with 1% pentobarbital sodium. The skin was incised to expose the Achilles tendon. The Achilles tendon received 20 times of repeated clamping by the hemostatic forceps and then was cut by the scissors. Finally, the skin was closed with sutures.^[^ ^58^ ^]^

### scRNA‐seq Data Preprocessing

Uninjured and injured tibial muscles at 1, 3 and 7 dpi from *Nse‐Bmp4* mice (3 tibial muscles were mixed for one group) were harvested and performed the scRNA‐seq assay using 10× genomics platform. The Cell Ranger software pipeline (10× Genomics, version 5.0.0) was used to demultiplex cellular barcodes, map reads to the genome and transcriptome using the STAR aligner, and down‐sample reads as required to generate normalized aggregate data across samples, producing a matrix of gene counts versus cells. Using the R package Seurat (version 3.1.1), the unique molecular identifier (UMI) count matrix was processed. To remove low‐quality cells and likely multiplet captures, cells were filtered out with UMI/gene count values that were more than 2 standard deviations from the mean, assuming a Gaussian distribution of each cells' UMI/gene numbers. Visual inspection of cell distributions was used to further discard low‐quality cells where >10% of the counts belonged to mitochondrial genes. Additionally, the DoubletFinder package (version 2.0.2) was applied to identify potential doublets. After applying these quality control criteria, single cells were included in downstream analyses. Library size normalization with the NormalizeData function in Seurat was performed to obtain the normalized count. Specifically, the global‐scaling normalization method was used “LogNormalize,” which normalized the gene expression measurements for each cell by the total expression, then multiplied by a scaling factor (10 000 by default) and log‐transformed the results. Top variable genes across single cells were identified using the method described in Macosko et al. The most variable genes using the FindVariableGenes function were selected (mean.function = FastExpMean, dispersion.function = FastLogVMR) in Seurat. The RunPCA function was then used in Seurat to perform principal component analysis (PCA) to reduce the dimensionality [A4] of the data. Finally, graph‐based clustering was performed to cluster cells according to their gene expression profile using R based on the hypergeometric distribution.

### Regulon Analysis

Regulon analysis was performed by The SCENIC package, which was run using the motifs database for RcisTarget and GRNboost with the default parameters. In detail, transcription factor (TF) binding motifs over‐represented on a gene list with RcisTarget package were identified. The activity of each group of regulons in each cell was scored by AUCell package. To evaluate the cell type specificity of each predicted regulon, the regulon specificity score (RSS) was calculated, which was based on the Jensen‐Shannon divergence (JSD), a measure of the similarity between two probability distributions. The connection specificity index (CSI) for all regulons was calculated with the scFunctions (https://github.com/FloWuenne/scFunctions/) package.

### Spatial Transcriptome Analysis

Uninjured and injured tibial muscles at 1, 3 and 7 dpi from *Nse*‐*Bmp4* mice were cut into s 4–5 mm pieces and treated with cleanroom wipers. After removing blood stains on the tissue surface, fresh tissue was embedded with OCT then snap frozen at −80 °C. Cryosections were cut at 10 µm thickness, mounted onto the ST arrays. Next, the tissue was dehydrated with isopropanol for 1 min followed by staining with H&E. Slides were mounted in 80% glycerol and brightfield images were taken on 3D HISTECH Pannoramic MIDI FL, whole‐slide scanner at 40× resolution. Unstaining cryosections were then for library preparation, tissue optimization, on‐slide tissue permeabilization, cDNA synthesis, library construction and sequencing. Finally, the generated FASTQ files were processed and aligned to mouse reference genome using Space Ranger software (version 2.0.1) from 10X genomics, with unique molecular identifier (UMI) counts summarized for each barcode. To distinguish tissue overlaying spots from the background, tissue overlaying spots were detected according to the images. The filtered UMI count matrix was then analyzed using Seurat (version 4.1.0) R package. To infer the cell‐type composition of each spot, SPOTlight (version 0.1.2) was applied with default parameters. Specifically, A non‐negative matrix decomposition (NMF) based deconvolution algorithm was used to infer the cell composition of each spot by combining single‐cell transcriptome data (scRNA‐seq) and cell type marker gene information with spatial transcriptome data. The diameter of a spot is 50 µm. there are on average 1–10 cells per spot depending on tissue type. Differentially expressed genes (DEGs) were selected using the Seura function FindMarkers (test.use = presto). *P* value < 0.05 and |log2foldchange| > 0.58 was set as the threshold for significantly differential expression. GO enrichment and KEGG pathway enrichment analysis of DEGs were respectively performed using R (version 4.0.3) based on the hypergeometric distribution.

### MicroCT

Mouse hindlimbs were harvested and imaged after injury at different time points. To quantitatively measure the HO volume and bone parameters, microCT (Skyscan 1176, Bruker) was used with the setting parameters of 180° rotation, constant 90 kV voltage, and a voxel size of 72 µm. 3D images were reconstructed with SkyScan software. The HO region was first outlined by the ROI module and then quantified by individual 3D object analysis.

### Histology Analysis

WT and *Nse‐Bmp4* mouse hindlimbs with or without injury were harvested at different time points and then were subjected to the indicated staining. Briefly, hindlimbs were fixed at 4 °C in 4% paraformaldehyde or 10% neutral‐buffered formalin. Samples were then decalcified in 20% (m/v) EDTA solution for 4–6 weeks at 4 °C until deformable manually. Bones were paraffin‐embedded and 10 µm sections were cut and stored at −20 °C. Paraffin sections were selected for dewaxing and then HE staining (HE staining Kit, G1120, Solaribio), Saffron‐O staining (modified safranine O‐fast green FCF cartilage staining Kit, G1371, Solarbio) and Masson trichrome staining (Masson trichrome staining kit, BP‐DL023, Sbjbio) assays were performed according to the manufacturer's instructions.

### Immunofluorescence Assays

Immunostaining for different markers was performed as previously described.^[^ ^59^ ^]^ Briefly, sections were prefixed with 4% paraformaldehyde in PBS. Nonspecific binding was blocked with 10% normal goat serum diluted in 1% bovine serum albumin (V900933, Sigma) and 0.25% Triton X‐100 (X100, Sigma) for 1 h at room temperature. For CldU staining, one day before injury, CldU (42.5 mg kg^−1^) was injected intraperitoneally into adult (>1 month old) *Nse‐Bmp4* mice three times per day. After CTX injury or tenotomy injury at indicated time point, the mice were sacrificed, and the tissues were harvested. sections were first denatured by incubating the sections in 2 n HCl for 30 min at 37 °C, neutralized with 0.1 m sodium borate, pH 8.5, for 10 min at room temperature, rinsed twice with PBS. Then, sections were then incubated with primary antibodies diluted with 1% BSA + 0.25% Triton X‐100 at 4 °C overnight (the primary antibodies used in this study are listed in Table S15, Supporting Information). After washing, the sections were incubated with appropriate secondary antibodies (Alexa Fluor 488‐ or Alexa Fluor 594‐conjugated antibodies) diluted with 1% BSA + 0.25% Triton X‐100 in the dark at room temperature for 2 hand counterstained with 4,6‐diamidino‐2‐phenylindole (1:4000). Other immunostaining assays were directly performed from blocking step. All fluorescence microscopy images were acquired using a ZEISS Axio Observer (Carl Zeiss).

### Drugs or Neutralizing Antibodies Treatment In Vivo

C57BL/6 background *Nse‐Bmp4* and/or WT mice (*n* = 4–6) were treated with either clodronate or PBS liposomes (CP‐005‐005, 200 µL per mouse, Liposoma) through i.p. injection according to the protocols in the indicated experiments. Regarding neutrophils, CD3^+^, CD4^+^ and CD8^+^ T cells depletion, injured *Nse‐Bmp4* mice with neutralizing antibodies were treated, including Ly6G (BE0075‐1, 500 µg in 100 µL PBS per mouse, BioXCell), CD3 (A2104, 50 µg in 100 µL PBS per mouse, Selleck), CD4 (A2101, 200 µg in 100 µL PBS per mouse, Selleck), CD8 (A2102, 200 µg in 100 µL PBS per mouse, Selleck) and Asialo GM1 (986‐10001, 20 µL per mouse, Wako Pure Chemical corporation) via tail vein injection at the indicated time points. Inhibitors of JAK (MCE, JAK1‐IN‐11) and AKT (MCE, Capivasertib) were i.p. injected into the indicated mice. The readouts of these treatments included a) the numbers of MSCs and their subpopulations at different time points and b) microCT parameters (for HO volume quantification analysis).

### Flow Cytometry

Quantification of macrophages and myeloid cells was performed by flow cytometry. In brief, injured site cells of mouse muscle and tendon were harvested, washed, and incubated for 20 min at 4 °C in phosphatase buffered saline (PBS) containing the anticipated antibodies (see Table S15, Supporting Information). Next, the cells were analyzed using Cytoflex (Beckman).

### Statistical Analysis

Unless otherwise noted, data are presented as the mean ± s.d. of biological replicates (independent animals/independent experiments; the numbers (*n*) are specified in each figure legend). Unpaired two‐tailed Student's t‐tests were used to determine the significance of differences between two groups. One‐way ANOVA is applied into multiple comparisons with a single factor. *P* < 0.05 was considered statistically significant (* *p* < 0.05; ** *p* < 0.01; *** *p* < 0.001, and **** *p* < 0.0001).

### Data Availability

The RNA‐sequencing data are deposited in GEO (accession number: GSE246445 and GSE246448). scRNA‐seq data of tibial muscle in normal mice were from GEO database (GSE138826). The antibodies used are shown in Table S15 (Supporting Information). The data that support the findings of this study are available from the corresponding author upon request. Additionally, images in Graphic abstract and Figure 1A were created in BioRender. com (Lience: BioRender. com/z20a072).

## Conflict of Interest

The authors declare no conflict of interest.

## Author Contributions

C.K. and Z.T. contributed equally to this work. H.Z., C.K., and H.F. conceived and conducted the project. H.Z. supervised the project. C.K. and H.Z. wrote the paper. C.K., Z.T., W.W., H.F., J.Y., Y.Z., X.L., Q.C., L.C., C.P., L.Z., K.L., J.Z., C.Z., H.W., and Y.L. performed the experiments and data analysis. H.W. and L.Z. contributed to the experimental design and execution.

## Supporting information



Supporting Information

Supporting Tables

## Data Availability

The data that support the findings of this study are openly available in GEO at https://www.ncbi.nlm.nih.gov/geo/, reference number 246445, 246448 and 138826.
