# Supplementary material for: Spatiotemporal Analysis of Mesenchymal Stem Cells Fate Determination by Inflammatory Niche Following Soft Tissue Injury at a Single‐Cell Level
Source: Adv Sci (Weinh). 2024 Sep 23;11(43):2310282. doi: 10.1002/advs.202310282 (PMC11578311; doi:10.1002/advs.202310282)
Supplement: Supplementary file 1 — Supporting Information [file ADVS-11-2310282-s001.docx]

**Supplemental information**

**Spatiotemporal analysis of mesenchymal stem cells fate determination by** **inflammatory niche following soft tissue injury at a single-cell level**

**Chen Kan, Zhenya Tan, Haitao Wang, Wei Wang, Jiazhao Yang, Ya Zhang, Xiaoling Lu, Qirong Cheng, Lanyi Chai, Chao Peng, Jicheng Zhu, Chenghang Zhu, Hailin Wang, Li Zhan, Keqiong Lin, Yakun Liu, Lingqiang Zhang, Haitao Fan, Hong Zheng**


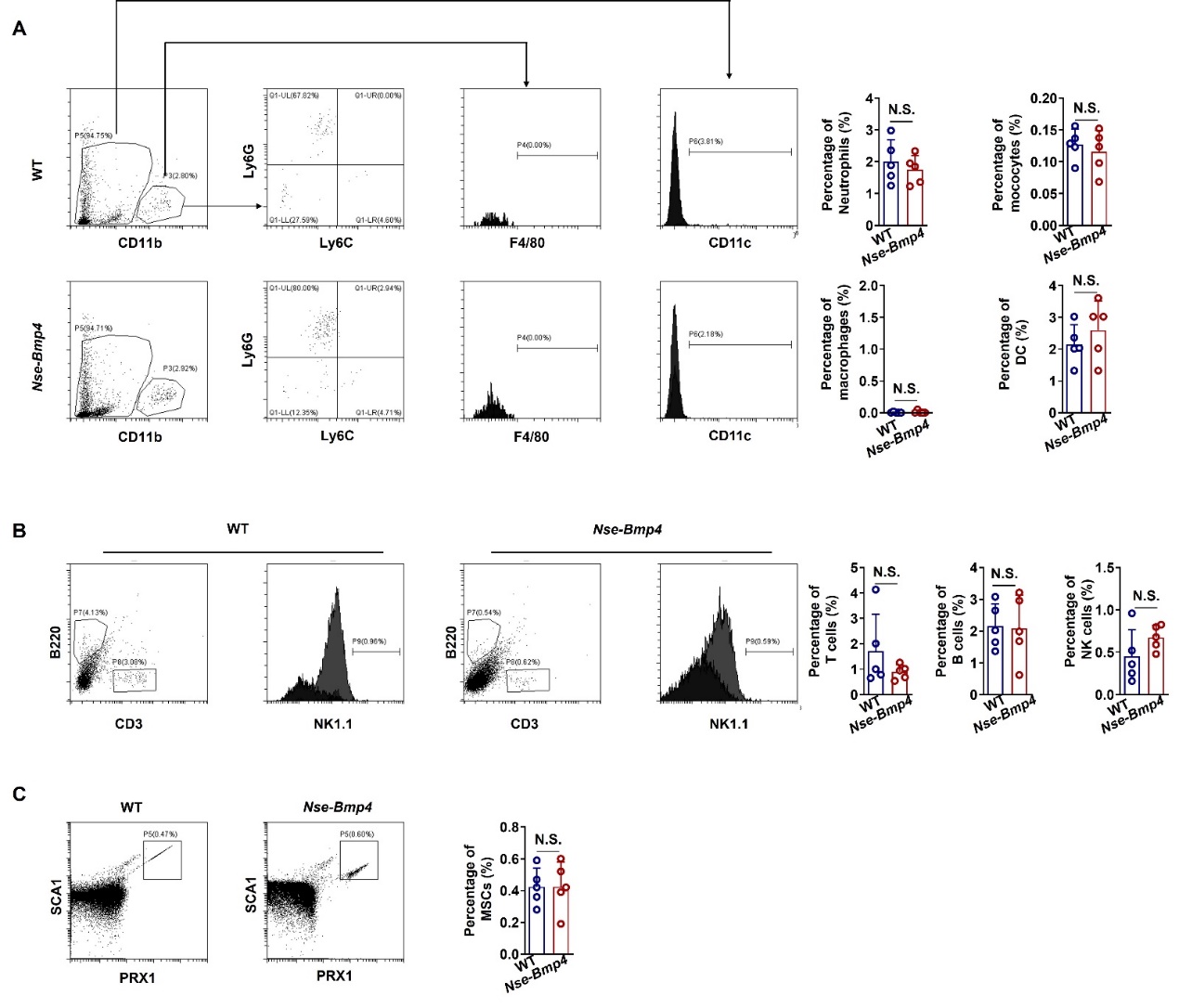


**Figure S1. There was no significant difference in the profile of immune cell and MSCs between uninjured WT and *Nse*-*Bmp4* mice**. (**A**) Representative flow cytometry analysis and statistical analysis of innate immune cells in uninjured tibial muscle of WT and *Nse-Bmp4* mice (n=5 per group). Data are presented as mean ± SD of biological replicates. N.S. indicated no significance. (**B**) Representative flow cytometry analysis and statistical analysis of adaptive immune cells in uninjured tibial muscle of WT and *Nse-Bmp4* mice (n=5 per group). Data are presented as mean ± SD of biological replicates. N.S. indicated no significance. (**C**) Representative flow cytometry analysis and statistical analysis of PRX1^+^/SCA1^+^ MSCs in uninjured tibial muscle of WT and *Nse-Bmp4* mice (n=5 per group). Data are presented as mean ± SD of biological replicates. N.S. indicated no significance.


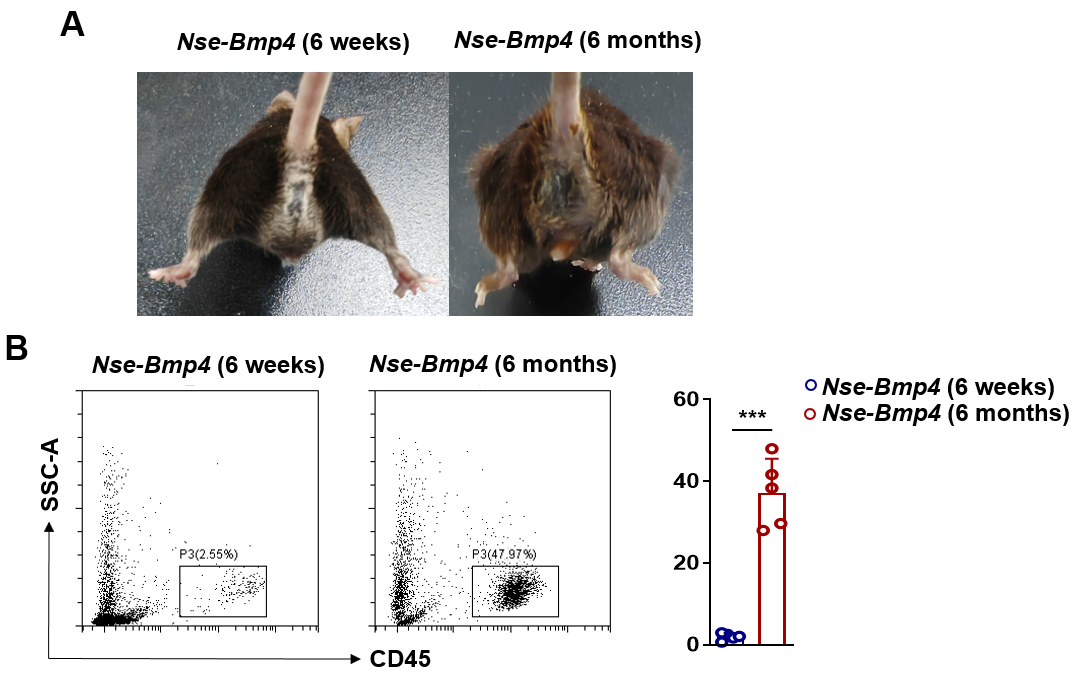


**Figure S2. Identification of the limb phenotype of *Nse*-*Bmp4* mice**. (**A**) Representative hind limb images of uninjured *Nse-Bmp4* mice at 6-weeks old and 6-months old. (**B**) Representative flow cytometry analysis and statistical analysis of CD45^+^ cells in tibial muscle of *Nse-Bmp4* mice at 6-weeks old and 6-months old (n=5 per group). Data are presented as mean ± SD of biological replicates. *** *p* < 0.001.


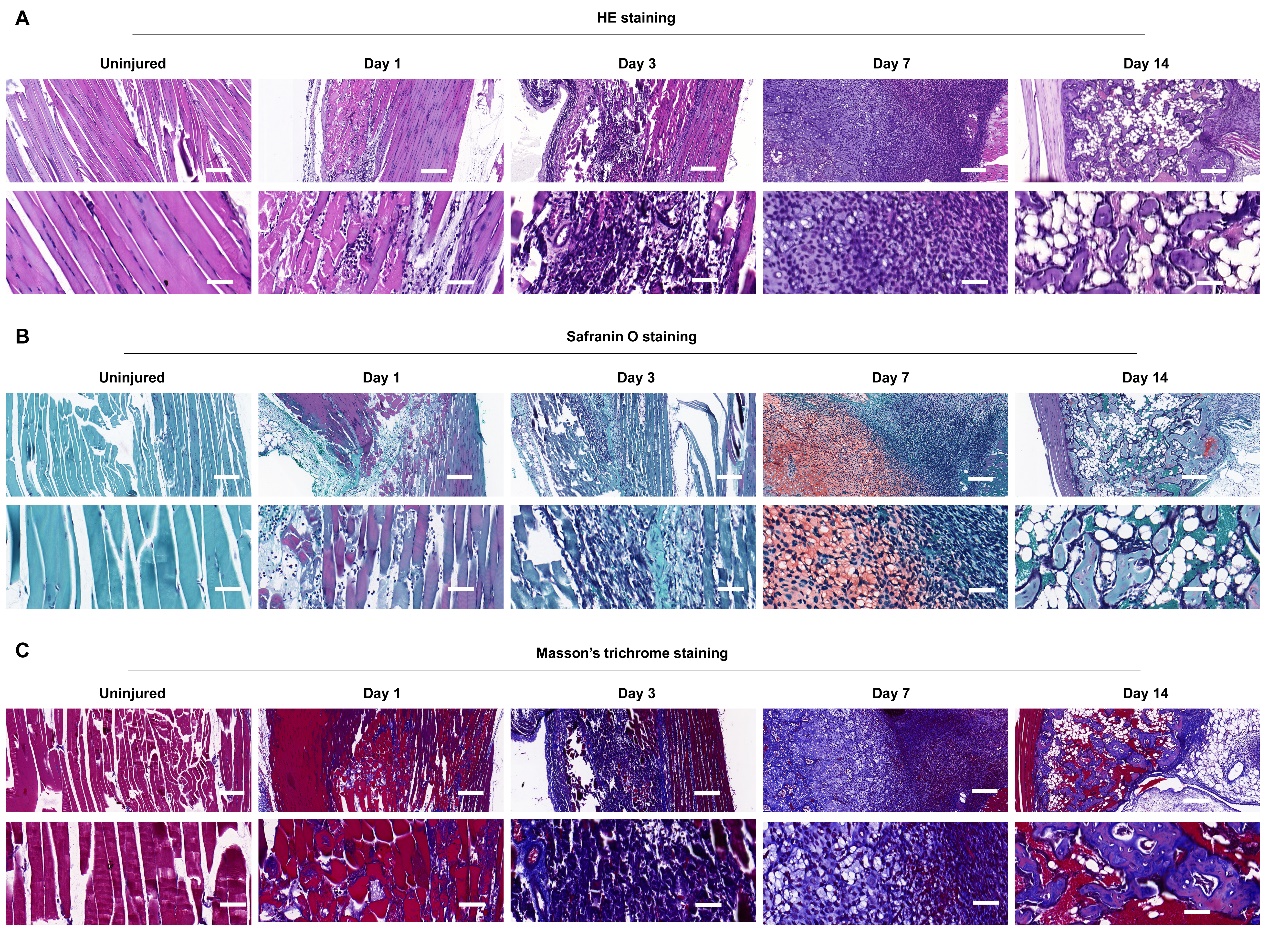


**Figure S3. Histologic analysis of the HO development in tibial muscle of *Nse-Bmp4* mice following injury**. (**A**) Representative HE staining images of uninjured and injured tibial muscle of *Nse-Bmp4* mice at different time points post injury (*i.e*., 1, 3, 7 and 14 dpi). (**B**) Representative Safranine O and fast green staining images of uninjured and injured tibial muscle of *Nse-Bmp4* mice at different time points post injury (*i.e*., 1, 3, 7 and 14 dpi). (**C**) Representative Masson staining images of uninjured and injured tibial muscle of *Nse-Bmp4* mice at different time points post injury (*i.e*., 1, 3, 7 and 14 dpi).


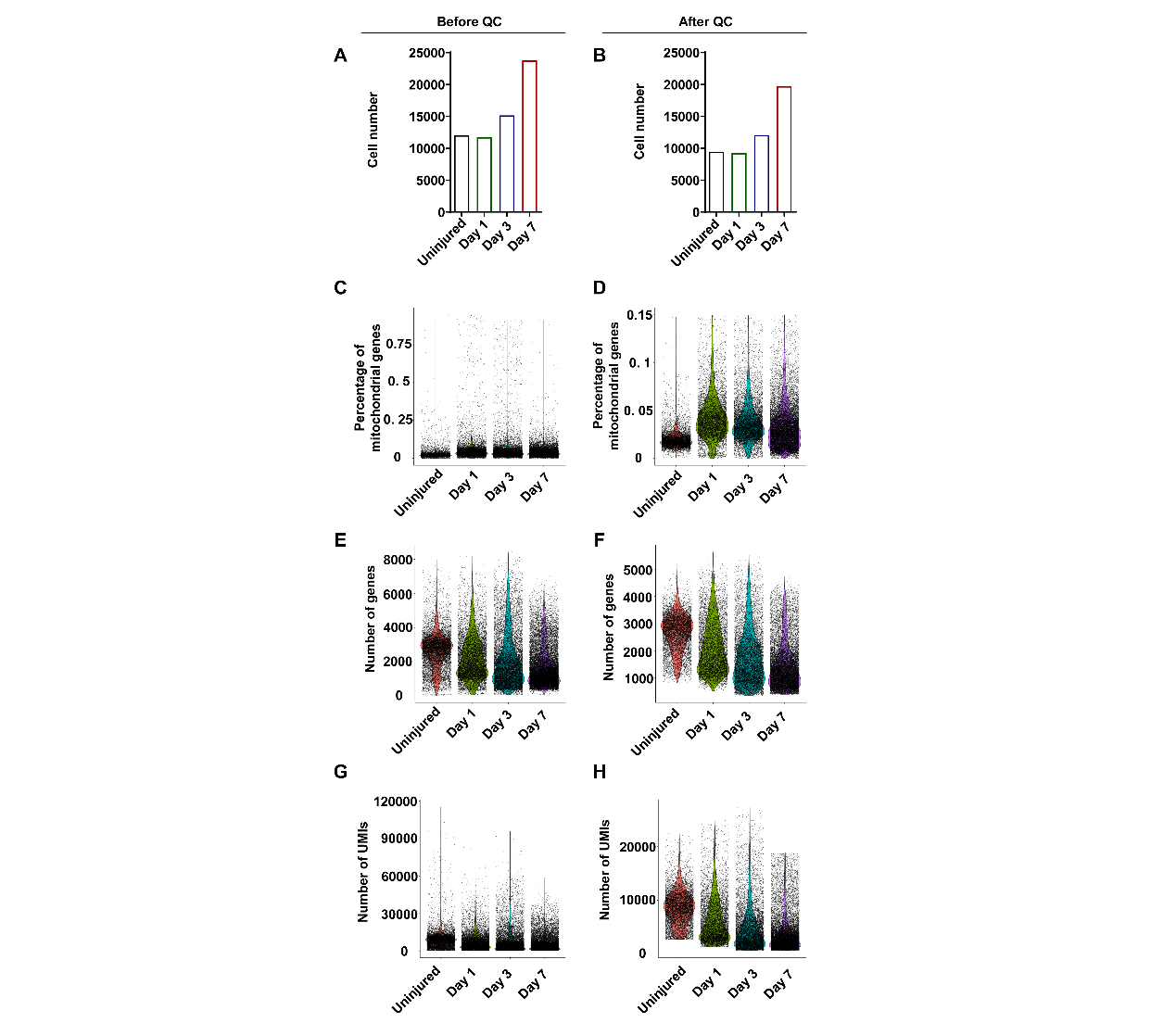


**Figure S4. Quantification of the cells from tibial muscle of *Nse-Bmp4* mice for scRNA-seq assay**. (**A**) Bar plot showing the number of sequenced cells collected at different time points. (**B**) Bar plot showing the number of qualified cells for further bioinformatics analyses. (**C-H**) Quantification of the percentage of mitochondrial genes, number of UMIs and number of detected genes per cell before (**C, E and G**) and after (**D, F and H**) cell filtering.


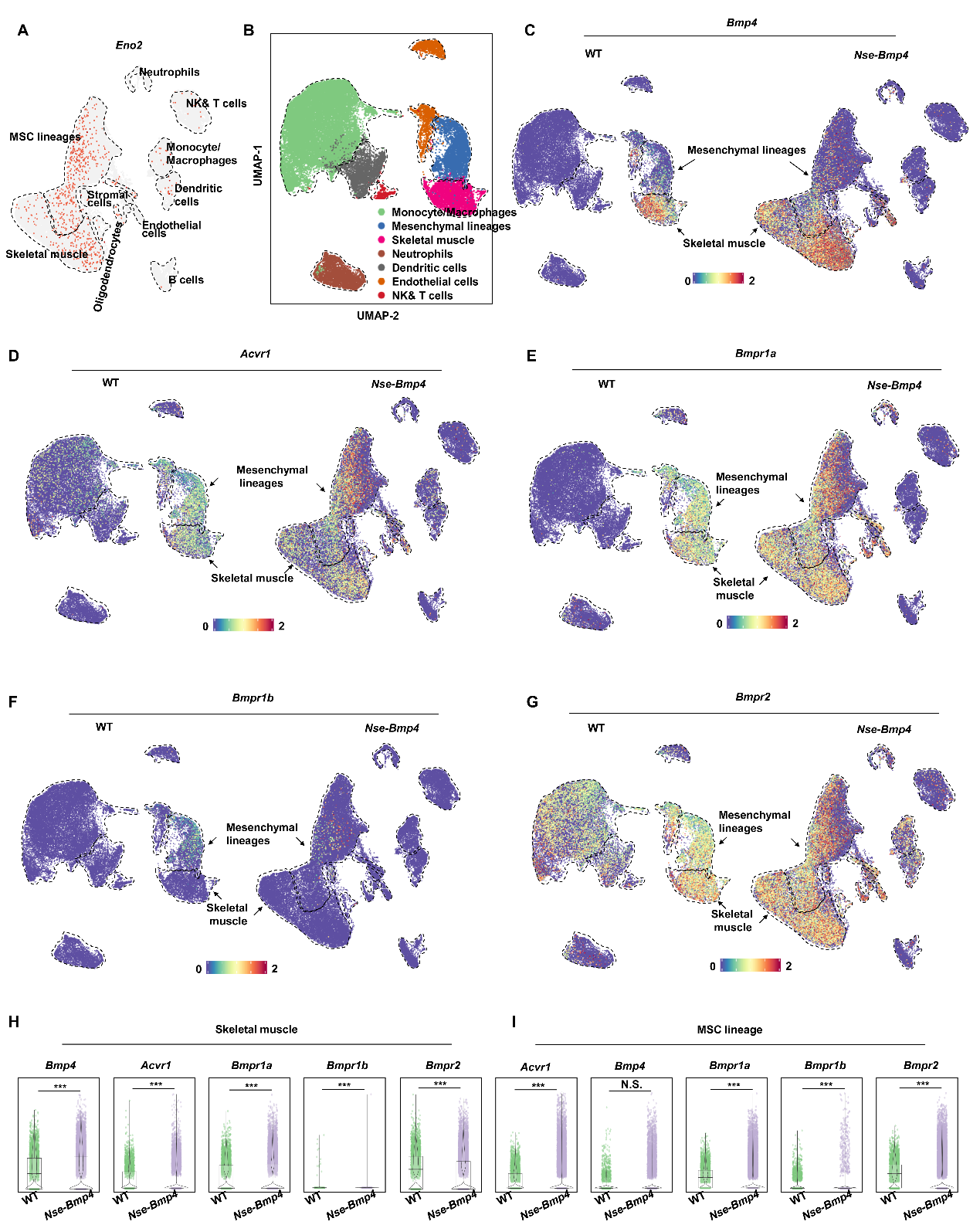


**Figure S5. The expression of BMP signaling pathway-associated genes increases in *Nse*-*Bmp4* mice using scRNA-seq analysis.** (**A**) Feature plot of *Nse* (*Eno2*) in skeletal muscle of *Nse*-*Bmp4* mice with or without injury. (**B**) UMAP visualization of diverse cell types in skeletal muscle of WT mice with or without injury. (**C-G**) Feature plot images of *Bmp4* (**C**), *Acvr1* (**D**). *Bmpr1a* (**E**), *Bmpr1b* (**F**) and *Bmpr2* (**G**) in tibial muscle of WT and *Nse*-*Bmp4* mice with or without injury. (**H, I**) Viloin plot images of *Bmp4*, *Acvr1*. *Bmpr1a*, *Bmpr1b* and *Bmpr2* in muscle cells (**H**) and MSC lineage cells (**I**) of tibial muscle of WT and *Nse*-*Bmp4* mice.


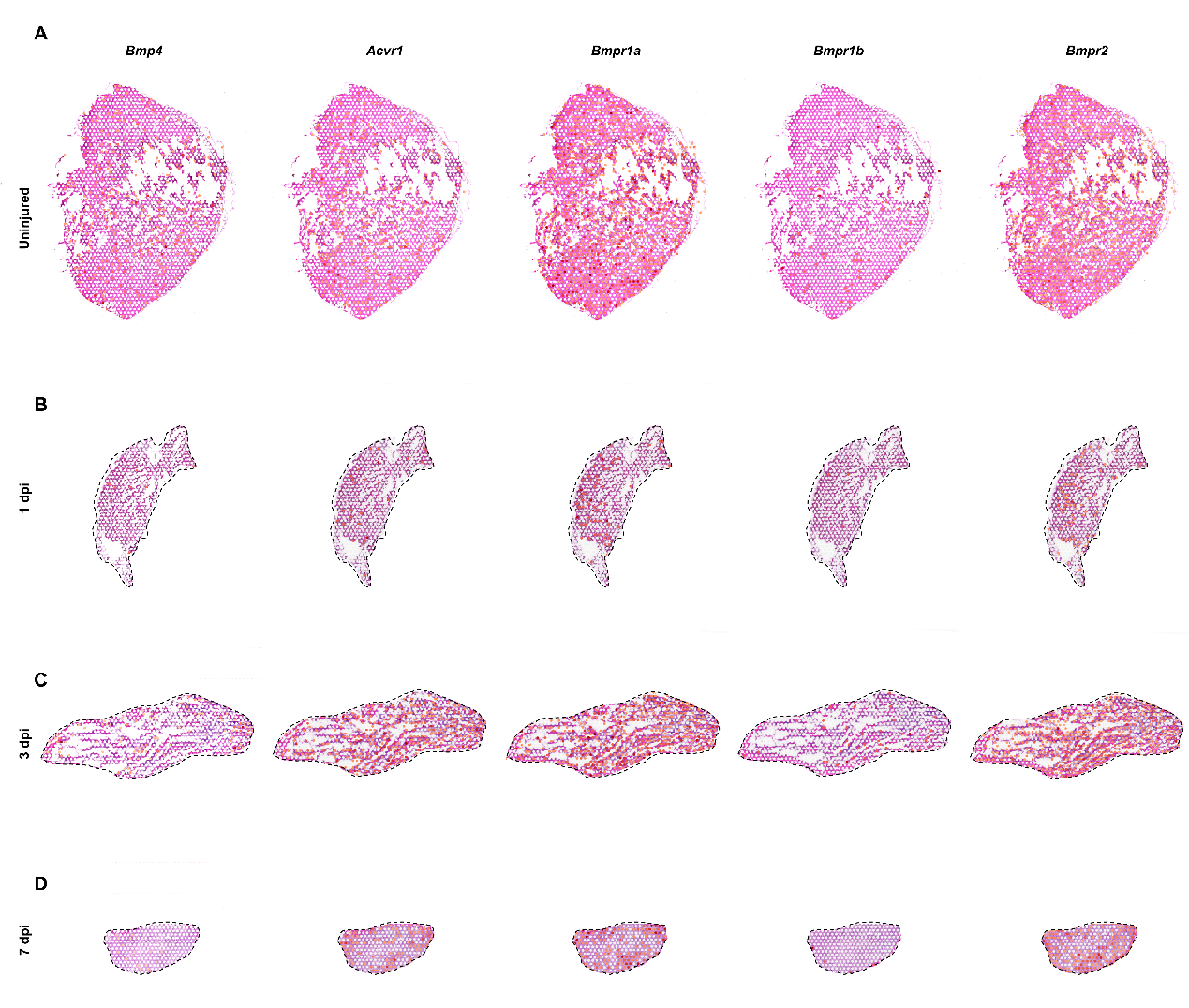


**Figure S6. Spatial transcriptome analysis of BMP signaling-associated genes in injured sites of *Nse*-*Bmp4* mice.** (**A-D**) Spatial expression of *Bmp4*, *Acvr1*. *Bmpr1a*, *Bmpr1b* and *Bmpr2* in uninjured (**A**) or injured site of *Nse-Bmp4* mice at1 (**B**), 3 (**C**) and 7 (**D**) dpi.


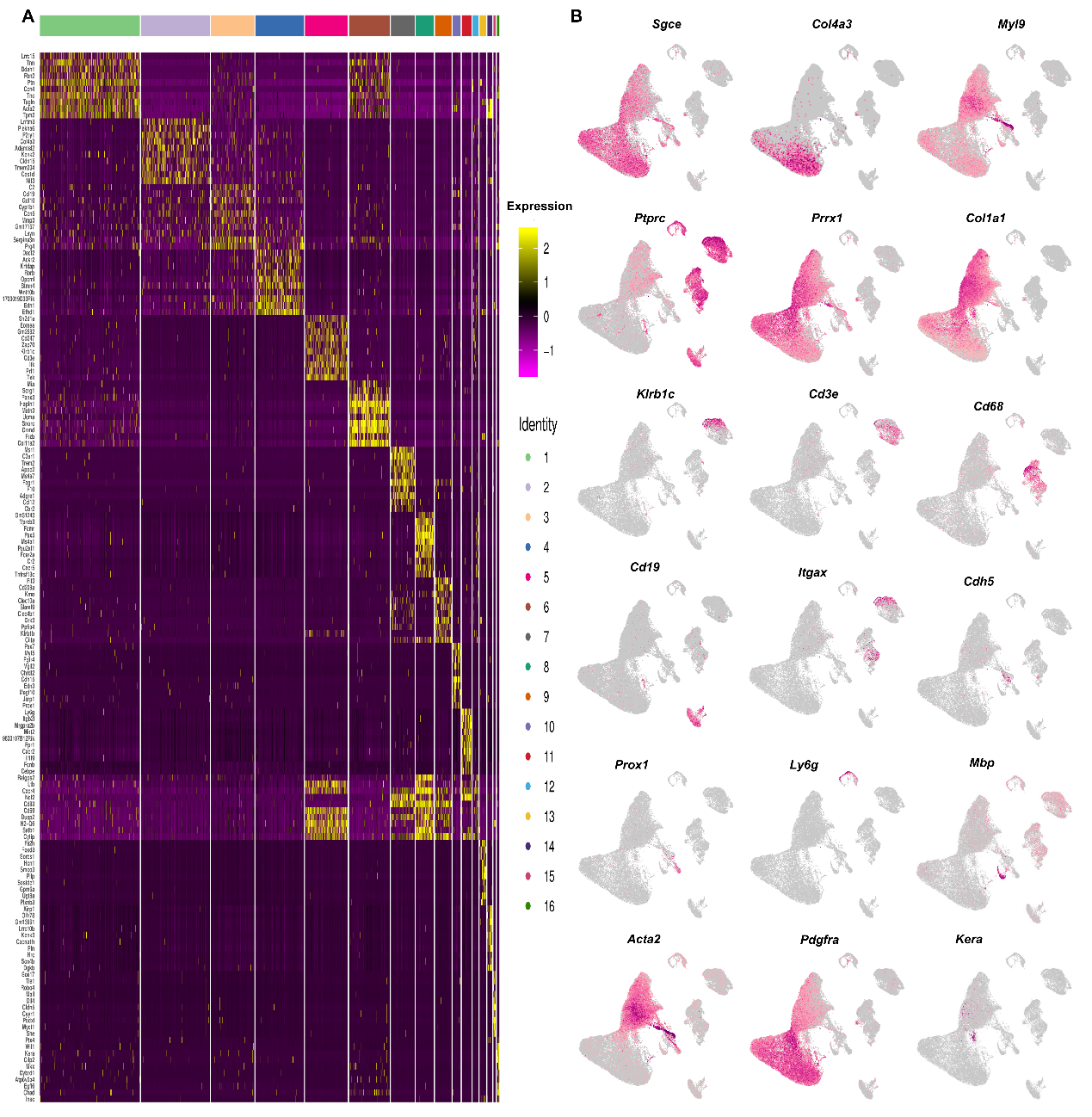
**Figure S7. Identification of the cell types involved in BMP4-dependent HO formation in muscle.** (**A**) Heatmap showing the top 10 differentially expressed genes, which is enriched in diverse cell types of tibial muscle of *Nse*-*Bmp4* mice. (**B**) UMAP visualization of diverse cell types from our dataset, including mesenchymal lineage cells (*Prrx1*^+^/*Col1a1*^+^/*Ptprc*^-^), skeletal muscle cells (*Sgce*^+^/*Col4a3*^+^/*Myl9*^+^/*Ptprc*^-^), NK & T cells (*Klrb1c*^+^ or *Cd3e*^+^), monocytes/macrophages (*Cd68*^+^), B cells (*Cd19*^+^), Dendritic cells (*Itgax*^+^), endothelial cells (*Cdh5*^+^ or *Prox1*^+^), neutrophils (*Ly6g*^+^), oligodendrocytes (*Mbp*^+^), smooth muscle cells (*Acta2*^+^/*Pdgfra*^-^), and stromal cells (*Kera*^+^).


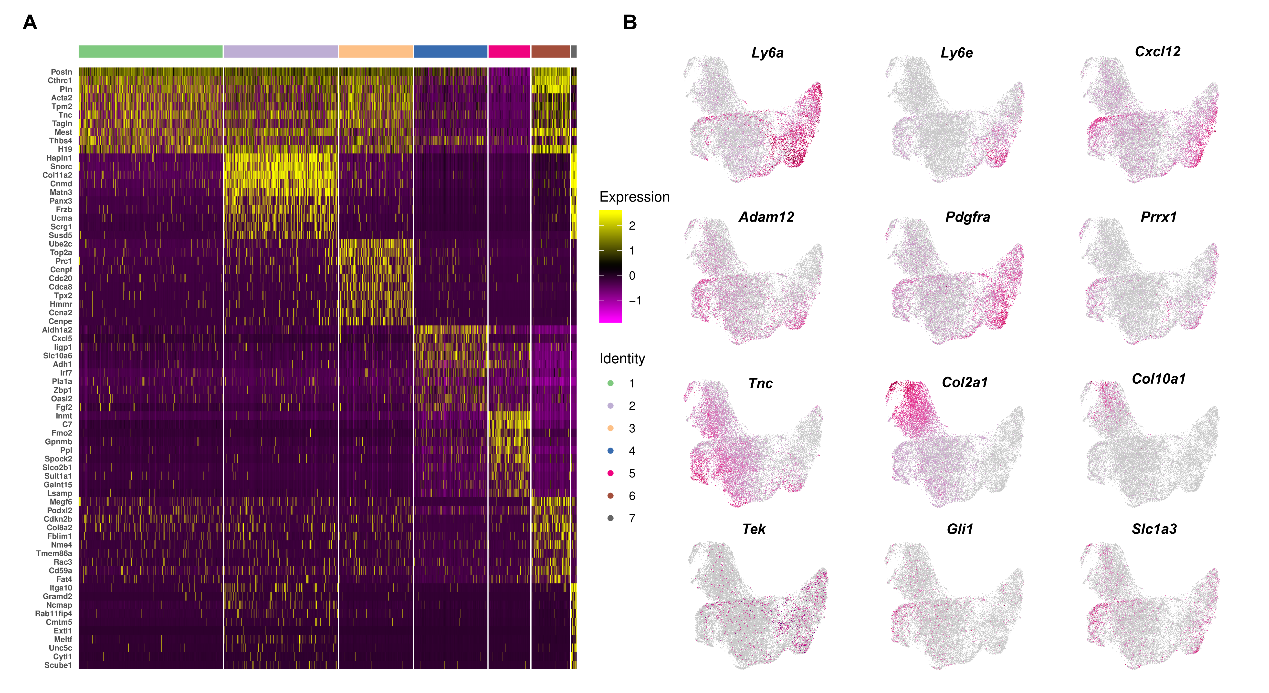


**Figure S8. Identification of the MSCs in tibial muscle of *Nse-Bmp4* mice with or without injury.** (**A**) Heatmap showing the top 10 differentially expressed genes enriched among diverse types of MSCs and their descendants in tibial muscle of *Nse*-*Bmp4* mice. (**B**) UMAP visualization of diverse cell types from mesenchymal lineage cells, including MSCs (*Pdgfra*^+^*/Prrx1*^+^*/Ly6a*^+^/*Ly6e*^+^/*Cxcl12*^+^/*Adam12*^+^), chondroblast (*Tnc*^+^) and mature chondrocytes (*Col2a1*^+^/*Col10a1*^+^).


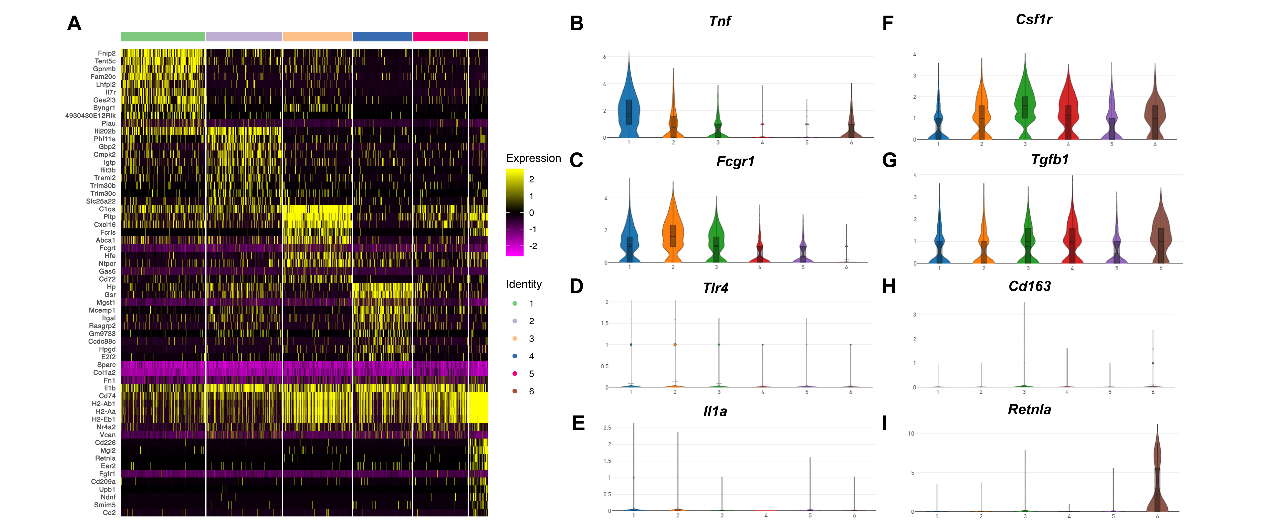


**Figure S9. Identification of the subtypes of macrophages in tibial muscle of *Nse-Bmp4* mice with or without injury.** (**A**) Heatmap showing the top 10 differentially expressed genes enriched among diverse types of macrophages in tibial muscle of *Nse*-*Bmp4* mice. (**B-G**) Violin plots showing the specific expression of M1 macrophages (*Tnf*, *Fcgr1*^+^/*Tlr4*^+^/*Il1a*^+^) and M2 macrophage (*Mrc1*^+^/*Csf1r*^+^/*Tgfb1*^+^/*Cd163*^+^/*Retnla*^+^) in tibial muscle of *Nse*-*Bmp4* mice.


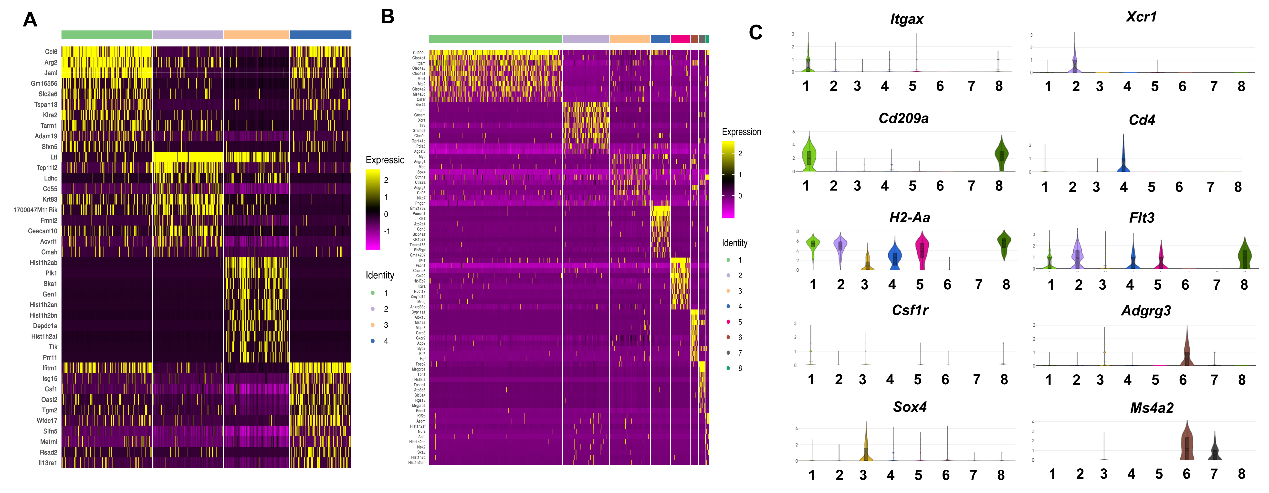


**Figure S10. Characterization of neutrophils and DCs in tibial muscle of *Nse-Bmp4* mice with or without injury.** (**A, B**) Heatmap showing the top 10 differentially expressed genes enriched among diverse types of neutrophils (**A**) and DCs (**B**) in tibial muscle of *Nse*-*Bmp4* mice. (**C**) Violin plots showing the specific expression of each type of DCs, including cDC1 (*Itgax*^+^/*Xcr1*^+^), cDC2 (*Itgax*^+^/*Cd209a*^+^), pDC (*Itgax*^+^/*Cd4*^+^), common DC progenitors (*Itgax*^-^/*H2-Aa*^+^/*Flt3*^+^/*Csf1r*^+^/*Adgrg3*^+^/*Sox4*^+^) and mast cells (*Ms4a2*^+^) in tibial muscle of *Nse*-*Bmp4* mice.


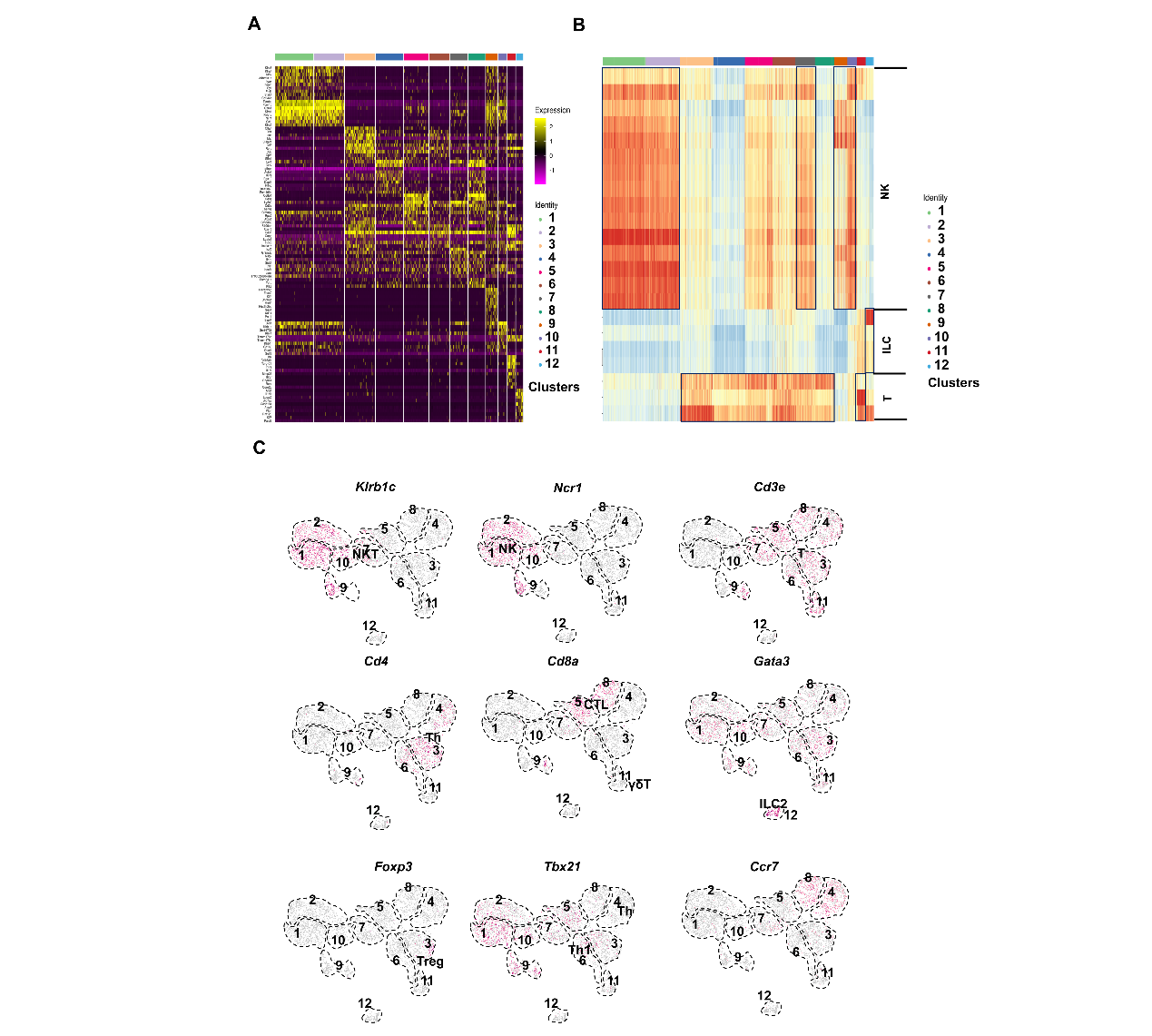


**Figure S11. Characterization of NK and T cells in tibial muscle of *Nse-Bmp4* mice with or without injury.** (**A**) Heatmap showing the top 10 differentially expressed genes enriched among diverse types of NK and T cells in tibial muscle of *Nse*-*Bmp4* mice. (**B**) Heatmap of genes associated with NK and T cells using Immgen database. (**C**) UMAP visualization of diverse cell types from NK and T cells, including NK cells (*Klrb1c*^+^/*Ncr1*^+^), CD4^+^ T cells (*Cd3e*^+^/*Cd4*^+^), CD8^+^ T cells (*Cd3e*^+^/*Cd8a*^+^), NKT cells (*Klrb1c*^+^/*Cd3e*^+^), γδT cells (*Cd3e*^+^/*Klrb1c*^-^/*Cd4*^-^/*Cd8a*^-^), and innate lymphoid cells 2 (*Gata3*^+^/*Klrb1c*^-^/*Cd3e*^-^)


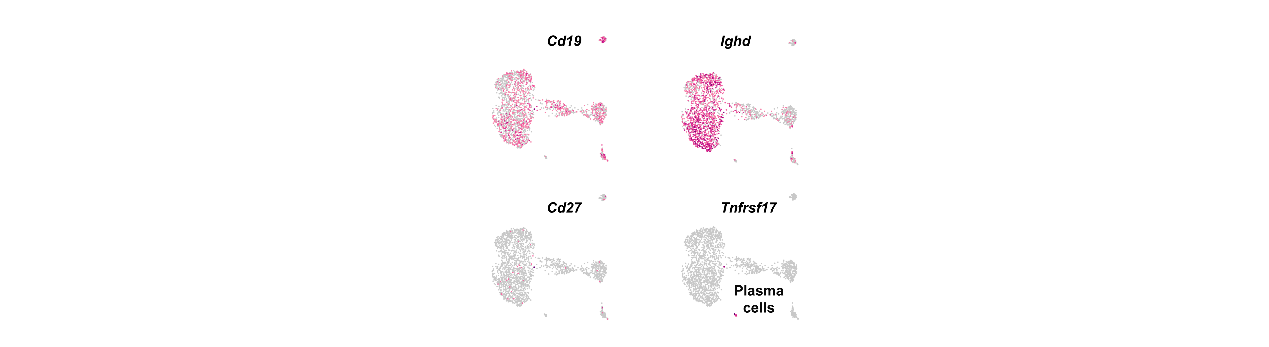


**Figure S12. UMAP visualization of diverse cell types from B cells**


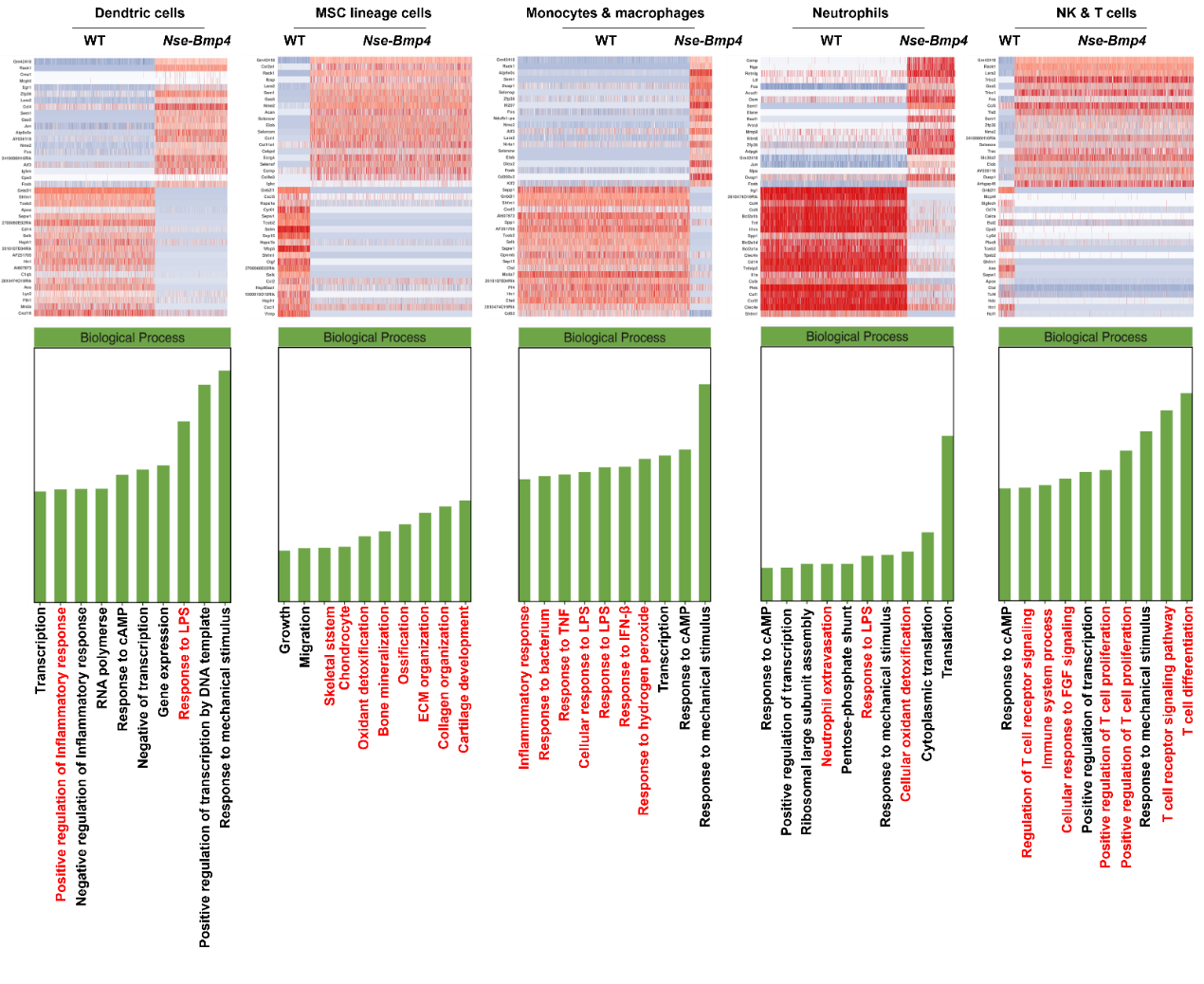


**Figure S13. GO analysis of immune cells and MSC lineage cells between HO model mice and normal mice.**


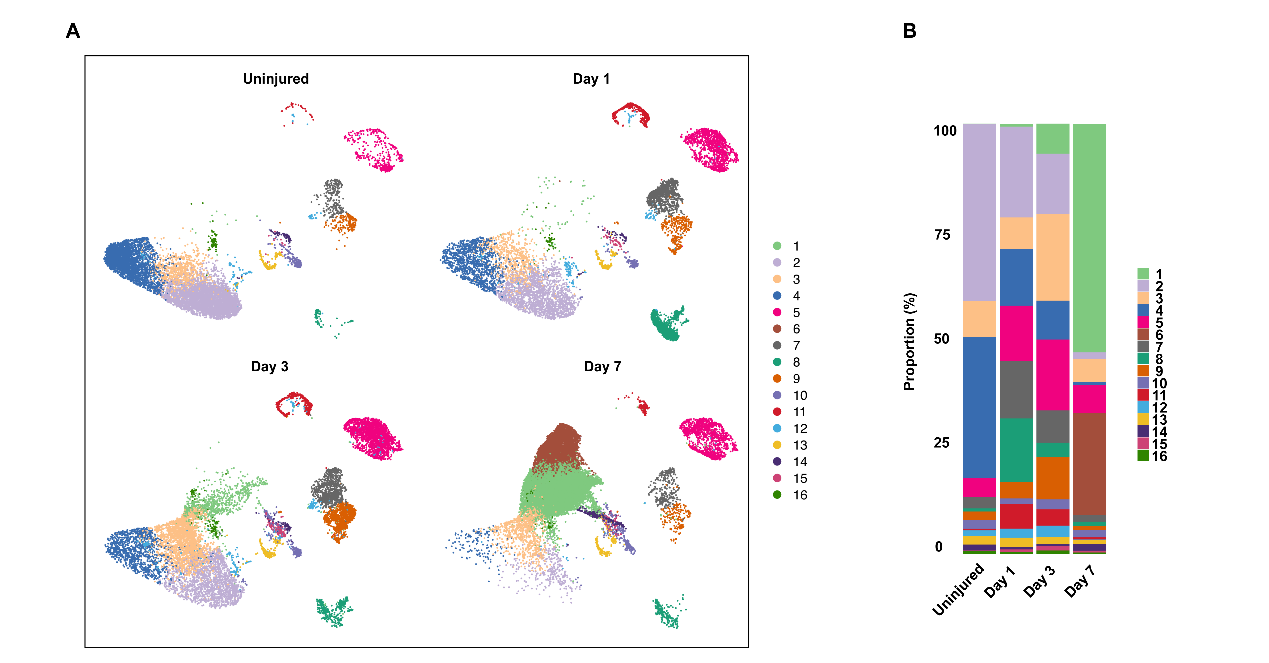


**Figure S14. MSC lineage cells, accompanied with immune cells were increased following tibial muscle injury.** (**A**) UMAP visualization of dynamic changes of each cell types in the uninjured and injured tibial muscle at 1, 3 and 7 dpi. (**B**) The percentage of each type of immune cells in the uninjured and injured tibial muscle at 1, 3 and 7 dpi.


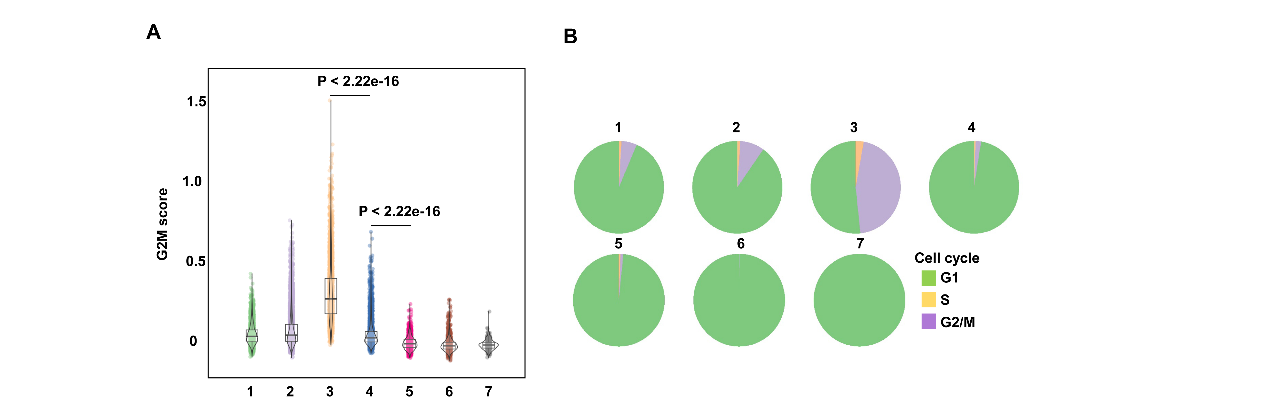


**Figure S15. Cell cycle analysis of MSC lineage cells.** (**A**) Addmodulescore analysis of G2M phase-associated genes for MSC lineage cells. (**B**) Scran cell cycle analysis for MSC lineage cells.


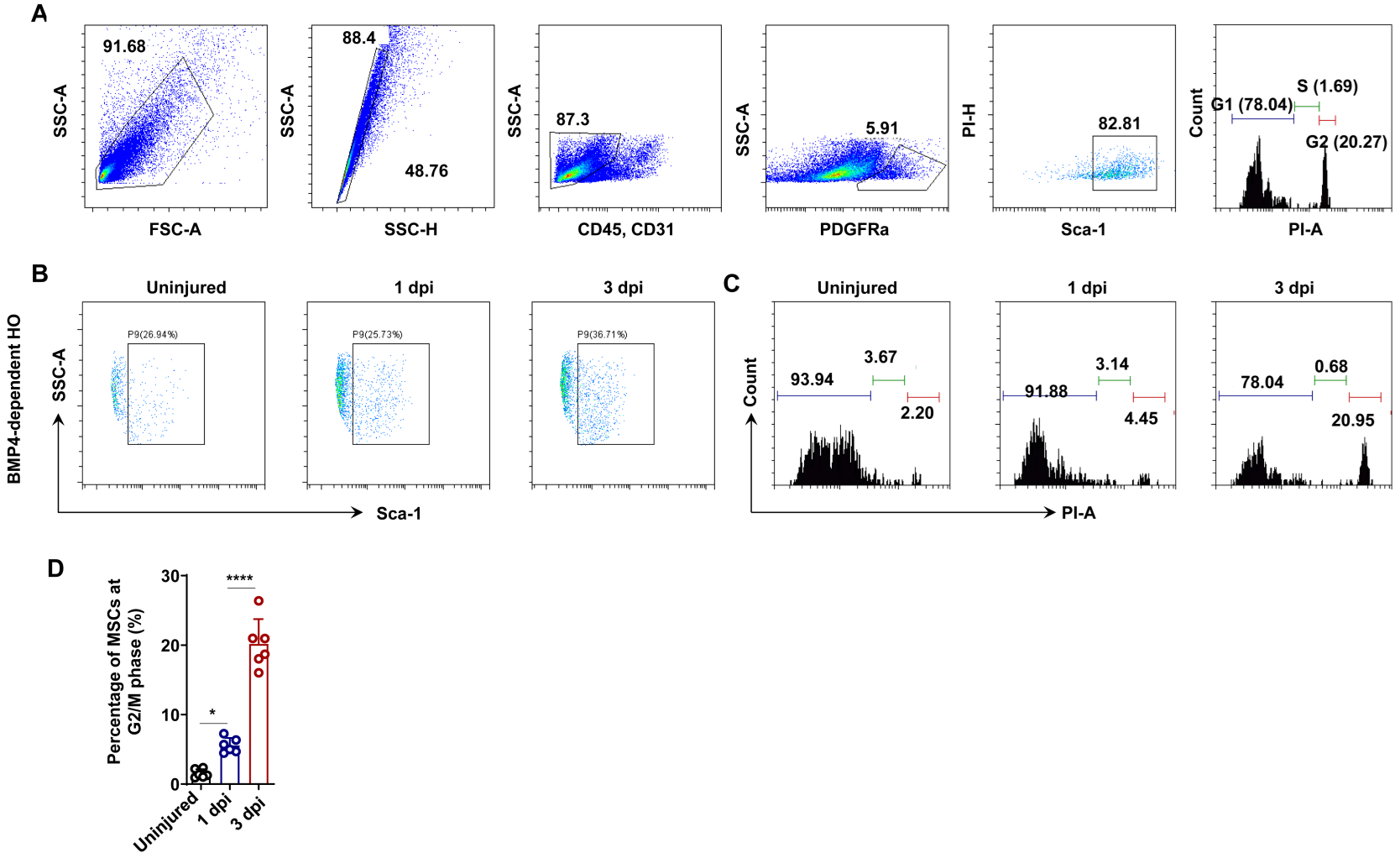


**Figure S16. The population of MSCs of tibial muscle were increased at 1 and 3 dpi compared to that in uninjured muscle of *Nse*-*Bmp4* mice. (A)** Gating strategy of for MSC and their cell cycle analysis. (**B**) Representative flow cytometry analysis for MSCs in uninjured tibial muscle and tibial muscle at 1 and 3 dpi. (**C**) Representative cell cycle analysis for MSCs in uninjured tibial muscle and tibial muscle at 1, 3 and 7 dpi. (**D**) Statistical analysis of percentage of MSCs at G2M phase in uninjured tibial muscle and injured tibial muscles (n=6 per group). Data are presented as mean ± SD of biological replicates. * *p* < 0.05, **** *p* < 0.0001.


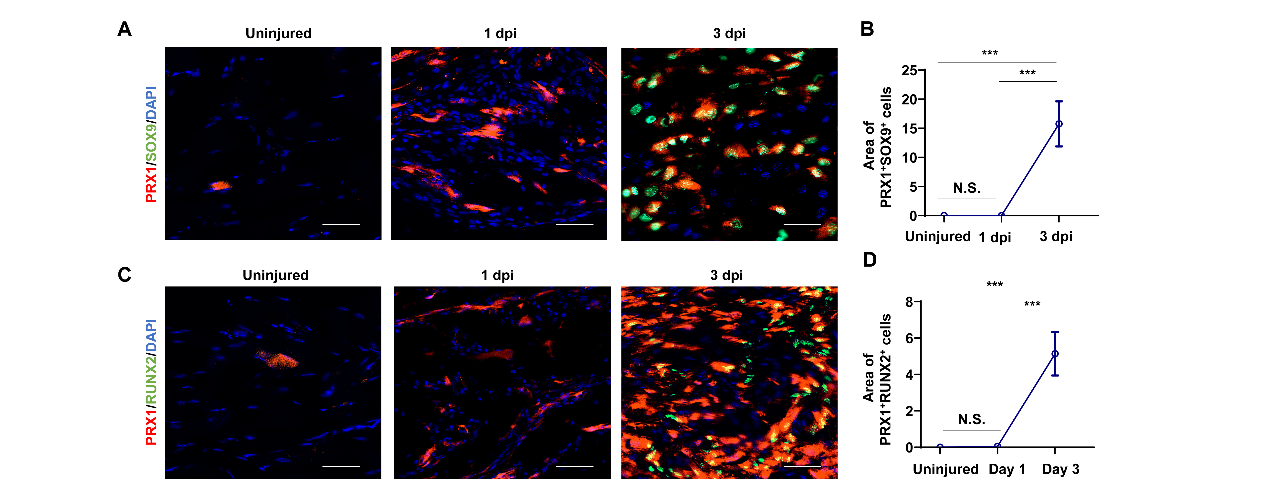
**Figure S17. *Prrx1*-expressing MSCs transitioned into osteochondral lineages.** (**A, B**) Representative IF images (**A**) and statistical analysis (**B**) of PRX1^+^/SOX9^+^ cells in tibial muscle of *Prrx1-Cre*; *Nse-Bmp4*; Ai9 mice at 0, 1 and 3 dpi. (**C, D**) Representative IF images (**C**) and statistical analysis (**D**) of PRX1^+^/RUNX2^+^ cells in tibial muscle of *Prrx1-Cre*; *Nse-Bmp4*; Ai9 mice at 0, 1 and 3 dpi (n=5 per group). Data are presented as mean ± SD of biological replicates. **** *p* < 0.0001.


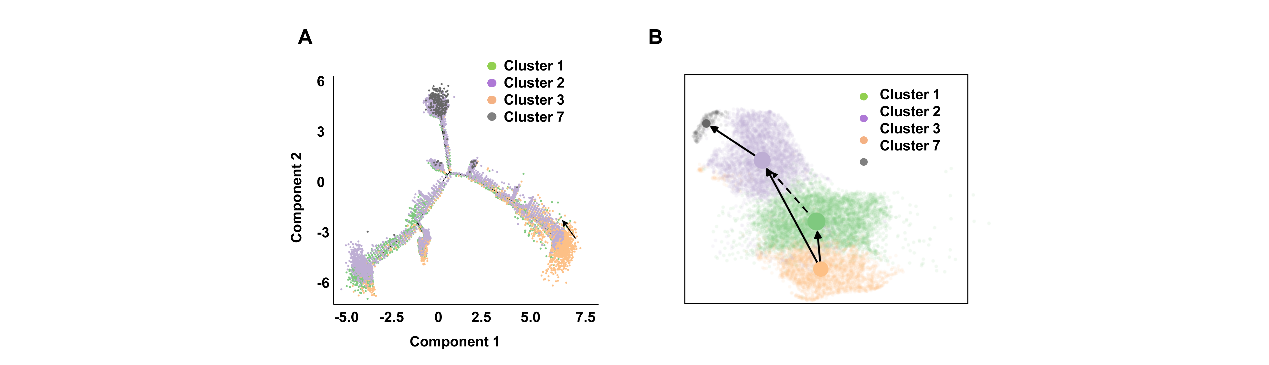
**Figure S18. cMSC can give rise to chondroblasts, which are the origin of mature chondrocytes.** (**A**) Pseudotemporal trajectories of the chondrocytes development (from cMSC to chondroblasts and eventually to mature chondrocytes) in injured tibial muscle of *Nse-Bmp4* mice using Monocle 2 analysis. (**B**) RNA velocity analyses revealed that cMSCs served as the original cellular source of chondroblasts and mature chondrocytes.


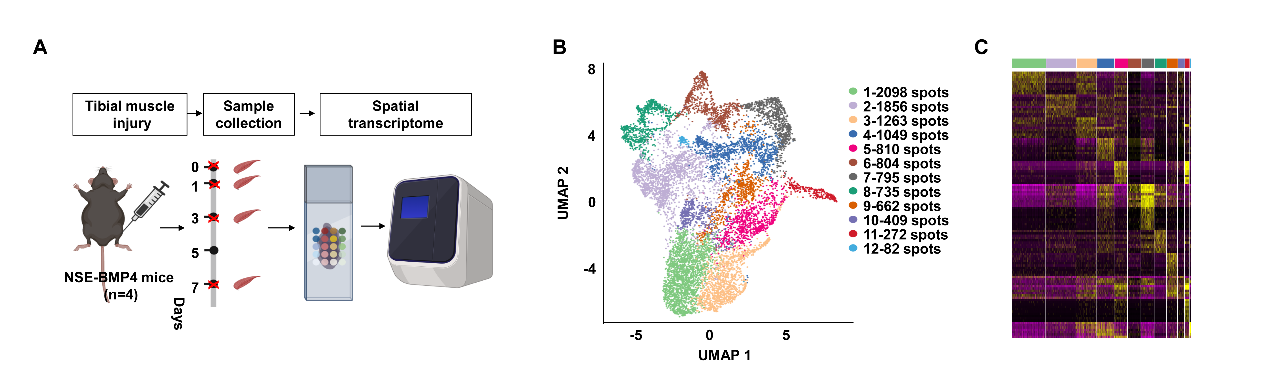
**Figure S19. Spatial transcriptomic analysis of the cell types in tibial muscle of *Nse*-*Bmp4* mice.** (**A)** Working flow for spatial transcriptome of uninjured and injured site of *Nse-Bmp4* mice at 0, 1, 3 and 7 dpi. (**B**) UMAP visualization of the spots containing various cell types in the HO lesion using spatial transcriptome sequencing. (**C**) Heatmap showing the top 10 differentially expressed genes enriched among diverse spots in tibial muscle of *Nse*-*Bmp4* mice.


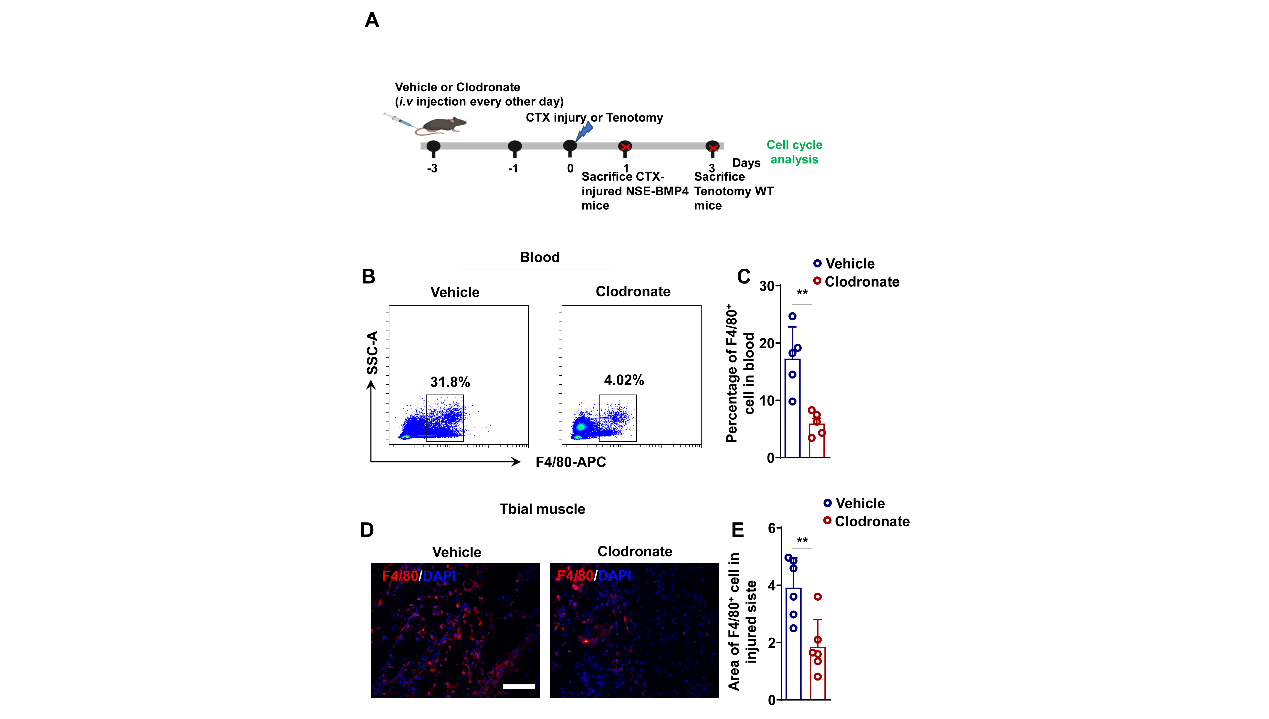


**Figure S20. Clodronate or MRC1 Abs effectively inhibited the infiltration of macrophages into the injured tibial muscle of *Nse*-*Bmp4* mice.** (**A)** Visual representation of our study design, highlighting the systematic process of clodronate consumption and collecting target tissue at specific time intervals for indicated analysis. (**B, C**) Representative flow cytometry (**B**) and statistical analysis (**C**) of macrophages in the blood of *Nse*-*Bmp4* mice with either vehicle or clodronate treatment (n=5 per group). Data are presented as mean ± SD of biological replicates. ** *p* < 0.01. (**D, E**) Representative flow cytometry (**D**) and statistical analysis (**E**) of macrophages in the injured tibial muscle of *Nse*-*Bmp4* mice with either vehicle or clodronate treatment (n=5 per group). Data are presented as mean ± SD of biological replicates. ** *p* < 0.0. Scale bar, 200μm.


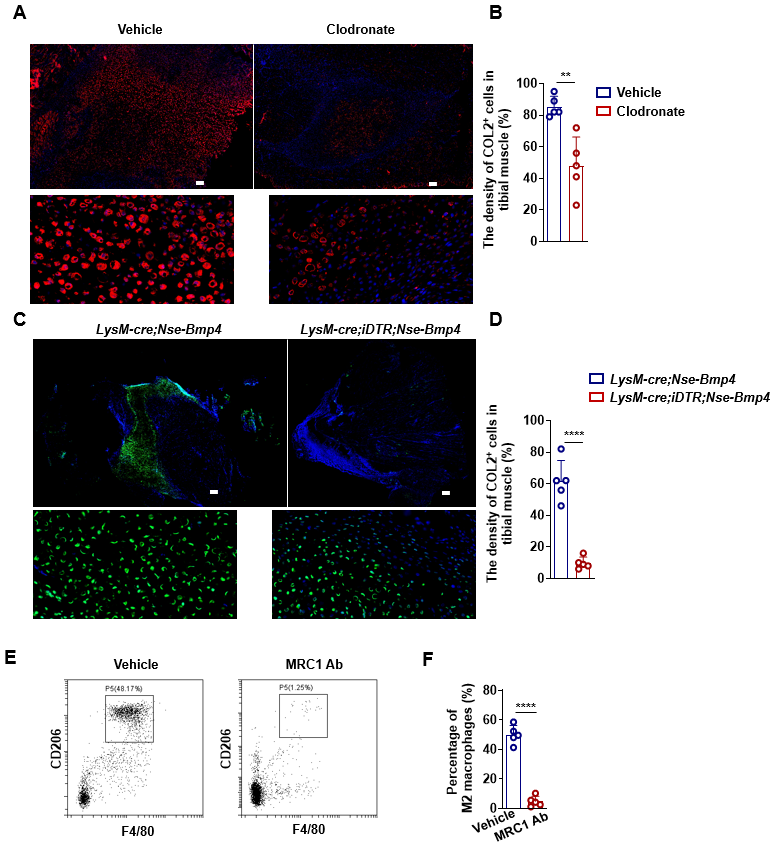


**Figure S21. Depletion of macrophages effectively inhibited the chondrocytes formation in injured tibial muscle of *Nse*-*Bmp4* mice.** (**A, B**) Representative IF staining images (**A**) and statistical analysis (**B**) of COL2^+^ cells in injured tibial muscle of *Nse*-*Bmp4* mice with either vehicle or clodronate treatment (n=5 per group). Data are presented as mean ± SD of biological replicates. ** *p* < 0.01 (unpaired two-tailed t-test). Scale bar, 200μm. (**C, D**) Representative flow cytometry (**C**) and statistical analysis (**D**) of COL2^+^ cells in the injured tibial muscle of *LysM*-*cre*; *Nse*-*Bmp4* and *LysM*-*cre*; *iDTR*; *Nse*-*Bmp4* mice (n=5 per group). Data are presented as mean ± SD of biological replicates. **** *p* < 0.0001. Scale bar, 200μm. (**E, F**) Representative flow cytometry (**E**) and statistical analysis (**F**) of M2 macrophages in the tibial muscle of *Nse*-*Bmp4* mice with either vehicle or MRC1 Abs treatment (n=5 per group). Data are presented as mean ± SD of biological replicates. **** *p* < 0.0001.


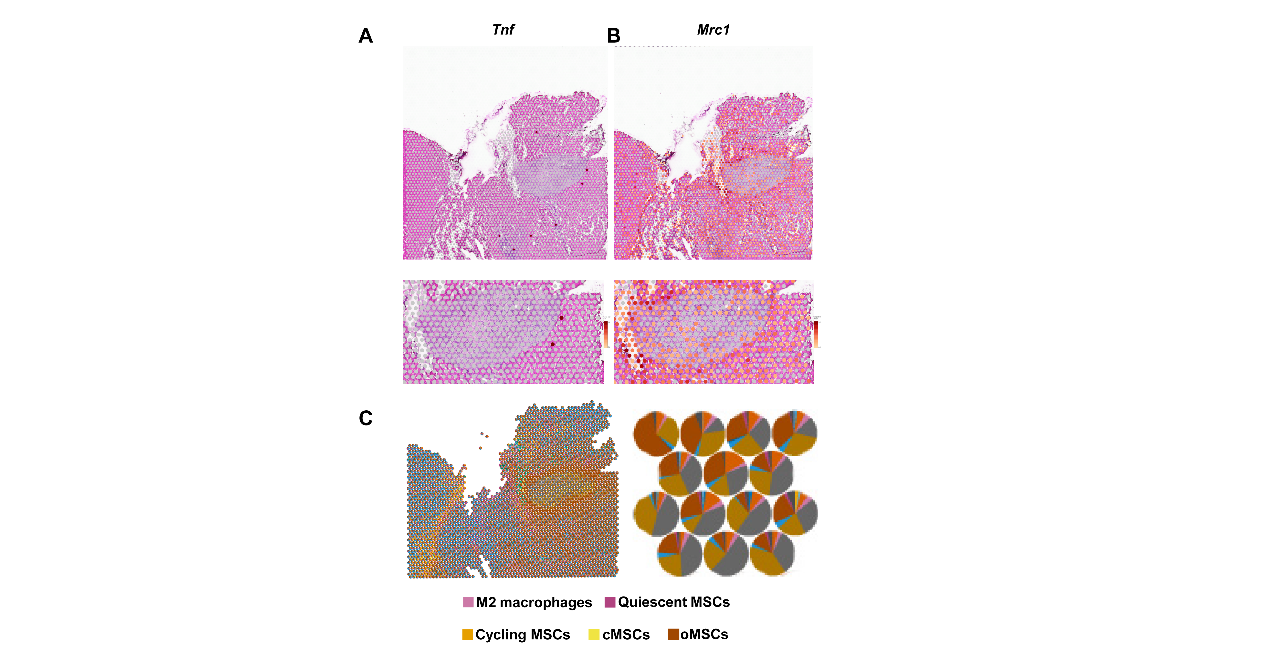


**Figure S22. M2 macrophages were distributed into peri-cartilage region of tibial muscle of *Nse*-*Bmp4* mice at 7 dpi.** (**A, B**) Spatial transcriptomic analysis of the expression of *Tnf* (**A**) and *Mrc1* (**B**) in HO lesion at 7 dpi (**C**) RCTD analysis of the distribution of M2 macrophages and each type of MSCs in HO lesion at 7 dpi.


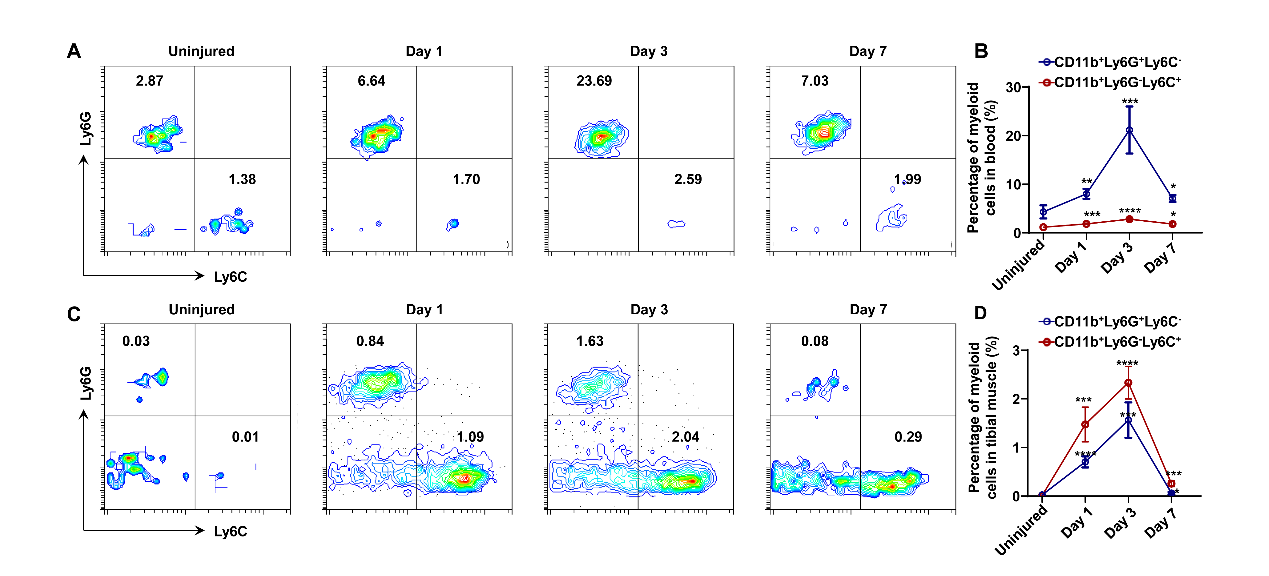


**Figure S23. Myeloid cells were increased after tibial muscle injury.** (**A, B**) Representative images of flow cytometry (**A**) and statistical analysis (**B**) of myeloid cells in blood of *Nse*-*Bmp4* mice at 0, 1, 3 and 7 dpi. (n=5 per group). Data are presented as mean ± SD of biological replicates. * *p* < 0.05.** *p* < 0.01.*** *p* < 0.001. **** *p* < 0.0001. (**C, D**) Representative images of flow cytometry (**C**) and statistical analysis (**D**) of myeloid cells in tibial muscle of *Nse*-*Bmp4* mice at 0, 1, 3 and 7 dpi. (n=5 per group). Data are presented as mean ± SD of biological replicates. *** *p* < 0.001, **** *p* < 0.0001.


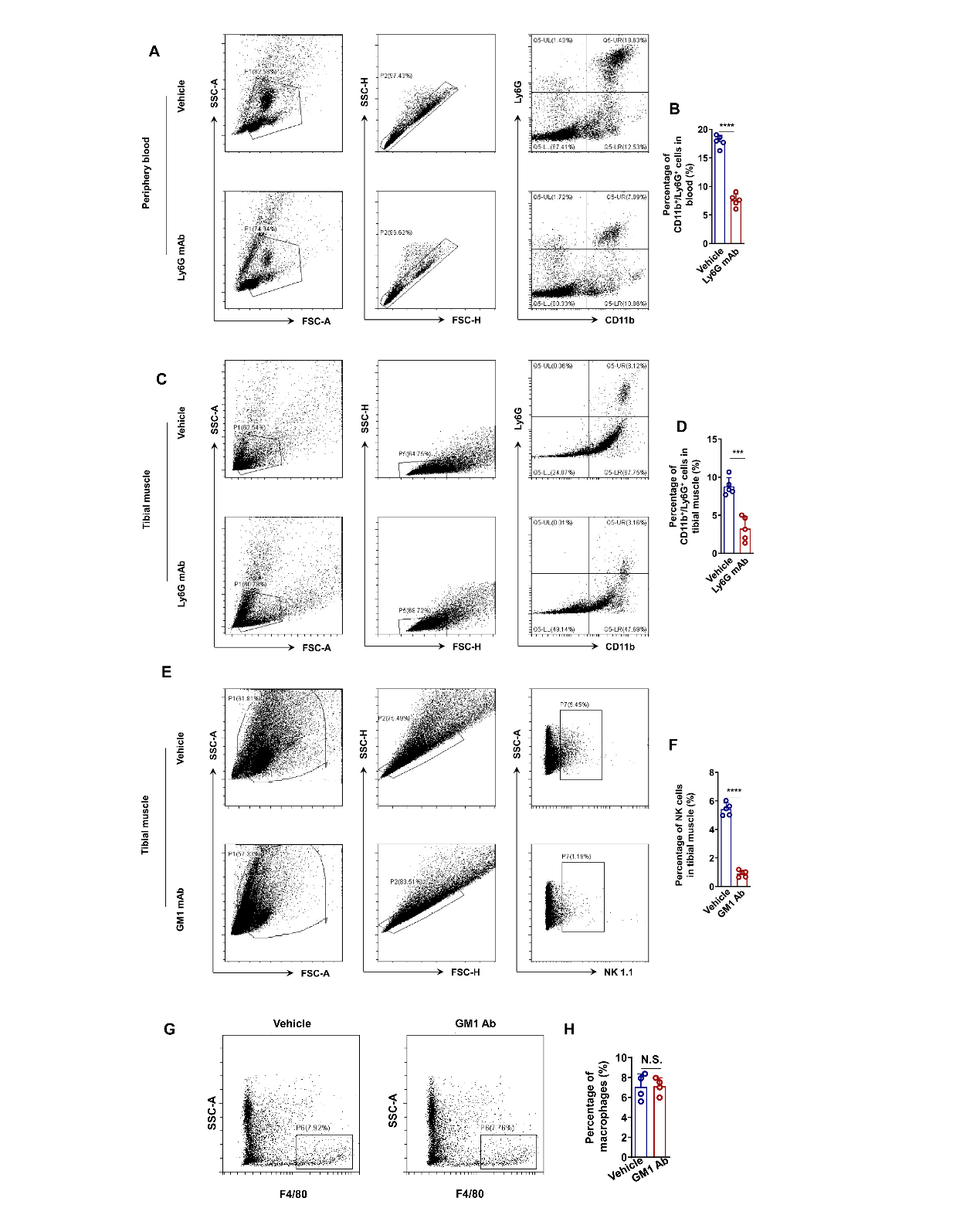


**Figure S24. Anti-Ly6G Abs and Asialo GM1 Abs effectively inhibited the production of neutrophils and NK cells, respectively, in *Nse*-*Bmp4* mice after tibial muscle injury.** (**A, B**) Representative images of flow cytometry (**A**) and statistical analysis (**B**) of neutrophils in blood of *Nse*-*Bmp4* mice with or without Ly6G Abs treatment at indicated time point. (n=5 per group). Data are presented as mean ± SD of biological replicates. **** *p* < 0.0001. (**C, D**) Representative images of flow cytometry (**C**) and statistical analysis (**D**) of neutrophils in tibial muscle of *Nse*-*Bmp4* mice with or without Ly6G Abs treatment at indicated time point. (n=5 per group). Data are presented as mean ± SD of biological replicates. **** *p* < 0.0001. (**E, F**) Representative images of flow cytometry (**E**) and statistical analysis (**F**) of NK cells in tibial muscle of *Nse*-*Bmp4* mice with or without Ly6G Abs treatment at indicated time point. (n=5 per group). Data are presented as mean ± SD of biological replicates. **** *p* < 0.0001. (**G, H**) Representative images of flow cytometry (**G**) and statistical analysis (**H**) of macrophages in tibial muscle of *Nse*-*Bmp4* mice with or without Asialo GM1 Abs treatment at indicated time point. (n=5 per group). Data are presented as mean ± SD of biological replicates. N.S. indicated no significance.


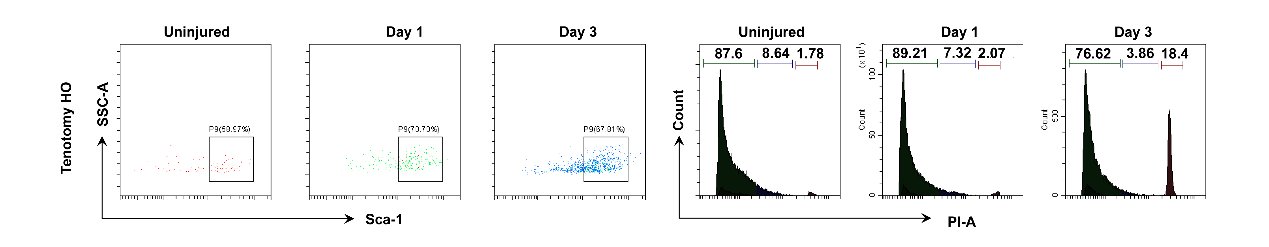
**Figure S25. Gating strategy of flow cytometry for MSCs and their cell cycle analysis.**


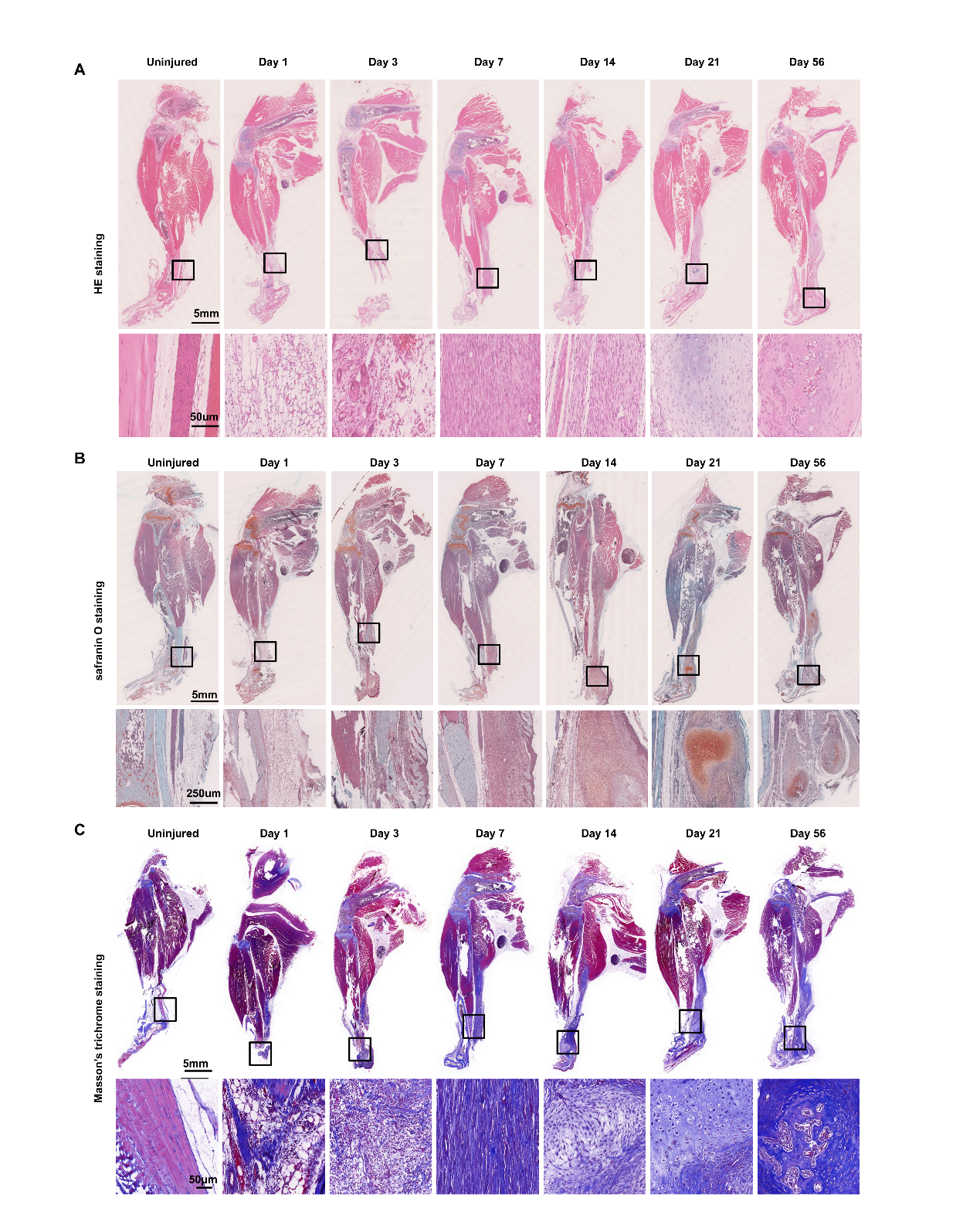
**Figure S26. Histologic analysis of the HO development in tendon of WT mice following tenotomy surgery**. (**A**) Representative HE staining images of uninjured and injured tendon of WT mice at different time points post injury (i.e., 1, 3, 7, 14, 21 and 56 dpi). (**B**) Representative Safranin O and fast green staining images of uninjured and injured tibial muscle of *Nse-Bmp4* mice at different time points post injury (i.e., 1, 3, 7, 14, 21 and 56 dpi). (**C**) Representative Masson staining images of uninjured and injured tibial muscle of *Nse-Bmp4* mice at different time points post injury (i.e., 1, 3, 7, 14, 21 and 56 dpi).


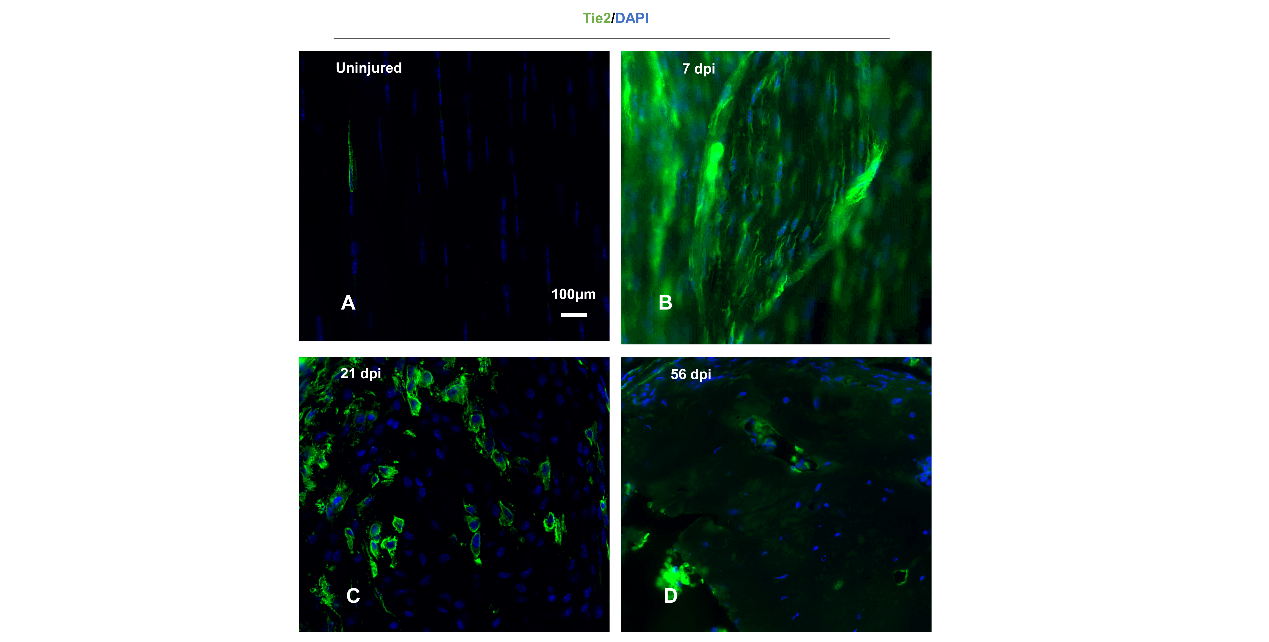
**Figure S27. Genetic tracing analysis of Tie2^+^ cells in tenotomy HO.**


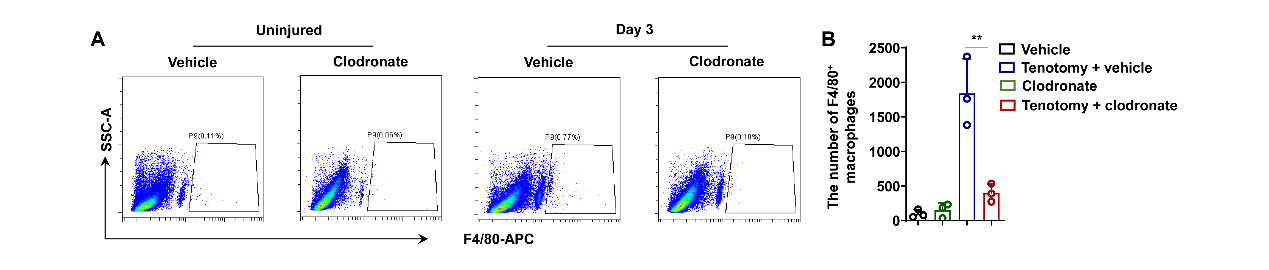
**Figure S28. Clodronate effectively decreased the production of macrophages in injury tendon of tenotomy HO model mice.** (**A, B**) Representative images of flow cytometry (**A**) and statistical analysis (**B**) of macrophages in tendon of tenotomy mice at indicated time point. (n=5 per group). Data are presented as mean ± SD of biological replicates. **** *p* < 0.0001.


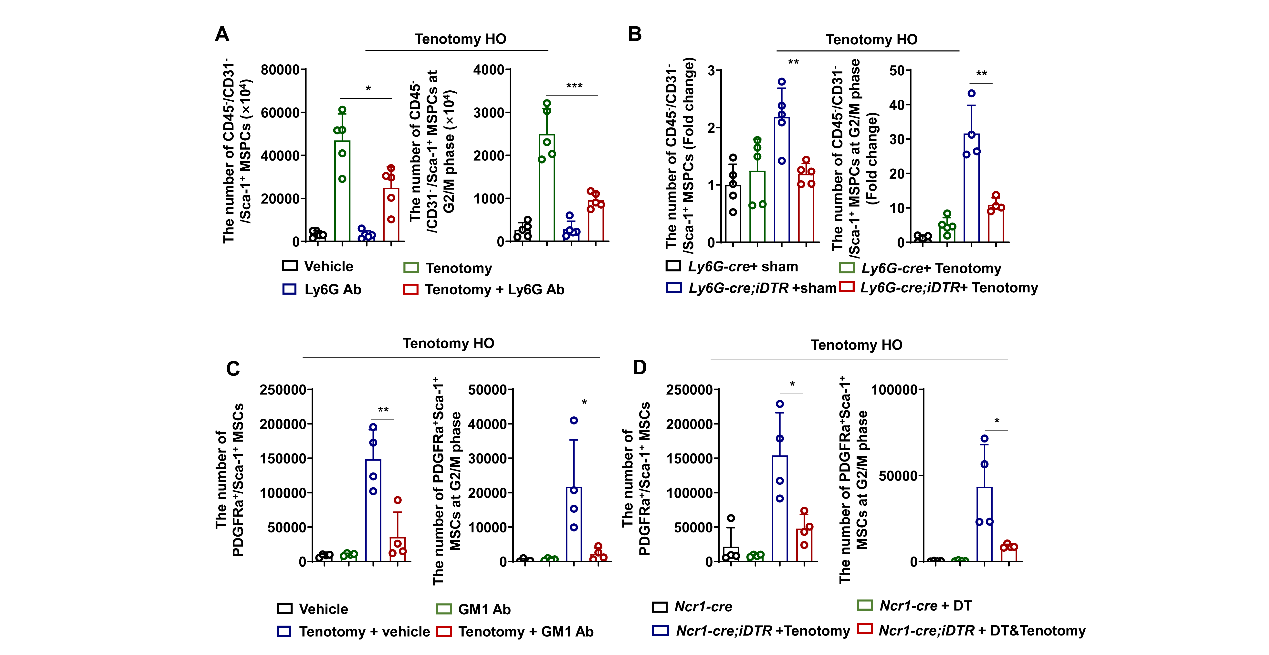
**Figure S29. Depletion of neutrophils or NK cells significantly inhibited the proliferation of MSC in tendon following injury.** (**A, B**) Statistical analysis of total MSCs and G2/M phase MSCs in injured tendon after chemically (Ly6G Abs, **A**) and genetically (*Ly6G*-*cre*; *iDTR*, **B**) induced neutrophils depletion. (n=5 per group). Data are presented as mean ± SD of biological replicates. **** *p* < 0.0001. (**C, D**) Statistical analysis of total MSCs and G2/M phase MSCs in injured tendon after chemically (GM1 Abs, **C**) and genetically (*Ncr1*-*cre*; *iDTR*, **D**) induced NK cells depletion. (n=5 per group). Data are presented as mean ± SD of biological replicates. **** *p* < 0.0001.


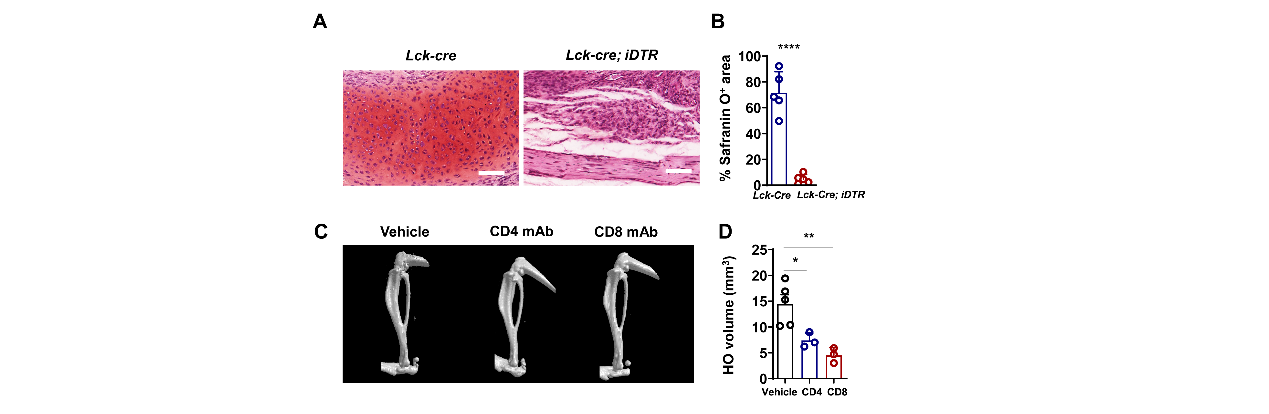
**Figure S30. Depletion of T cells impair the ectopic cartilage and bone formation in tenotomy HO model mice.** (**A, B**) Representative images of safranine O staining (**A**) and statistical analysis (**B**) of chondrocytes region in tendon of *Lck-cre* and *Lck-cre; iDTR* mice at 3 weeks post injury. (n=5 per group). Data are presented as mean ± SD of biological replicates. **** *p* < 0.0001. Scale bar, 200μm. (**C, D**) Representative images of microCT (**C**) and statistical analysis (**D**) of HO volume in tendon of mice with vehicle or CD4 mAb or CD8 mAb. (n=5 per group). Data are presented as mean ± SD of biological replicates. * *p* < 0.05, ** *p* < 0.01.


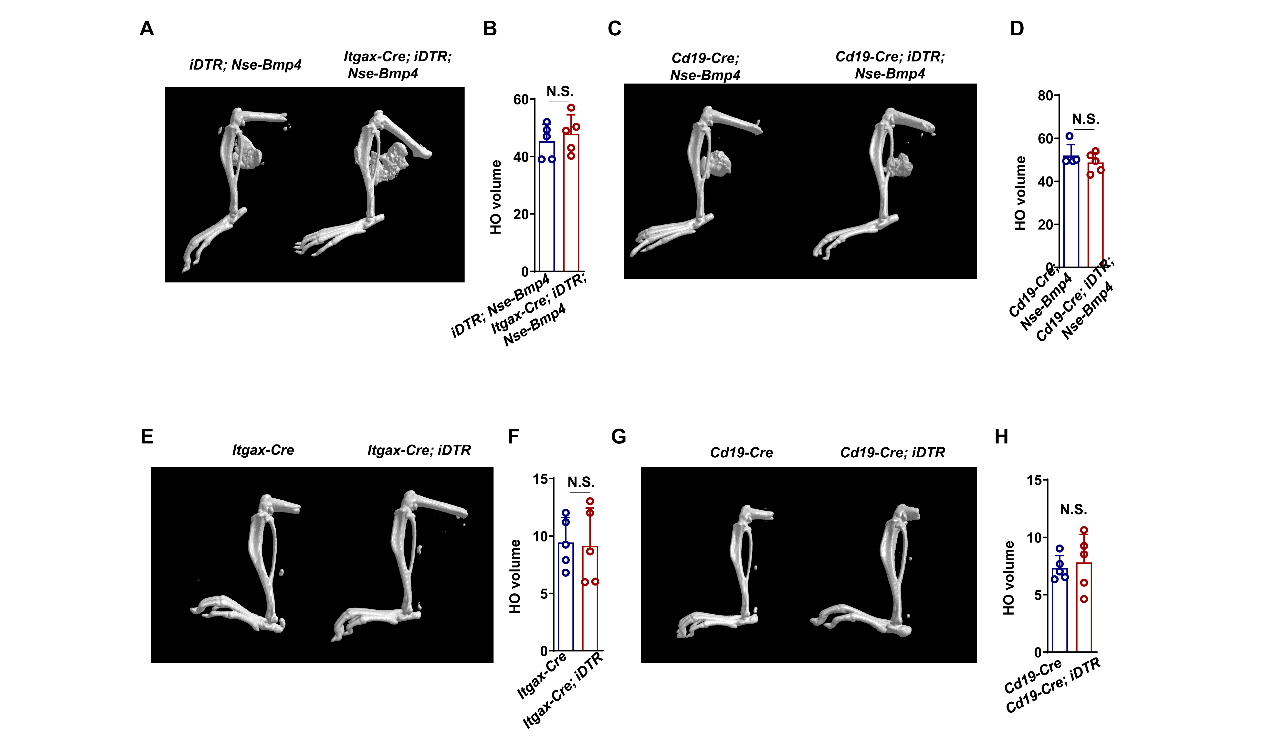
**Figure S31. Depletion of DCs or B cells did not impair the BMP4-dependent and tenotomy HO formation.** (**A, B**) Representative images of microCT (**A**) and statistical analysis (**B**) of HO volume in tibial muscle of *iDTR; Nse-Bmp4* and *Itgax-cre; iDTR; Nse-Bmp4* mice at indicated time point. (n=5 per group). Data are presented as mean ± SD of biological replicates. **** *p* < 0.0001. (**C, D**) Representative images of microCT (**C**) and statistical analysis (**D**) of HO volume in tibial muscle of *Cd19-cre; Nse-Bmp4* and *Cd19-cre; iDTR; Nse-Bmp4* mice at indicated time point. (n=5 per group). Data are presented as mean ± SD of biological replicates. **** *p* < 0.0001. (**E, F**) Representative images of microCT (**E**) and statistical analysis (**F**) of HO volume in injured tendon of *Itgax-cre* and *Itgax-cre; iDT* mice at indicated time point. (n=5 per group). Data are presented as mean ± SD of biological replicates. N.S. indicated no significance. (**G, H**) Representative images of microCT (**G**) and statistical analysis (**H**) of HO volume in injured tendon of *Cd19-cre* and *Cd19-cre; iDTR* mice at indicated time point. (n=5 per group). Data are presented as mean ± SD of biological replicates. N.S. indicated no significance.


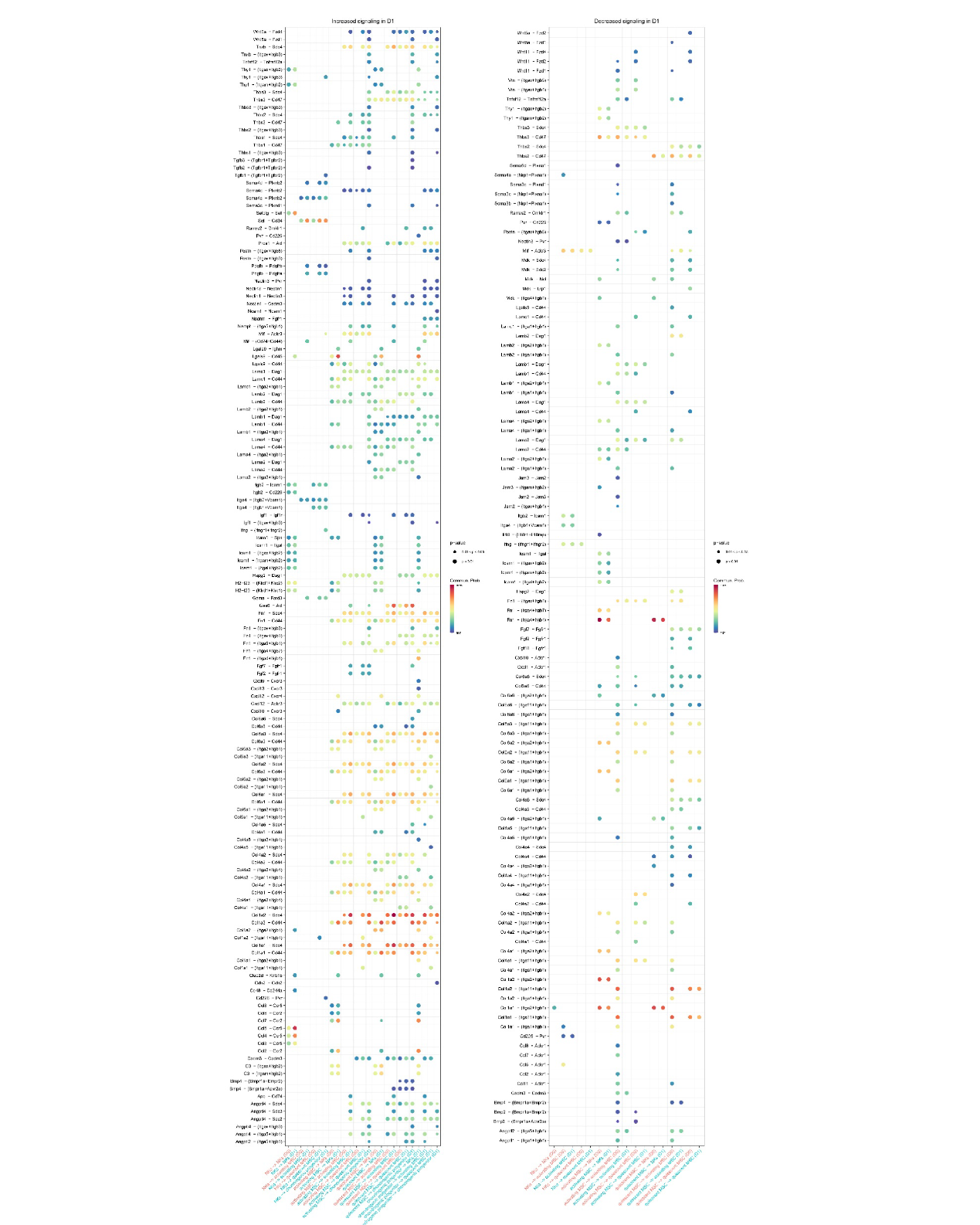


**Figure S32. Cellchat analysis of immune-MSC interaction at 1 dpi.**


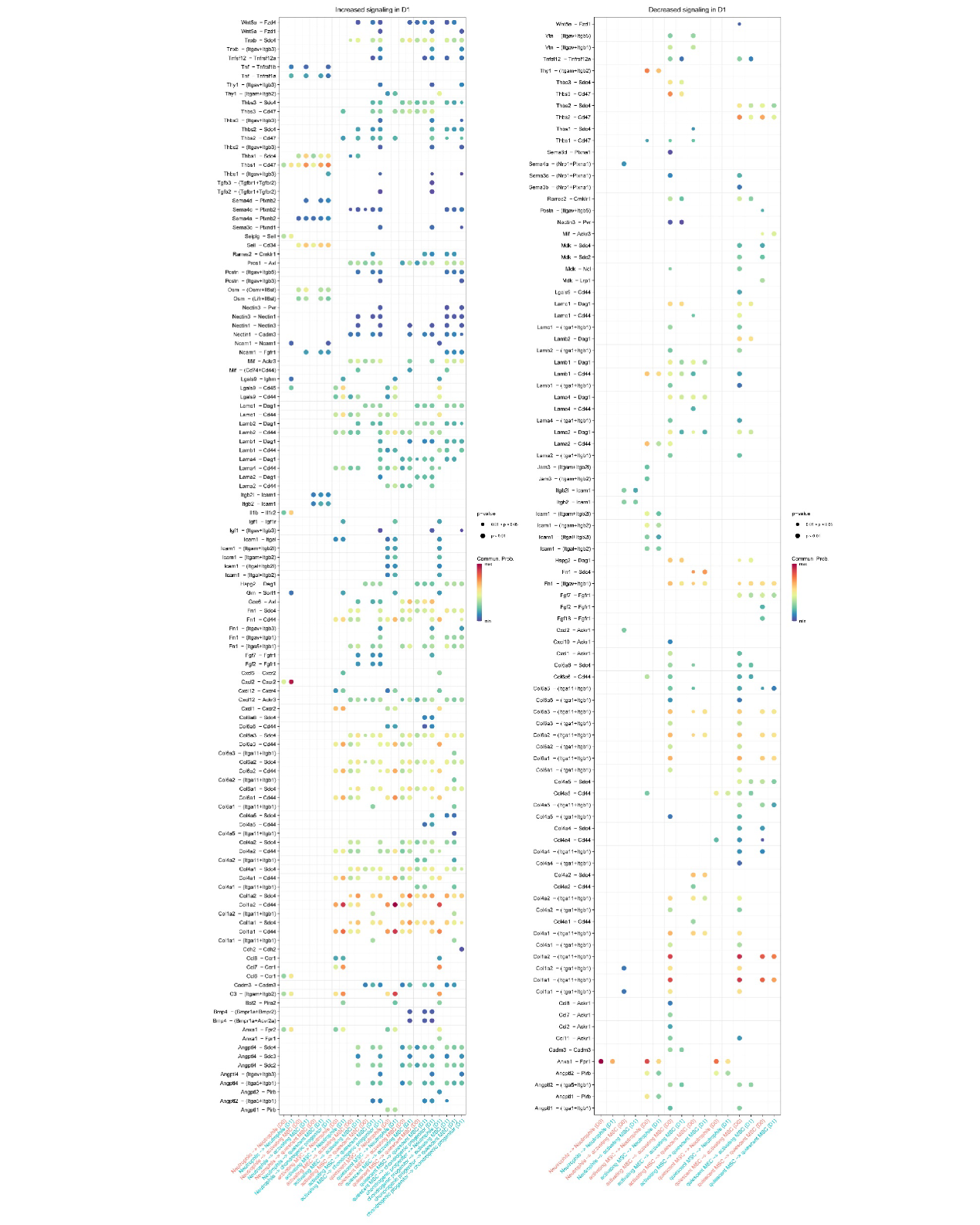
**Figure S33. Cellchat analysis of MSC-immune interaction at 1 dpi.**


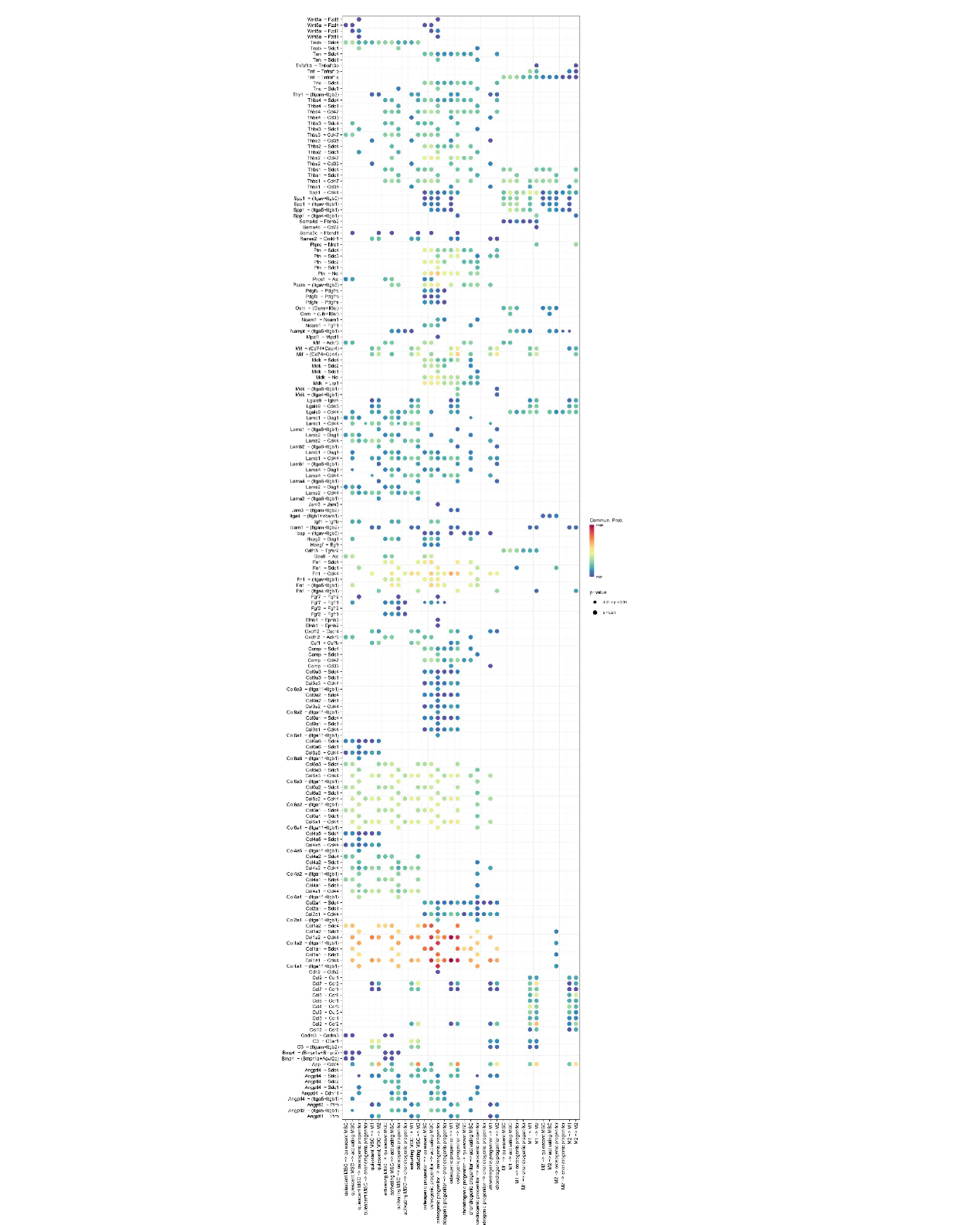
**Figure S34. Cellchat analysis of immune-MSC interaction in injured site of *Nse*-*Bmp4* mice over a course of 7 days.**


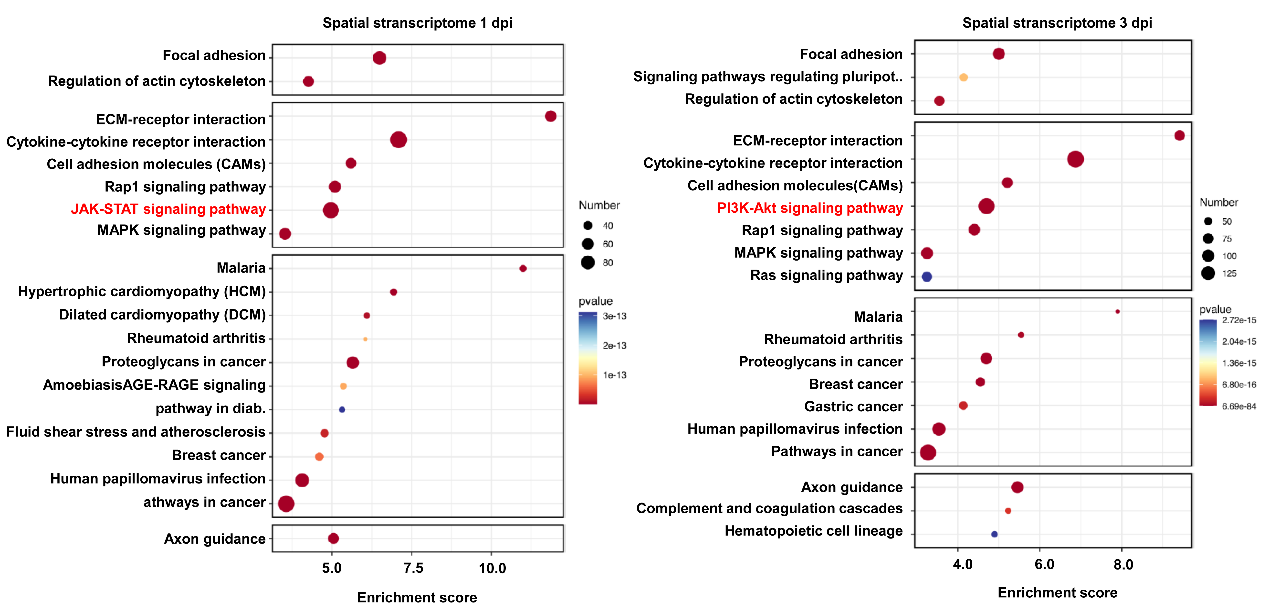
**Figure S35. KEGG analysis of immune-MSC interaction using spatial transcriptome.**
